# Transdiagnostic Ecological Momentary Intervention for Improving Self-Esteem in Youth Exposed to Childhood Adversity: The SELFIE Randomized Clinical Trial

**DOI:** 10.1001/jamapsychiatry.2023.4590

**Published:** 2023-11-29

**Authors:** Ulrich Reininghaus, Maud Daemen, Mary Rose Postma, Anita Schick, Iris Hoes-van der Meulen, Nele Volbragt, Dorien Nieman, Philippe Delespaul, Lieuwe de Haan, Marieke van der Pluijm, Josefien Johanna Froukje Breedvelt, Mark van der Gaag, Ramon Lindauer, Jan R. Boehnke, Wolfgang Viechtbauer, David van den Berg, Claudi Bockting, Therese van Amelsvoort

**Affiliations:** 1 Central Institute of Mental Health, Department of Public Mental Health, Medical Faculty Mannheim, University of Heidelberg, Mannheim, Germany; 2 Centre for Epidemiology and Public Health, Institute of Psychiatry, Psychology & Neuroscience, Health Service and Population Research Department, King’s College London, London, United Kingdom; 3 Department of Psychiatry and Neuropsychology, School for Mental Health and Neuroscience, Maastricht University, Maastricht, the Netherlands; 4 Mondriaan Mental Health Centre, Maastricht, the Netherlands; 5 Department of Child and Adolescent Psychiatry, Amsterdam UMC, location Academic Medical Center, Amsterdam, the Netherlands; 6 Levvel, Academic Centre for Child and Adolescent Psychiatry, Amsterdam, the Netherlands; 7 Prodeba Mental Health Care, Leiden, the Netherlands; 8 Department of Psychiatry, Amsterdam University Medical Centers (location AMC), Amsterdam Public Health, Amsterdam, the Netherlands; 9 Centre for Urban Mental Health, University of Amsterdam, Amsterdam, the Netherlands; 10 Department of Clinical Psychology, VU University, Amsterdam, the Netherlands; 11 School of Health Sciences, University of Dundee, Dundee, United Kingdom; 12 Department of Psychosis research, Parnassia Psychiatric Institute, The Hague, the Netherlands; 13 Koraal, YiP, Urmond, the Netherlands; 14 Department of Child and Adolescent Psychiatry, Institute of Psychiatry, Psychology and Neuroscience, King’s College London, London, United Kingdom

## Abstract

**Importance:**

Targeting low self-esteem in youth exposed to childhood adversity is a promising strategy for preventing adult mental disorders. Ecological momentary interventions (EMIs) allow for the delivery of youth-friendly, adaptive interventions for improving self-esteem, but robust trial-based evidence is pending.

**Objective:**

To examine the efficacy of SELFIE, a novel transdiagnostic, blended EMI for improving self-esteem plus care as usual (CAU) compared with CAU only.

**Design, Setting, and Participants:**

This was a 2-arm, parallel-group, assessor-blinded, randomized clinical trial conducted from December 2018 to December 2022. The study took place at Dutch secondary mental health services and within the general population and included youth (aged 12-26 years) with low self-esteem (Rosenberg Self-Esteem Scale [RSES] <26) exposed to childhood adversity.

**Interventions:**

A novel blended EMI (3 face-to-face sessions, email contacts, app-based, adaptive EMI) plus CAU or CAU only.

**Main Outcomes and Measures:**

The primary outcome was RSES self-esteem at postintervention and 6-month follow-up. Secondary outcomes included positive and negative self-esteem, schematic self-beliefs, momentary self-esteem and affect, general psychopathology, quality of life, observer-rated symptoms, and functioning.

**Results:**

A total of 174 participants (mean [SD] age, 20.7 [3.1] years; 154 female [89%]) were included in the intention-to-treat sample, who were primarily exposed to childhood emotional abuse or neglect, verbal or indirect bullying, and/or parental conflict. At postintervention, 153 participants (87.9%) and, at follow-up, 140 participants (80.5%), provided primary outcome data. RSES self-esteem was, on average, higher in the experimental condition (blended EMI + CAU) than in the control condition (CAU) across both postintervention and follow-up as a primary outcome (B = 2.32; 95% CI, 1.14-3.50; *P* < .001; Cohen *d*-type effect size [hereafter, Cohen *d*] = 0.54). Small to moderate effect sizes were observed suggestive of beneficial effects on positive (B = 3.85; 95% CI, 1.83-5.88; *P* < .001; Cohen *d* = 0.53) and negative (B = −3.78; 95% CI, −6.59 to −0.98; *P* = .008; Cohen *d* = −0.38) self-esteem, positive (B = 1.58; 95% CI, 0.41-2.75; *P* = .008; Cohen *d* = 0.38) and negative (B = −1.71; 95% CI, −2.93 to −0.48; *P* = .006; Cohen *d* = −0.39) schematic self-beliefs, momentary self-esteem (B = 0.29; 95% CI, 0.01-0.57; *P* = .04; Cohen *d* = 0.24), momentary positive affect (B = 0.23; 95% CI, 0.01-0.45; *P* = .04; Cohen *d* = 0.20), momentary negative affect (B = −0.33; 95% CI, −0.59 to −0.03, *P* = .01, Cohen *d* = −0.27), general psychopathology (B = −17.62; 95% CI, −33.03 to −2.21; *P* = .03; Cohen *d* = −0.34), and quality of life (B = 1.16; 95% CI, 0.18-2.13; *P* = .02; Cohen *d* = 0.33) across postintervention and follow-up. No beneficial effects on symptoms and functioning were observed.

**Conclusions and Relevance:**

A transdiagnostic, blended EMI demonstrated efficacy on the primary outcome of self-esteem and signaled beneficial effects on several secondary outcomes. Further work should focus on implementing this novel EMI in routine public mental health provision.

**Trial Registration:**

Dutch Trial Register Identifier:NL7129(NTR7475)

## Key Points

**Question:**

Can a transdiagnostic, blended ecological momentary intervention improve self-esteem in youth with low self-esteem and prior exposure to childhood adversity?

**Findings:**

This randomized clinical trial of 174 youth with low self-esteem and exposure to childhood adversity demonstrated that a novel transdiagnostic ecological momentary intervention improved the primary outcome of self-esteem at postintervention and 6-month follow-up. Small to moderate effect sizes signaled beneficial effects on general psychopathology, quality of life, and other secondary outcomes.

**Meaning:**

This trial demonstrated efficacy of a novel transdiagnostic ecological momentary intervention on self-esteem and signaled beneficial effects on secondary outcomes, which needs to be followed by investigating implementation in routine public mental health provision.

## Teaser

This randomized clinical trial investigates the efficacy of a transdiagnostic ecological momentary intervention for improving self-esteem in youth with low self-esteem and exposure to childhood adversity.

## Introduction

Most mental disorders first emerge in adolescence and early adulthood and, as such, contribute substantially to disease burden.^1,2,3^ Recent years have seen a shift in focus toward transdiagnostic frameworks, which broadly posit that early clinical phenotypes are nonspecific^4,5^ and may result in a range of mental disorders later in life.^5,6^ Childhood adversity (ie, abuse, neglect, bullying, parental conflict) is one of the most pervasive risk factors for various mental disorders.^7,8,9,10,11^ Although primary prevention of childhood adversity continues to be of prime importance, it remains difficult to achieve for all, and, hence, interventions targeting the negative consequences of childhood adversity in youth are a promising selective prevention strategy for adult mental disorders with tangible public health implications.^12,13^ One important candidate mechanism that may link childhood adversity to mental ill health is low self-esteem.^14,15^ Indeed, childhood adversity may increase risk of later psychopathology via pathways through low self-esteem.^13,14,16,17,18,19,20,21,22,23^ This is corroborated by evidence that momentary self-esteem, measured with Ecological Momentary Assessment (EMA), may play an important role in pathways to adult mental ill health.^17,23,24,25,26^

The SELFIE intervention, a novel, transdiagnostic ecological momentary intervention (EMI), adopts a strategy that aims to transform evidence on self-esteem as a candidate momentary mechanism identified in EMA studies to real-time EMIs targeting this mechanism in daily life. EMIs offer a unique opportunity for real-time tailoring of interventions to what individuals need in a given moment and context.^27,28,29,30^ Consistent with an ecological interventionist causal model approach,^30,31^ EMIs reflect a very promising strategy for preventing adult mental disorders and/or improving well-being, resilience, and outcomes in youth already in contact with services for early mental health problems by targeting momentary self-esteem in their daily life. However, robust trial-based evidence on EMIs remains very limited.^28,30,32,33^

This multicenter randomized clinical trial (RCT) aimed to test the efficacy of SELFIE, a novel transdiagnostic, blended EMI for improving self-esteem in youth aged 12 to 26 years with prior exposure to childhood adversity. First, we hypothesized that, compared with the control condition (care as usual [CAU]), self-esteem will, on average, be higher in the experimental condition (blended EMI + CAU) across postintervention and 6-month follow-up (primary outcome). Second, we hypothesized that, compared with the control condition (CAU), momentary self-esteem, positive self-esteem, positive schematic self-beliefs, emotional well-being, momentary resilience, momentary positive affect, functioning, and subjective quality of life will, on average, be higher, and negative self-esteem, negative schematic self-beliefs, momentary negative affect, psychological distress, general psychopathology, and clinical symptoms (secondary outcomes) will be lower in the experimental condition (blended EMI + CAU).

## Methods

### Study Design

In a definitive, 2-arm, parallel-group, assessor-blinded, multicenter RCT, individuals aged between 12 and 26 years with prior exposure to childhood adversity and low self-esteem were randomly allocated to SELFIE in addition to CAU (experimental condition) or CAU only (control condition). Recruitment for this trial started in December 2018 and outcome assessment at 6-month follow-up was completed in December 2022. Recruitment was from secondary mental health services in collaborating centers of 3 regions in the Netherlands (Noord-Holland [Amsterdam University Medical Center, Levvel], Zuid-Holland [Parnassia, Prodeba], Limburg [Mondriaan, Lionarons, Koraal]) and from the Dutch general population (via social media, schools, social services, flyers). The study was approved by the local ethics committee and followed the Consolidated Standards of Reporting Trials (CONSORT) reporting guideline.^34,35^ All participants provided written informed consent. The full trial protocol has been reported elsewhere (Supplement 1).^12^

### Participants

Participant eligibility criteria were as follows: (1) aged between 12 and 26 years,^36^ (2) exposure to childhood adversity,^37,38,39,40,41^ (3) self-esteem below average,^42,43^ (4) willingness to participate, (5) ability to provide informed consent, and (6) parental consent for minors. Individuals were excluded if they had insufficient command of the Dutch language and/or psychiatric symptoms due to an organic cause (eTable 1 and eMethods 2 in Supplement 2). Data on basic sociodemographic characteristics were collected on age, sex, level of education, and employment status, and self-ascribed ethnicity and race (ie, Moroccan, Turkish, Surinamese, other) to allow for a basic epidemiological characterization of the sample based on evidence on the basic risk factor epidemiology of mental disorders.

### Randomization and Masking

Participants were randomly assigned (50:50) to the experimental or control condition at the participant level. Block randomization in blocks of 6 was performed by an independent researcher through a computer-generated sequence, with stratification for region of collaborating center or external admission. Assessors administering self-report and interview-based outcome measures were masked to allocation. Breaks in masking were documented and another blinded assessor repeated the assessment.

### Interventions

#### Control Condition: CAU

Participants allocated to the control condition received CAU, which included access to all standard health care and social services that they would have received if they had not participated in the study, except for manualized treatment that explicitly addresses self-esteem as a primary target (eg, Competitive Memory Training [COMET]/Eye Movement Desensitization and Reprocessing [EMDR]^42^) during the intervention period.

#### Experimental Condition: Blended EMI + CAU

The transdiagnostic, blended EMI for improving self-esteem was delivered within a 6-week period in addition to CAU to individuals allocated to the experimental condition. This blended intervention consisted of 3 face-to-face sessions (on-site or online), delivered by trained mental health professionals, who were trained mental health professionals, 3 email contacts, and an EMI administered through a smartphone-based app for adaptive real-time and real-world transfer. The intervention was based on principles of EMIs,^27,28,29,30^ and a guided self-help approach using principles of cognitive behavioral therapy.^44,45^ The EMI translated the training from the face-to-face sessions into individuals’ daily lives based on 3 types of delivery schemes (eMethods 1 and eTable 2 in Supplement 2).

### Measures

Blinded assessors collected outcome data before randomization (baseline), at the end of the 6-week intervention period (postintervention), and at 6-month follow-up (follow-up). The primary outcome was global self-esteem, measured with the Rosenberg Self-Esteem Scale (RSES),^46^ a widely used measure to assess global self-esteem with good reliability and validity.^43,47^ The RSES consists of 10 items rated on a 4-point Likert scale ranging from strongly agree to strongly disagree. The level of global self-esteem, operationalized as the total score of the RSES, was compared between experimental and control condition at postintervention and follow-up. Details on all screening and outcome measures are provided in eMethods 2 in Supplement 2. Secondary outcomes included positive and negative self-esteem,^48^ positive and negative schematic self-beliefs,^49^ emotional well-being,^50^ psychological distress,^51,52^ general psychopathology,^52,53^ clinical symptoms,^54,55^ functioning,^56,57^ and quality of life.^52,58^ Momentary self-esteem,^24,25^ resilience,^59,60,61^ and positive/negative affect^62,63,64^ were measured with the EMA.^64,65,66^ Serious adverse events (SAEs) were monitored throughout the entire study period.

### Statistical Analysis

Statistical analysis was performed blind to random allocation and according to the intention-to-treat principle based on a preregistration and statistical analysis plan published on the open science framework.^67^ The trial was powered to detect an effect size (standardized mean difference) of 0.3 (experimental vs control condition),^68^ with sample size calculation indicating that a sample size of 130 participants (65 per condition) is sufficient to test our hypothesis that levels of self-esteem (primary outcome) are, on average, higher in the experimental than control condition across postintervention and follow-up (power = 0.87; 2-tailed α = .05) using linear mixed modeling. To allow for 12.5% attrition at 6-month follow-up (25% at 24-month follow-up), we aimed to randomize 174 participants at baseline. The current article reports findings from the a priori planned analyses on the primary and secondary outcomes at postintervention and follow-up (as detailed in relation to hypothesis 1 and 2 in the prespecified published statistical analysis plan).^67^ All analyses were conducted in Stata, version 16 (StataCorp).^69^

We fitted a linear mixed model to test the primary hypothesis of the effect of blended EMI plus CAU compared with CAU alone on the primary outcome, with self-esteem (RSES) at postintervention and follow-up as dependent variables. We controlled for baseline self-esteem and the following independent variables in this model: time, condition, and region/center (stratifier). We analyzed all secondary outcomes using linear mixed modeling. We calculated Cohen *d*-type effect sizes and used robust restricted maximum likelihood in mixed models to account for missing outcome data under the assumption that data are missing at random, conditional on the covariates. All *P* values were 2 sided, and a *P* value <.05 was considered statistically significant (eMethods 3 in Supplement 2).

## Results

Of the 425 potential participants initially identified, 223 were assessed for eligibility (Figure). Of these, 174 participants (mean [SD] age, 20.7 [3.1] years; 154 female [89%]; 19 male [11%]) were randomly assigned to a blended EMI plus CAU (85 [49%]) or CAU only (89 [51%]). Primary outcome data were available for 153 participants (87.9%) at postintervention, 140 participants (80.5%) at follow-up, and at least postintervention or follow-up for 159 participants (91.4%).

**Figure.**
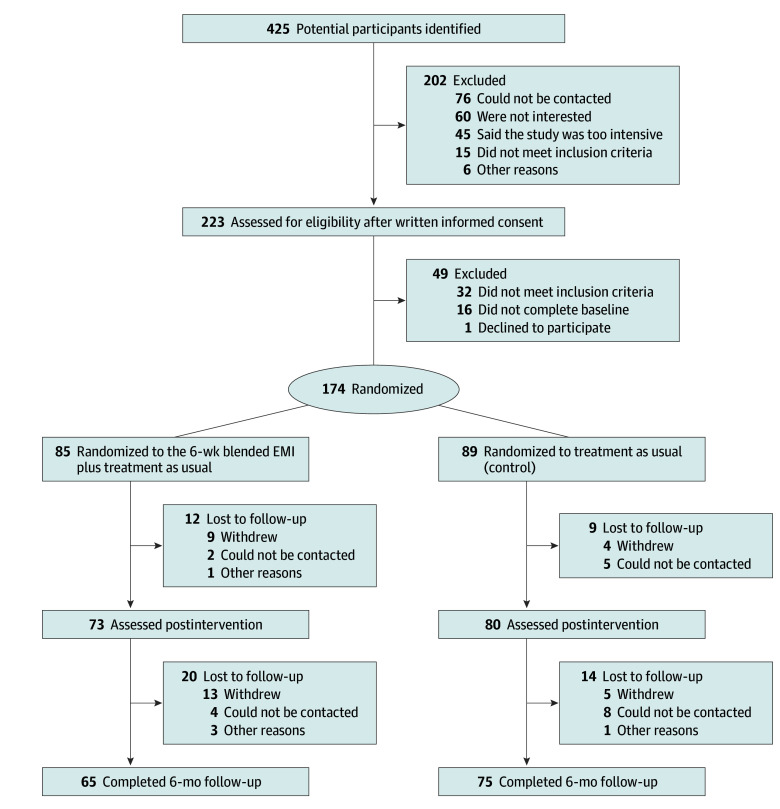
Study Flowchart EMI indicates ecological momentary intervention.

Baseline sample characteristics are shown in Table 1^69,70^ and eTables 3 to 5 in Supplement 2. Participants were largely currently in school, vocational training, or university (122 of 174 [70.1%]). At randomization, 87 individuals were in contact with secondary mental health services in collaborating study centers, and 87 individuals (50.0%) were from the general population (of these, 54 [62.1%] and, hence, 141 [81.0%] of the total sample, received help from secondary mental health services at study entry). Most participants reported exposure to childhood emotional abuse, emotional neglect, verbal bullying, indirect bullying, and/or parental conflict, but, still, a substantial proportion reported exposure to childhood sexual abuse (65 of 174 [37.4%]), physical abuse (32 of 174 [18.4%]), and physical bullying (69 of 173 [39.9%]). Mean (SD) self-esteem score measured with the RSES (18.9 [3.8]) was, on average, well above the inclusion threshold (score <26).

**Table 1.** Baseline Characteristics of the Intention-to-Treat Sample

Characteristic	Total No.	Full sample	Total No.	Experimental condition	Total No.	Control condition
						
Age, mean (SD), y	174	20.67 (3.08)	85	20.86 (3.00)	89	20.49 (3.15)
Sex, No. (%)^a^	174		85		89	
Female		154 (89.0)		73 (86.9)		81 (91.0)
Male		19 (11.0)		11 (13.1)		8 (9.0)
Self-ascribed race and ethnicity, No. (%)	174		85		89	
Migrant/ethnic minority^b^		25 (14.4)		12 (14.1)		13 (14.6)
White majority		149 (85.6)		73 (85.9)		76 (85.4)
Level of education (completed, ongoing), No. (%)^c^	173		85		88	
Low		77 (44.5)		42 (49.4)		35 (39.8)
Middle		75 (43.4)		34 (40.0)		41 (46.6)
High		21 (12.1)		9 (10.6)		12 (13.6)
Employment status, No. (%)	174		85		89	
Employed		22 (12.7)		14 (16.5)		8 (9.0)
Student^d^		122 (70.1)		57 (67.1)		65 (73.0)
Unemployed		30 (17.2)		14 (16.5)		16 (18.0)
Medication, No. (%)		112 (64.4)		52 (61.2)		60 (67.4)
Study center/recruitment route into study, No. (%)	174		85		89	
Noord-Holland		12 (6.9)		6 (7.1)		6 (6.7)
Zuid-Holland		25 (14.4)		11 (12.9)		14 (15.7)
Limburg		50 (28.7)		26 (30.6)		24 (27.0)
General population		87 (50.0)		42 (49.4)		45 (50.6)
Clinical characteristics, mean (SD)						
Childhood trauma						
Emotional abuse, mean (SD), score	174	14.48 (5.05)	85	14.11 (5.02)	89	14.83 (5.09)
Score above inclusion threshold, No. (%)	174	113 (64.9)	85	56 (65.9)	89	57 (64.0)
Physical abuse, mean (SD), score	174	7.16 (3.27)	85	6.81 (3.13)	89	7.48 (3.39)
Score above inclusion threshold, No. (%)	174	32 (18.4)	85	13 (15.3)	89	19 (21.4)
Sexual abuse, mean (SD), score	174	9.24 (6.19)	85	8.19 (4.98)	89	10.24 (7.04)
Score above inclusion threshold, No. (%)	174	65 (37.4)	85	26 (30.6)	89	39 (43.8)
Emotional neglect, mean (SD), score	174	14.55 (4.87)	85	14.47 (4.73)	89	14.62 (5.01)
Score above inclusion threshold, No. (%)	174	92 (52.9)	85	42 (49.4)	89	50 (56.2)
Physical neglect, mean (SD), score	174	8.65 (3.14)	85	8.78 (3.40)	89	8.53 (2.89)
Score above inclusion threshold, No. (%)	174	63 (36.2)	85	30 (35.3)	89	33 (37.1)
RBQ						
Frequency physical bullying, mean (SD), score	173	2.14 (1.28)	84	2.12 (1.21)	89	2.17 (1.34)
Score above inclusion threshold, No. (%)	173	69 (39.9)	84	35/84 (41.7)	89	34 (38.20)
Severity physical bullying, mean (SD), score	173	2.29 (1.34)	84	2.33 (1.37)	89	2.26 (1.32)
Score above inclusion threshold, No. (%)	173	47 (27.2)	84	24/84 (28.6)	89	23 (25.8)
Frequency verbal bullying, mean (SD), score	173	3.48 (1.28)	85	3.45 (1.24)	89	3.52 (1.32)
Score above inclusion threshold, No. (%)	174	142 (81.6)	85	69/85 (81.2)	89	73 (82.0)
Severity verbal bullying, mean (SD), score	174	3.35 (1.25)	85	3.32 (1.26)	89	3.38 (1.25)
Score above inclusion threshold, No. (%)	174	101 (58.1)	85	47/85 (55.3)	89	54 (60.7)
Frequency indirect bullying, mean (SD), score	172	3.58 (1.27)	84	3.50 (1.29)	88	3.65 (1.24)
Score above inclusion threshold, No. (%)	172	142 (82.6)	84	69 (83.14)	88	73 (82.95)
Severity indirect bullying, mean (SD), score	172	3.41 (1.24)	84	3.32 (1.20)	88	3.49 (1.28)
Score above inclusion threshold, No. (%)	172	95 (55.2)	84	46 (54.8)	88	49 (55.7)
CECA parental conflict						
Frequency, mean (SD), score	173	2.17 (1.74)	84	2.11 (1.80)	89	2.22 (1.69)
Score above inclusion threshold, No. (%)	173	88 (50.9)	84	41 (48.8)	89	47 (52.8)
Severity, mean (SD), score	173	1.89 (1.54)	84	1.74 (1.55)	89	2.03 (1.53)
Score above inclusion threshold, No. (%)	173	81 (46.8)	84	34 (40.5)	89	47 (52.8)
Time since parental conflict, mean (SD), y^e^	114	6.29 (5.04)	52	7.10 (5.27)	62	5.61 (4.77)
RSES, mean (SD), score	174	18.94 (3.80)	85	18.52 (3.85)	89	19.35 (3.72)
SERS, mean (SD), score						
Positive self-esteem	173	28.69 (9.05)	85	27.36 (9.46)	88	29.98 (8.50)
Negative self-esteem	174	39.36 (10.02)	85	38.92 (11.10)	89	39.79 (8.93)
BCSS, mean (SD), score						
Positive self	174	5.79 (4.22)	85	5.33 (4.10)	89	6.22 (4.32)
Negative self	174	9.25 (5.19)	85	8.98 (5.06)	89	9.52 (5.32)
Positive other	174	7.09 (4.16)	85	7.06 (4.26)	89	7.12 (4.09)
Negative other	174	5.32 (4.28)	85	5.11 (4.39)	89	5.53 (4.17)
PANAS positive affect, mean (SD)	174	22.12 (5.97)	85	21.71 (5.37)	89	22.52 (6.50)
PANAS negative affect, mean (SD)	174	22.94 (9.15)	85	22.04 (9.11)	89	23.80 (9.15)
K10, mean (SD), total score	174	27.55 (7.48)	85	28.11 (7.69)	89	27.02 (7.29)
SCL-90-R, mean (SD), total score	173	229.00 (58.88)	84	221.51 (60.61)	89	236.07 (56.64)
BPRS, mean (SD), total score	174	44.71 (7.91)	85	44.22 (7.58)	89	45.187 (8.22)
WHOQOL-BREF, mean (SD), score						
Physical domain	172	21.58 (4.01)	84	21.68 (4.00)	88	21.49 (4.03)
Psychological domain	173	14.21 (3.52)	84	14.18 (3.68)	89	14.24 (3.39)
Social domain	173	9.05 (2.57)	84	8.85 (2.74)	89	9.25 (2.38)
Environmental domain, mean (SD), score	173	27.98 (4.55)	84	28.01 (4.69)	89	27.96 (4.44)
SOFAS	174	65.30 (11.25)	85	66.11 (10.74)	89	64.54 (11.72)
GAF	174	63.71 (10.10)	85	64.31 (9.45)	89	63.13 (10.72)

Abbreviations: BCSS, Brief Core Schema Scale; BPRS, Brief Psychiatric Rating Scale; GAF, Global Assessment of Functioning; K10, Kessler Psychological Distress Scale; PANAS, Positive and Negative Affect Scale; RBQ, Retrospective Bullying Questionnaire; RSES, Rosenberg Self-esteem Scale; SCL-90-R, Symptom Checklist 90-R; SERS, Self-Esteem Rating Scale; SOFAS, Social and Occupational Functioning Assessment Scale; WHOQOL-BREF, World Health Organization Quality of Life Instrument-Brief.

^a^ There were too few participants with nonbinary gender to provide numbers without compromising identifiability.

^b^ Includes Moroccan, Turkish, Surinamese, and other.

^c^ Education degree adapted from the Dutch Standard Classification of Education.^69^

^d^ The student category student includes primary education, lower vocational education, secondary general education, secondary vocational education, secondary general education, higher general and vocational education, and university.

^e^ Time since parental conflict reported for those exposed to any conflict, including those below inclusion threshold.

Attendance of intervention sessions was generally high, with 77 participants (90.6%) attending at least 1 session (Table 2 and eTable 6 in Supplement 2).^44,45^ Session attendance ranged from 85.9% (session 3) to 89.4% (session 2) to 90.6% (session 1). Fidelity to intervention protocol was overall rated as high by therapists and independent researchers, with participants completing, on average, approximately 13 EMI tasks per week. Further, moderate to high user experience, satisfaction, and acceptance ratings were reported for the novel transdiagnostic intervention. Overall, 1 SAE (ie, 1 inpatient admission) deemed unrelated to the blended EMI was reported in the experimental condition, and 2 SAEs (2 inpatient admissions) were reported in the control condition.

**Table 2.** Key Components of the Transdiagnostic Ecological Momentary Intervention (EMI)^a^

Training session	Transdiagnostic ecological momentary intervention					
	Week 1. Face-to-face session 1	Week 2. Email contact 1	Week 3. Face-to-face session 2	Week 4. Email contact 2	Week 5. Face-to-face session 3	Week 6. Email contact 3
Enhancing EMI tasks	Formulating a new positive core belief + positive datalog (enter daily successes)	Personal positive qualities (integrated in positive datalog) + tips to identify more positive qualities + one-minute exercise (listing [previously identified] positive qualities)	Overview old behavioral patterns + development of new behavior patterns	Expanding the positive datalog with successes arising from new behavioral patterns	Strategies to deal with criticism + a critical look at criticism + cost-benefit analysis of perfectionism + the minimum program (practicing to perform less than perfect)	Writing a positive story about yourself + maintenance plan (after the intervention)
Consolidating EMI tasks	Positive datalog + tips to add more successes in the positive datalog + rating credibility of the new core belief	Positive datalog + tips positive datalog + one-minute exercise + rating credibility of the new core belief	Positive datalog + 1-min exercise + rating credibility of the new core belief	Positive datalog + 1-min exercise + expanding new behavior patterns + rating credibility of the new core belief	Positive datalog + 1-min exercise + a critical look at criticism + rating credibility of the new core belief	Positive datalog + 1-min exercise + rating credibility of the new core belief
Interactive EMI tasks	Positive datalog (adding successes) or positive datalog (viewing previously identified successes)	NA	Positive datalog (adding successes and/or positive qualities) or positive datalog (viewing previously identified successes and qualities)	NA	A critical look at criticism	NA

Abbreviation: NA, not applicable.

^a^ De Neef^44^ and Postma et al.^45^

Table 3 shows findings for all primary and secondary outcomes. We found evidence on the efficacy of the blended EMI on improved global self-esteem measured with the RSES as primary outcome, which was, on average, higher in the experimental than control condition across postintervention and follow-up (B = 2.32; 95% CI, 1.14-3.50; *P* < .001; Cohen *d*-type effect size = 0.54). When inspecting findings further, at each time point separately, higher levels of global self-esteem in the experimental than control condition were evident at postintervention (B = 2.83; 95% CI, 1.46-4.20; *P* < .001; Cohen *d*-type effect size = 0.66) and follow-up (B = 1.81; 95% CI, 0.38-3.23; *P* = .01; Cohen *d*-type effect size = 0.42), with differences being in the moderate effect size range (eFigure and eMethods 4 in Supplement 2).

**Table 3.** Primary and Secondary Outcomes and Secondary Mechanisms at Postintervention and 6-Month Follow-Up^a^

Outcome	Experimental condition		Control condition		Difference between conditions (at time), adjusted B (95% CI)	*P* value	Cohen *d*-type effect size (95% CI)
	Mean (SE)	No.	Mean (SE)	No.			
Primary outcome							
Global self-esteem (RSES)							
Main effect of condition	NA	NA	NA	NA	2.32 (1.14 to 3.50)	<.001	0.54 (0.26 to 0.82)
Time							
Postintervention	24.04 (0.62)	73	21.89 (0.51)	80	2.83 (1.46 to 4.20)	<.001	0.66 (0.33 to 0.98)
Follow-up	24.43 (0.71)	65	23.31 (0.68)	75	1.81 (0.38 to 3.23)	.01	0.42 (0.09 to 0.75)
Secondary outcomes							
Positive self-esteem (SERS)							
Main effect of condition	NA	NA	NA	NA	3.85 (1.83 to 5.88)	<.001	0.53 (0.24 to 0.81)
Time							
Postintervention	34.54 (1.28)	71	31.90 (0.90)	81	4.11 (1.75 to 6.47)	.001	0.56 (0.23 to 0.89)
Follow-up	34.68 (1.27)	62	31.79 (1.06)	72	3.59 (1.11 to 6.08)	.005	0.49 (0.15 to 0.84)
Negative self-esteem (SERS)							
Main effect of condition	NA	NA	NA	NA	−3.78 (−6.59 to −0.98)	.008	−0.38 (−0.67 to −0.10)
Time							
Postintervention	30.41 (1.50)	71	35.31 (1.28)	81	−4.41 (−7.57 to −1.25)	.006	−0.45 (−0.77 to −0.12)
Follow-up	27.35 (1.66)	62	32.08 (1.51)	72	−3.16 (−6.48 to 0.16)	.06	−0.32 (−0.66 to 0.02)
Negative schematic beliefs of self (BCSS)							
Main effect of condition	NA	NA	NA	NA	−1.71 (−2.93 to −0.48)	.006	−0.39 (−0.68 to −0.11)
Time							
Postintervention	5.83 (0.61)	71	7.96 (0.66)	81	−1.99 (−3.37 to −0.60)	.005	−0.46 (−0.78 to −0.13)
Follow-up	4.32 (0.61)	62	6.29 (0.65)	72	−1.43 (−2.89 to 0.03)	.06	−0.33 (−0.67 to 0.01)
Positive schematic beliefs of self (BCSS)							
Main effect of condition	NA	NA	NA	NA	1.58 (0.41 to 2.75)	.008	0.38 (0.09 to 0.66)
Time							
Postintervention	8.25 (0.60)	71	6.93 (0.55)	81	1.98 (0.63 to 3.33)	.004	0.47 (0.15 to 0.80)
Follow-up	9.00 (0.65)	62	8.06 (0.69)	72	1.18 (−0.24 to 2.60)	.10	0.28 (−0.06 to 0.62)
Negative affect (PANAS)							
Main effect of condition	NA	NA	NA	NA	−1.34 (−3.54 to 0.86)	.23	−0.17 (−0.46 to 0.11)
Time							
Postintervention	19.87 (1.13)	70	20.54 (0.96)	79	−0.27 (−2.75 to 2.21)	.83	−0.04 (−0.36 to 0.29)
Follow-up	16.05 (0.93)	62	19.72 (0.98)	72	−2.41 (−5.00 to 0.18)	.07	−0.31 (−0.65 to 0.02)
Positive affect (PANAS)							
Main effect of condition	NA	NA	NA	NA	0.52 (−1.21 to 2.26)	.55	0.08 (−0.19 to 0.35)
Time							
Postintervention	23.29 (0.93)	70	22.44 (0.74)	79	1.10 (−1.00 to 3.19)	.30	0.17 (−0.15 to 0.49)
Follow-up	21.92 (0.93)	62	22.31 (0.73)	72	−0.05 (−2.25 to 2.16)	.97	−0.01 (−0.35 to 0.33)
General psychopathology (SCL-90-R)							
Main effect of condition	NA	NA	NA	NA	−17.62 (−33.03 to −2.21)	.03	−0.34 (−0.64 to −0.04)
Time							
Postintervention	186.62 (7.91)	71	212.74 (7.21)	80	−17.70 (−34.42 to −0.98)	.04	−0.34 (−0.66 to −0.02)
Follow-up	172.87 (7.70)	62	206.60 (8.12)	72	−17.54 (−34.93 to −0.15)	.048	−0.34 (−0.67 to 0)
Psychological distress (K10)							
Main effect of condition	NA	NA	NA	NA	1.69 (−0.34 to 3.72)	.10	0.23 (−0.05 to 0.52)
Time							
Postintervention	32.07 (0.96)	71	30.16 (0.93)	81	1.27 (−1.03 to 3.56)	.28	0.18 (−0.14 to 0.50)
Follow-up	34.02 (1.11)	61	30.69 (1.05)	72	2.10 (−0.32 to 4.53)	.09	0.29 (−0.05 to 0.63)
Clinical symptoms (BPRS)							
Main effect of condition	NA	NA	NA	NA	−1.01 (−3.06 to 1.03)	.33	−0.14 (−0.43 to 0.14)
Time							
Postintervention	41.21 (0.89)	70	43.84 (1.14)	77	−1.68 (−4.00 to 0.65)	.16	−0.23 (−0.56 to 0.09)
Follow-up	40.24 (0.93)	59	41.58 (1.06)	67	−0.35 (−2.83 to 2.13)	.78	−0.05 (−0.40 to 0.30)
Quality of life (WHOQOL-BREF; Physical domain)							
Main effect of condition	NA	NA	NA	NA	1.06 (−0.11 to 2.23)	.08	0.25 (−0.03 to 0.53)
Time							
Postintervention	23.86 (0.57)	69	22.54 (0.51)	80	1.10 (−0.25 to 2.46)	.11	0.26 (−0.06 to 0.59)
Follow-up	43.05 (0.59)	62	22.71 (0.58)	72	1.02 (−0.40 to 2.43)	.16	0.24 (−0.10 to 0.58)
Quality of life (WHOQOL-BREF; Psychological domain)							
Main effect of condition	NA	NA	NA	NA	1.16 (0.18 to 2.13)	.02	0.33 (0.05 to 0.61)
Time							
Postintervention	17.14 (0.51)	69	15.46 (0.49)	80	1.54 (0.41 to 2.66)	.007	0.44 (0.11 to 0.76)
Follow-up	17.26 (0.61)	62	16.31 (0.55)	72	0.77 (−0.40 to 1.95)	.20	0.22 (−0.12 to 0.56)
Quality of life (WHOQOL-BREF; Social domain)							
Main effect of condition	NA	NA	NA	NA	0.20 (−0.43 to 0.83)	.53	0.09 (−0.19 to 0.37)
Time							
Postintervention	9.60 (0.36)	70	9.30 (0.28)	80	0.45 (−0.28 to 1.19)	.22	0.20 (−0.12 to 0.52)
Follow-up	9.98 (0.34)	62	9.83 (0.27)	71	−0.05 (−0.82 to 0.72)	.89	−0.02 (−0.36 to 0.31)
Quality of life (WHOQOL-BREF; Environmental domain)							
Main effect of condition	NA	NA	NA	NA	1.50 (0.35 to 2.65)	.01	0.37 (0.08 to 0.65)
Time							
Postintervention	29.65 (0.60)	69	28.19 (0.60)	80	1.41 (0.10 to 2.72)	.04	0.35 (0.02 to 0.67)
Follow-up	30.27 (0.66)	62	28.36 (0.58)	72	1.59 (0.23 to 2.96)	.02	0.39 (0.05 to 0.73)
Functioning (SOFAS)							
Main effect of condition	NA	NA	NA	NA	−0.80 (−3.43 to 1.82)	.55	−0.08 (−0.35 to 0.19)
Time							
Postintervention	68.61 (1.27)	70	67.25 (1.26)	77	0.56 (−2.62 to 3.74)	.73	0.06 (−0.27 to 0.38)
Follow-up	68.78 (1.36)	60	69.28 (1.48)	68	−2.17 (−5.57 to 1.24)	.21	−0.22 (−0.57 to 0.13)
Functioning (GAF)							
Main effect of condition	NA	NA	NA	NA	0.46 (−2.18 to 3.10)	.73	0.05 (−0.23 to 0.32)
Time							
Postintervention	67.86 (1.24)	69	65.97 (1.22)	76	1.17 (−1.99 to 4.33)	.47	0.12 (−0.21 to 0.45)
Follow-up	68.02 (1.22)	60	66.82 (1.53)	68	−0.25 (−3.60 to 3.11)	.89	−0.03 (−0.37 to 0.32)

Abbreviations: BCSS, Brief Core Schema Scale; BPRS, Brief Psychiatric Rating Scale; GAF, Global Assessment of Functioning; K10, Kessler Psychological Distress Scale; NA, not applicable; PANAS, Positive and Negative Affect Scale; RSES, Rosenberg Self-esteem Scale; SCL-90-R, Symptom Checklist 90-R; SERS, Self-Esteem Rating Scale; SOFAS, Social and Occupational Functioning Assessment Scale; WHOQOL-BREF, World Health Organization Quality of Life Instrument-Brief.

^a^ Adjusted for centered baseline values, region, and group status.

Small to moderate effect sizes signaled beneficial effects of the blended EMI on secondary outcomes, with, on average, lower levels of negative self-esteem (B = −3.78; 95% CI, −6.59 to −0.98; *P* = .008; Cohen *d*-type effect size = −0.38), negative schematic self-beliefs (B = −1.71; 95% CI, −2.93 to −0.48; *P* = .006; Cohen *d*-type effect size = −0.39) as well as higher levels of positive self-esteem (B = 3.85; 95% CI, 1.83-5.88; *P* < .001; Cohen *d*-type effect size = 0.53) and positive schematic self-beliefs (B = 1.58; 95% CI, 0.41-2.75; *P* = .008; Cohen *d*-type effect size = 0.38) in the experimental than control condition across postintervention and follow-up.

We also observed, on average, lower levels of self-reported general psychopathology (B = −17.62; 95% CI, −33.03 to −2.21; *P* = .03; Cohen *d*-type effect size = −0.34) in the experimental than control condition across postintervention and follow-up. Small to moderate effect sizes signaled beneficial effects of the blended EMI on quality of life, with higher levels of quality of life, on average, in the experimental than control condition across postintervention and follow-up for both the psychological (B = 1.16; 95% CI, 0.18-2.13; *P* = .02; Cohen *d*-type effect size = 0.33) and environmental domain (B = 1.50; 95% CI, 0.35-2.65; *P* = .01; Cohen *d*-type effect size = 0.37). There was no evidence on improvements in self-reported psychological distress, emotional well-being, clinical symptoms, and functioning.

Small effect sizes suggestive of momentary beneficial effects of the blended EMI indicated, on average, higher levels of momentary self-esteem (B = 0.29; 95% CI, 0.01-0.57; *P* = .04; Cohen *d*-type effect size = 0.24) and momentary positive affect (B = 0.23; 95% CI, 0.01-0.45; *P* = .04; Cohen *d*-type effect size = 0.20) as well as lower levels of momentary negative affect (B = −0.33; 95% CI, −0.59 to −0.06; *P* = .01; Cohen *d*-type effect size = −0.27) in the experimental than control condition across postintervention and follow-up (Table 4).

**Table 4.** Secondary Outcomes Measured With Ecological Momentary Assessment at Postintervention and 6-Month Follow-Up

Outcome	Postintervention			Follow-up			Adjusted B (95% CI)	*P* value	Cohen *d*-type effect size (95% CI)
	Adjusted B (95% CI)	*P* value	Cohen *d*-type effect size (95% CI)	Adjusted B (95% CI)	*P* value	Cohen *d*-type effect size (95% CI)			
**Momentary self-esteem**									
Condition									
Experimental condition	3.32 (2.71 to 3.94)	<.001	NA	3.86 (3.23 to 4.49)	<.001	NA	NA	NA	NA
Control condition	3.07 (2.46 to 3.69)	<.001	NA	3.53 (2.90 to 4.17)	<.001	NA	NA	NA	NA
Experimental vs control condition	0.25 (−0.04 to 0.54)	.09	0.21 (−0.03 to 0.46)	0.33 (0.00 to 0.65)	.047	0.28 (0.00 to 0.55)	NA	NA	NA
Time × condition	NA	NA	NA	NA	NA	NA	0.29 (0.01 to 0.57)	.04	0.24 (0.01 to 0.48)
**Negative affect** ^a^									
Condition									
Experimental condition	1.81 (1.19 to 2.43)	<.001	NA	1.60 (1.00 to 2.20)	<.001	NA	NA	NA	NA
Control condition	2.15 (1.51 to 2.78)	<.001	NA	1.91 (1.29 to 2.53)	<.001	NA	NA	NA	NA
Experimental vs control condition	−0.34 (−0.64 to −0.03)	.03	−0.28 (−0.54 to −0.03)	−0.31 (−0.60 to −0.03)	.03	−0.27 (−0.50 to −0.03)	NA	NA	NA
Time × condition	NA	NA	NA	NA	NA	NA	−0.33 (−0.59 to −0.03)	.01	−0.27 (−0.49 to −0.06)
**Positive affect**									
Condition									
Experimental condition	3.33 (2.79 to 3.87)	<.001	NA	3.67 (3.12 to 4.20)	<.001	NA	NA	NA	NA
Control condition	3.10 (2.57 to 3.64)	<.001	NA	3.43 (2.89 to 3.97)	<.001	NA	NA	NA	NA
Experimental vs control condition	0.23 (−0.02 to 0.48)	.08	0.20 (−0.02 to 0.42)	0.23 (−0.02 to 0.49)	.07	0.21 (−0.02 to 0.43)	NA	NA	NA
Time × condition	NA	NA	NA	NA	NA	NA	0.23 (0.01 to 0.45)	.04	0.20 (0.01 to 0.40)
**Momentary resilience: positive affective recovery**									
Condition									
Experimental condition	−0.31 (−0.56 to −0.06)	.01	NA	−0.40 (−0.65 to −0.15)	.002	NA	NA	NA	NA
Control condition	−0.16 (−0.38 to 0.06)	.16	NA	−0.11 (−0.36 to 0.15)	.42	NA	NA	NA	NA
Experimental vs control condition	−0.15 (−0.49 to 0.18)	.37	−0.12 (−0.38 to 0.14)	−0.29 (−0.65 to 0.07)	.11	−0.23 (−0.48 to 0.03)	NA	NA	NA
Time × condition	NA	NA	NA	NA	NA	NA	−0.22 (−0.47 to 0.02)	.07	−0.17 (−0.35 to 0.01)
**Momentary resilience: negative affective recovery**									
Condition									
Experimental condition	0.29 (0.04 to 0.53)	.02	NA	0.35 (0.10 to 0.60)	.005	NA	NA	NA	NA
Control condition	0.16 (−0.05 to −0.38)	.14	NA	0.16 (−0.10 to 0.41)	.23	NA	NA	NA	NA
Experimental vs control condition	0.12 (−0.20 to 0.45)	.46	0.11 (−0.15 to 0.33)	0.19 (−0.16 to 0.55)	.28	0.09 (−0.10 to 0.37)	NA	NA	NA
Time × condition	NA	NA	NA	NA	NA	NA	0.16 (−0.08 to 0.40)	.20	0.14 (−0.06 to 0.28)

Abbreviation: NA, not applicable.

^a^ This model did not converge with the specification of the correlation between residual errors being time ordered. The results for the other models that did converge (for self-esteem and positive affect) were similar after exclusion of time-ordered correlation between residual errors. Therefore, we removed this specification for this model and assume that this did not impact the results.

## Discussion

### Main Findings

This definitive RCT moved beyond previous research by establishing the efficacy of a novel, cutting-edge, transdiagnostic blended EMI for improving self-esteem in the daily lives of youth with prior exposure to childhood adversity. We found evidence on the efficacy of the blended EMI on improved global self-esteem across postintervention and follow-up as a primary outcome. Effect sizes were also suggestive of improvements in positive and negative self-esteem, schematic self-beliefs, and momentary self-esteem in daily life as secondary outcomes. Although effect sizes were suggestive of beneficial effects on self-reported general psychopathology and quality of life, no improvements were evident in observer-rated symptoms and functioning. Adherence, fidelity, and acceptance were moderate to high, with no evidence of the novel EMI being harmful.

### Strengths and Limitations

#### Methodological Considerations

This study was conducted in line with CONSORT reporting guidelines^34,35^ and allowed us to benefit from the methodological rigor of a definitive RCT in eliminating important sources of bias and confounding. Even though secondary outcomes were interpreted based on effect size (rather than statistical significance) and type I error control is not obligatory in the reporting of secondary outcomes for RCTs,^34^ the beneficial effects on our (distinct but intercorrelated) secondary outcomes would have held for positive self-esteem (at *P* < .001) if this finding was conservatively adjusted for type I error. However, the trial did neither include an active control condition nor blinding of participants to random allocation to further minimize risk of bias (eg, teaching to the test of participants). In line with the broad scope of selective prevention in public mental health provision, the comparator condition was selected to be CAU, broadly defined, including access to all standard health care and social services. Given that systematic delivery of evidence-based prevention strategies in the broader public mental health care domain is still limited, a key question from a public health perspective remains whether our novel blended EMI confers superiority beyond that of CAU. Hence, design and comparator in this trial (ie, CAU, broadly defined) reflected an optimal choice for ensuring high external and ecological validity,^71^ as it mirrors the naturalistic service landscape and practice to which beneficial effects ought to generalize, including to newly emerging digital mental health services, as reflected in offering the novel EMI to youth identified not only via conventional secondary mental health services but also via digital outreach (accounted for as stratifier in this trial). Future research now needs to clarify why and how the novel EMI works in our qualitative work and subsequent trials using an active control condition and/or different dosages of the EMI (eg, face-to-face sessions, EMI components) as comparator.

Concerns have been raised that digital interventions may create new rather than lower barriers to care.^72^ However, in designing the blended EMI, we paid careful attention to ensure acceptance, adherence, and accessibility, which were found to be moderate to high. Further, even though the COVID-19 pandemic may have impacted youth due to infection control measures, this impact is considered to be balanced across conditions in RCTs. Most participants were female, the blended EMI is currently only available in the Dutch language, and no improvements were evident in observer-rated symptoms/functioning. Hence, future work needs to improve reach to male and vulnerable populations (eg, by broadening adversity criteria, translation to other languages, gamification). It further needs to improve and better capture proximal and long-term effects on observer-rated symptoms and functioning by enhancing EMI content/techniques (eg, through closer linkage with symptoms/functioning), optimizing EMI adaptive/interactive components based on cutting-edge methodology (ie, machine learning), and allowing for longer periods of follow-up^73^ (eAppendix 1 in Supplement 2).

#### Comparison With Previous Research

Several experience sampling studies have investigated the role of momentary self-esteem in pathways to adult mental ill health.^17,23,24,25,26^ Current estimates of attributable risks suggest that interventions targeted at eliminating childhood adversity as a risk factor in the population can prevent a substantial proportion of the incidence of adult mental disorders, and, thereby, have a sizable public health impact.^10,74^ Although primary prevention remains the key goal for public health and policy, preventing childhood adversity from exerting its detrimental effects reflects a tangible selective strategy for preventing mental disorders in later life of those exposed and improving well-being, resilience, and outcome in youth already in contact with services.^12,13,63^ Given its role in pathways from childhood adversity to various adult mental disorders, self-esteem reflects a primary transdiagnostic target for such strategies,^14,15^ both as a mechanism and/or early symptom of mental disorder. However, ecological translation to individuals’ daily lives continues to be a major challenge. In addressing this challenge and targeting momentary self-esteem in the daily lives of youth exposed to childhood adversity, the blended EMI that we have evaluated here substantially moves beyond previous research. Notably, this blended EMI yielded significant improvements in the primary outcome of global self-esteem of moderate effect size, corroborated by effect sizes suggestive of beneficial effects in secondary outcomes on positive and negative self-esteem, schematic self-beliefs, and, importantly, momentary self-esteem in daily life. Although these effect sizes were similar to, and in part even larger than, those reported in meta-analyses of other interventions specifically targeting self-esteem,^68,75^ this blended EMI involved a substantially lower number of sessions, yielded sustained effects at follow-up, and, at its core, was geared toward ecological translation to individuals’ daily lives (also reflected in effect sizes suggestive of improvements in momentary self-esteem). Although no improvements were evident in observer-rated symptoms and functioning, effect sizes signaled beneficial effects for self-reported general psychopathology and quality of life, partially supportive of the ecological interventionist causal model approach adopted here,^30,31^ which further needs to clarify the role of self-esteem as transdiagnostic target mechanism and/or outcome, and use improved clinical measures that are genuinely transdiagnostic in nature (eAppendix 2 in Supplement 2).

Overall, effect sizes for primary and secondary outcomes tended to be larger at postintervention than at follow-up. One obvious reason for this may be that the intervention, including the app-based EMI, was discontinued at postintervention. Hence, the beneficial effects may be sustained by allowing for continued access to the app-based EMI at very limited cost. This should be tested in a naturalistic implementation trial informed by secondary analyses of the current trial on long-term effects at 18- and 24-month follow-up, which will allow for a formal test of the ecological interventionist causal model approach as well as an evaluation of cost-effectiveness and cost-utility of the blended EMI.

## Conclusions

Transdiagnostic mechanisms implicated in the socioenvironmental origins of mental disorders are important targets for prevention and intervention. To our knowledge, this study was the first RCT that sought to test a transdiagnostic, blended EMI directly targeting self-esteem in daily life in youth with prior exposure to childhood adversity as priority target population. The trial results provided evidence for the efficacy of the novel EMI on improving global self-esteem as a primary outcome of moderate effect size sustained at postintervention and follow-up, corroborated by signals of beneficial effects on secondary outcomes. Taken together, these findings are highly relevant for furthering our understanding of self-esteem as a transdiagnostic mechanism, while, at the same time, contributing to the growing knowledge of EMI development and implementation. Future work needs to demonstrate the sustained effects of the novel EMI and adopt appropriate strategies for implementation in routine public and/or digital mental health services.

## Notes

Supplement 1.
Trial Protocol and Statistical Analysis Plan.

Supplement 2. **eTable 1.** Eligibility Criteria **eMethods 1.** SELFIE Intervention Description **eTable 2.** Overview of SELFIE Intervention Procedure **eMethods 2.** Measures **eMethods 3.** Statistical Analysis **eTable 3.** Diagnoses at Baseline **eTable 4.** Correlations Among Primary and Secondary Outcomes at Baseline **eTable 5.** Percentiles and Range of Baseline Characteristics of the Intention-to-Treat Sample **eTable 6.** Compliance With, Fidelity to, and Acceptance of the SELFIE Intervention **eMethods 4.** Sensitivity Analysis on Primary Outcome at Postintervention and Follow-Up Using Multiple Imputation **eFigure.** Response Trajectories in the Primary Outcome (RSES Self-Esteem) **eAppendix 1.** SELFIE in the Context of Previous and Future EMI Research **eAppendix 2.** Targeting Self-Esteem in Ecological Interventionist Causal Models and Transdiagnostic Intervention Research
eReferences

Supplement 3.
Data Sharing Statement.
