# Supplementary material for: Transdiagnostic Ecological Momentary Intervention for Improving Self-Esteem in Youth Exposed to Childhood Adversity: The SELFIE Randomized Clinical Trial
Source: JAMA Psychiatry. 2023 Nov 29;81(3):227–39. doi: 10.1001/jamapsychiatry.2023.4590 (PMC10687716; doi:10.1001/jamapsychiatry.2023.4590)
Supplement: Supplement 1. — Trial Protocol and Statistical Analysis Plan. [file jamapsychiatry-e234590-s001.pdf]

The effects of a transdiagnostic ecological momentary intervention for improving self-esteem (SELFIE) in youth exposed to childhood adversity: data analysis plan for a multi-center randomized controlled trial

Study Information

Hypotheses

(Preliminary remarks)
Sections of this pre-registration are taken from the SELFIE study protocol (please refer to Daemen, Postma (1) for further details). The present preregistration focuses on the main hypotheses on primary and secondary outcomes at 6-month follow-up that will be tested and reported in the first manuscript. In addition, we will report the statistical analysis plan for hypotheses relating to outcomes at 18-month and 24-month follow-up, which will be reported in a subsequent manuscript. The statistical analysis plan for hypotheses relating to hypotheses on cost effectiveness and cost utility will be described in a subsequent preregistration report.

Design Plan

Study type

Experiment - A researcher randomly assigns treatments to study subjects, this includes field or lab experiments. This is also known as an intervention experiment and includes randomized controlled trials.

Blinding

Personnel who interact directly with the study subjects (either human or non-human subjects) will not be aware of the assigned treatments. (Commonly known as "double blind")

Is there any additional blinding in this study?

The assessors are blind to the allocation of participants when assessing outcomes at post-intervention and follow-up. The design of this study is single blinded, because SELFIE therapists and participants cannot be masked towards the allocation of participants to the experimental or control condition. Any data specific to the intervention condition (e.g., on treatment fidelity) will be stored in a separate database. Any breaks in masking will be documented in the trial master file and another assessor will be allocated to complete the next set of assessments where possible.

Study design

In a two-arm parallel-group, assessor-blind multicenter randomized controlled trial, individuals aged between 12 and 26 years with prior exposure to childhood adversity and low self-esteem will be randomly allocated to SELFIE in addition to TAU as the experimental condition or a control condition of TAU only, which includes (access to) standard health care and social services. Participants will be recruited from mental health services in Noord-Holland, Zuid-Holland, and Limburg (the Netherlands) and from the general population (e.g., via social media). Outcomes will be measured at baseline (i.e., before randomization), post-intervention (i.e., after the 6- week intervention period), and 6-month, 18-month, and 24-month follow-up (i.e., 6, 18 and 24 months after completing the intervention period) by blind assessors.

No files selected

Randomization

Each participant will be randomized using blocks of six participants, stratified by region of collaborating centre or as external admission, at a 50:50 ratio to the experimental or control condition after completing the baseline assessment. Randomization will be conducted through a computer-generated sequence, stratified by region of a collaborating center or as external admission. The assessors will be blind to the allocation of subjects when assessing participants at post-intervention, 6-month, 18-month, and 24-month follow-up. After random allocation to the experimental condition, the names and contact details of the participants will be passed on to the SELFIE-therapist providing the SELFIE intervention. This will be done through an independent researcher. This researcher will inform the assessors when assessments at post-intervention and follow-up need to take place for each individual participant.

Sampling Plan

Existing Data

Registration prior to accessing the data

Explanation of existing data

As of the date of submission, recruitment has been completed but data collection is still ongoing. This means that there are already some existing data, but data will only be accessed by MD, MP, AS and UR once outcomes of the last participant will have been assessed at 6-month follow-up, the data analysis plan will have been fully preregistered and published, data checking and cleaning will have been completed, and the database will have been locked.

Data collection procedures

Timeline
Recruitment started in December 2018, the first enrollment was in January 2019, recruitment was completed in June 2021, and outcome assessment will continue until August 2023.

Experience Sampling Methodology (ESM) data collection procedure (required)

Study duration (number of days)
There are five independent periods of six ESM days throughout the study: at baseline, post-intervention, and at 6-month, 18-month, and 24-month follow-up. Each ESM period will include eight assessments per day, scheduled at random within set blocks of time, over a period of six consecutive days at baseline, post-intervention and at 6-month, 18-month, and 24-month follow-up.

Contributors

Ulrich Reininghaus, Maud Daemen, Anita Schick, Mary Rose Postma, Nele Volbragt, Ramon Lindauer, Iris Hoes - van der Meulen, Dorien Nieman, Philippe Delespaul, Josefien Breedvelt, Mark van der Gaag, Wolfgang Viechtbauer, David van den Berg, Claudi Bockting, and Therese van Amelsvoort

Description

For a detailed description of the study, please see our published study protocol (1). This preregistration includes the Statistical Analysis Plan (see sections 21-25) for this multicenter randomized controlled trial, which must be read and considered in conjunction with the published study protocol (1). Targeting low self-esteem in youth exposed to childhood adversity is a promising strategy for preventing adult mental disorder, but psychological help remains difficult to access and accept for youth, calling for novel, youth-friendly approaches. Mobile Health (mHealth) and, most prominently, ecological momentary interventions (EMIs) provide a unique opportunity to deliver youth-friendly, personalized, real-time, guided self-help interventions. The aim of the SELFIE trial is to investigate the efficacy of a novel, accessible, transdiagnostic ecological momentary intervention for improving self-esteem ('SELFIE') in youth aged 12-26 years with prior exposure to childhood adversity. (Preliminary remarks) Sections of this pre-registration are taken from the SELFIE study protocol (please refer to Daemen, Postma (1) for further details). The present preregistration focuses on the main hypotheses on primary and secondary outcomes at 6-month follow-up that will be tested and reported in the first manuscript. In addition, we will report the statistical analysis plan for hypotheses relating to outcomes at 18-month and 24-month follow-up, which will be reported in a subsequent manuscript. The statistical analysis plan for hypotheses relating to hypotheses on cost effectiveness and cost utility will be described in a subsequent preregistration report.
Show less

Registration type
OSF Preregistration

Date registered
August 10, 2022

Date created
August 10, 2022

Associated project
osf.io/zmfdn

Internet Archive link
https://archive.org/details/osf-registrations-8e4bc-v1

Category
Project

Registration DOI
https://doi.org/10.17605/OSF.IO/8E4BC

Subjects
Social and Behavioral Sciences
Life Sciences
Medicine and Health Sciences

License
CC-By Attribution 4.0 International

Tags

This website relies on cookies to help provide a better user experience. By clicking Accept or continuing to use the site, you agree. For more information, see our Privacy Policy and information on cookie use. Accept

time, over a period of six consecutive days at baseline, post-intervention and at 6-month, 18-month, and 24-month follow-up (2, 3).

Total number and type of items (open-ended or closed), estimated questionnaire duration
Full details on the ESM items used in the SELFIE study are reported by Daemen, Postma (1). The ESM questionnaire consists of 24 items. ESM items are closed and rated on either a 7-point Likert scale (e.g., mood or momentary self-esteem) or with multiple-choice answer options (e.g., activity). The ESM questionnaire requires approximately 2 minutes to complete, which amounts to approximately 16 minutes per day, and a total assessment time of 96 minutes per ESM week.

Time-out specifications
When participants receive a notification, the questionnaire remains available for 15 minutes. After this time window has passed, the questionnaire disappears and can no longer be filled in. In case participants stop filling in the questionnaire after they had initiated it, the questionnaire disappeared if they did not continue within 15 minutes.

ESM instructions
In line with previous research, an extensive briefing on ESM occurred at baseline, post-intervention and 6-month, 18-month, and 24-month follow-up.

Hardware and software used
Participants will complete self-report questionnaires using a smartphone-based app (i.e., the PsyMate® app). Following the protocol from previous studies, the PsyMate app was also used for ESM data collection.

Incentives and increasing participant engagement
Participants aged 16 years or older will be financially compensated for their time, and travel expenses will be fully reimbursed. To minimize loss to follow-up, researchers maintain contact with participants on a regular basis. Also, participants will receive a small additional financial reimbursement for completing all follow-up assessments.

No files selected

Sample size

We plan to recruit a total sample of 174 participants (87 experimental, 87 control condition) at baseline (see Daemen, Postma (1). We expect an attrition rate of 6.25% at post-intervention and of 12.5% at 6-month follow-up, a 18,75% attrition rate at 18-month, and a 25% attrition rate at 24-month follow-up, which is sufficient to address our outlined hypotheses (see below).

Sample size rationale

Previous studies demonstrated that cognitive behavioral therapy (CBT) (4-6), including CBT focusing on self-esteem (7, 8), may lead to reductions in symptoms of psychopathology of moderate to large effect size. In line with previous research, the power calculation is based on the primary outcome of level of self-esteem as measured with the RSES (8), power simulation in the R environment indicated that a sample size of 130 participants (65 per condition) is sufficient to test our primary hypothesis of the effect of condition (SELFIE + TAU vs. TAU) on self-esteem, while controlling for self-esteem at baseline. Specifically, this will allow us to detect an effect size (standardized mean difference (SMD)) of 0.3 (experimental vs. control condition), i.e., a difference that is considered clinically relevant, at (at least) post-intervention or 6-month follow-up with a power of 0.87 (primary hypothesis) when testing at alpha = 0.05 using linear mixed modeling. Based on our previous and ongoing work, we will allow for a 25% attrition rate at 2-year follow-up, which will result in a loss to follow-up of around 22 individuals per condition on average.

Stopping rule

Not applicable.

Variables

Manipulated variables

• Condition
Participants are either be randomized in the experimental condition (SELFIE + TAU) or in the control condition (TAU only). Hence, will treat condition as a 2-level factor (experimental versus control). Condition will be labeled as follows: 0 – TAU only 1 – SELFIE + TAU

No files selected

Measured variables

Primary outcome
The primary outcome will be global self-esteem, measured with the Rosenberg Self-Esteem Scale (RSES) (8), across the two follow-up time points (i.e., post-intervention, 6-month follow up). The RSES is a widely used measure to assess global self-esteem with good reliability and validity (9, 10). The RSES consists of ten items rated on a 4-point Likert scale ranging from "strongly agree" to "strongly disagree". A sum score of the RSES will calculated, such that self-esteem as a continuous variable will be the primary outcome.

Secondary outcomes
Secondary outcomes will include the level of momentary, positive and negative self-esteem, resilience, positive and negative affect, emotional well-being, positive and negative schematic beliefs of self, psychological distress, functioning, subjective quality of life, general psychopathology and clinical symptoms, which will be compared between the experimental and control condition at post-intervention and 6-month follow-up (H2). In subsequent analyses, these outcomes will be compared at 18-month and 24-month follow-up (H3).

• Clinical symptoms
We will use the 24- item version of the Brief Psychiatric Rating Scale (BPRS) (11, 12) as a validated interviewer measure to assess clinical symptoms of psychopathology in youth (13). All items are rated on a 7-point scale. The intensity of psychopathological symptoms is indicated by the BRPS total score (range 24 to 168).

• General psychopathology
The revised Symptom Checklist (SCL-90-R) will be used as a reliable and valid measure to assess general psychopathology in youth (13, 14). The measure consists of 90 items, which will be rated on a 5-point scale. The total sum score of the SCL-90-R will be used for analysis.

• Psychological distress
Psychological distress will be measured with the Kessler Psychological Distress Scale (K10), which is widely used and well-validated in youth (13, 15). The K10 is a 10-item [illegible]

Ecological Momentary Intervention

EMI ESM

Experience Sampling Method

mHealth Self-esteem SELFIE

Citation

osf.io/8e4bc

This website relies on cookies to help provide a better user experience. By clicking Accept or continuing to use the site, you agree. For more information, see our Privacy Policy and information on cookie use.

Accept

• Functioning
The Social and Occupational Functioning Assessment Scale (SOFAS) (16) and the Global Assessment of Functioning (GAF) scale (17) will be used as a well-validated measure of functioning in youth (13). The overall level of functioning rated by researchers on a scale of 0 to 100 will be used in the analysis.

• Subjective quality of life
Subjective quality of life will be measured with the World Health Organization Quality of Life Instrument-Brief (WHOQOL-BREF) (13, 18). Mean scores of all four domains (physical health, psychological, social relationships, and environment) will be used.

• Positive and negative schematic beliefs of self
The Brief Core Schema Scale (BCSS) will be used as an established measure of positive and negative schematic beliefs of self and others (19). The following four total scores (all consisting of six items) will be obtained for use in the analysis: negative-self, positive-self, negative-others, and positive-others.

• Positive and negative self-esteem
Positive and negative self-esteem will be measured with the SERS, which is a 20-item rating scale to assess these two dimensions of self-esteem separately with good reliability and validity (20). The total sum score of the positive dimension and the total sum score of the negative dimension will be used in the analysis.

• Emotional well-being
We will assess emotional well-being using the Positive and Negative Affect Scale (PANAS) (21) based on the total sum score of the negative affect items and the total sum score of the positive affect items.

Secondary momentary outcomes
• Momentary self-esteem
Momentary self-esteem will be assessed with four items, rated on a 7-point scale, using the ESM (22, 23). The mean score will be used in all analyses.

• Momentary resilience will be operationalized as positive affective recovery from event-related stress in daily life (i.e., the return to baseline levels of positive affect following event-related stress) measured with ESM) (2, 24-26). In addition, an established and validated 4-item ESM measure of momentary positive affect will be used (see section 17 on ESM variables). A mean positive affect score will be used in the analysis.

• Negative affect
A 5-item ESM measure will be used for assessing negative affect (24, 25, 27). A mean score will be used in the analysis.

Acceptability, adherence, and fidelity
Participants in the experimental condition will be asked to complete a debriefing questionnaire, which assesses acceptability, satisfaction, and perceived beneficial effects of the EMI tasks and sessions. Satisfaction is measured on a 7-point Likert scale. The mean satisfaction in the intervention condition will be calculated.
Also, the Working Alliance Inventory (WAI) (28) will be completed by the participant and the SELFIE therapist providing the SELFIE intervention. The WAI for participants and SELFIE therapists both consist of 36 items, measured on a 5-point Likert scale.
Adherence to the intervention will be assessed using the implicit EMI adherence data recorded by the app (e.g., number and duration of completed EMI interactive, enhancing and consolidating tasks). Further, the attended face-to-face sessions will be audio recorded and adherence will be rated on a visual analog scale (ranging from 0 = "not at all" to 11 = "very much") by a clinical psychologist or researchers (supervised by a clinical psychologist). A mean score of compliance and adherence will be calculated.
Fidelity to the SELFIE-intervention protocol will be based on independent ratings of a random selection of audio recordings of the three face-to-face sessions and the treatment fidelity questionnaires, which will be completed by the trained therapist directly after each face-to-face session. This questionnaire will be scored on a 3-point scale. A score of 0 indicates the component is not delivered, a score of 1 indicates the component is delivered, a score of 2 indicates that the component is somewhat delivered. Descriptive statistics will be reported.

Independent variables and other measures
Independent variables
Independent variables will be time, condition, center, and baseline scores of the primary and secondary outcomes.

• Time
Assessments were completed at baseline, post-intervention, 6-month, 18-month, and 24-month follow-up. For the first manuscript focusing on outcome data collected at post-intervention, and 6-month follow-up, we will treat the time variable as a 2-level factor (post-intervention, 6-month follow-up) in our models.
Time will be labeled as follows:
0 - post-intervention
1 - 6-month follow-up

In subsequent analyses focusing on outcome data collected post-intervention, 6-month, 18-month, and 24-month follow-up, we will treat the time variable as a 4-level factor (post-intervention, 6-month, 18-month, and 24-month follow-up) in our models.
Time will be labeled as follows:
0 - post-intervention
1 - 6-month follow-up
2 - 18-month follow-up
3 - 24-month follow-up

• Condition
Participants are either randomized in the experimental condition (SELFIE + TAU) or in the control condition (TAU only). Hence, will treat condition as a 2-level factor (experimental versus control).
Condition will be labeled as follows:
0 – TAU only
1 – SELFIE + TAU

• Center
Participants are recruited at different centers in three regions in The Netherlands. Hence, will treat center as a 4-level factor.
Center will be labeled as follows:

1 – Noord-Holland
2 – Zuid-Holland
3 - Limburg
4 - general population

This website relies on cookies to help provide a better user experience. By clicking Accept or continuing to use the site, you agree. For more information, see our Privacy Policy and information on cookie use.

Accept

Other independent variables, e.g., the baseline scores of the outcome variables specified above (primary and secondary outcomes) will be included in the models as appropriate. To facilitate interpretation of our models, we will grand mean center these baseline scores.

Baseline ESM variables
Similar to the models with non-ESM outcomes, we will model the baseline scores of our ESM measures as independent variables. Given the time-varying nature of these outcomes, we will use person mean centered scores.

Cluster variable
We will assess outcome measures at three different timepoints (baseline, post-intervention, 6-month follow-up), which means that time (level 1) is clustered within participants (level 2).

ESM variables
All ESM items were measured on a 7-point Likert scale, ranging from 1 ("not at all") to 7 ("very much").

Momentary self-esteem was operationalized as the mean score of the following four ESM items: "I like myself", "I am ashamed of myself" (reversed), "I am satisfied with myself", and "I doubt myself" (reversed).

The mean score of five items was used to assess negative affect: "I feel anxious", "I feel down", "I feel uncomfortable", "I feel lonely", and "I feel insecure".

Positive affect was operationalized as the mean score of the following five ESM items "I feel happy", "I feel satisfied", "I feel relaxed", "I feel cheerful", and "I can accept my feelings".

Event-related stress was assessed with one item, that asked participants to rate the most important event since the last beep on a 7-point Likert scale ranging from "very unpleasant" (rating of -3) to " very pleasant" (rating of 3) (29). The coding of this item was recoded in order for higher ratings to indicate higher levels of stress. Ratings of -3 were coded as 7, and ratings of 3 were coded as 1.

No files selected

Indices

Momentary resilience/recovery from momentary stress was assessed with the ESM positive/negative affective recovery from event-related stress in daily life (operationalized as the return to baseline levels of positive/negative affect following the first momentary stressor of the day) (30).

No files selected

Analysis Plan

Statistical models

Reliability, factor structure and validity of the measures
• Reliability: internal consistency
The internal consistency (Chronbach's alpha) will be calculated for all (subscales of) relevant baseline measures (Rosenberg Self-Esteem Scale (RSES), Self-Esteem Rating Scale (SERS), Temperament and Characteristics Inventory (TCI), Positive and Negative Affect Scale (PANAS), The Brief Core Schema Scale (BCSS), Kessler Psychological Distress Scale (K10), World Health Organisation Quality of Life Instrument-Brief (WHOQOL-BREF), the revised Symptom Checklist (SCL-90-R) and Brief Psychiatric Rating Scale (BPRS)).
The scales for momentary self-esteem, positive affect, and negative affect, which are measured with the Experience Sampling Method (ESM), will be person-mean-centered on the person-level before calculating Cronbach's alphas, as ESM data has a hierarchical nature with time points nested within persons.

• Validity:
The research instruments used in the current study have previously been validated. Psychometric properties are reported elsewhere (1).

Feasibility and efficacy
Feasibility
Descriptive statistics will be used, and as appropriate, confidence intervals will be constructed, to summarize findings on acceptability (i.e. satisfaction), adherence, and fidelity of the SELFIE intervention. Furthermore, the sum score (based on a 5-point Likert scale) of the Working Alliance Inventory (WAI) (28), which will be completed by the participants in the experimental condition and the SELFIE therapists providing the SELFIE intervention, will be described using descriptive statistics.

Efficacy
The analysis plan for efficacy is described in detail in the attached document.

- SELFIE_Statistical Analysis Plan.pdf

Transformations

We will check whether the residuals of our fitted models are normally distributed. If residuals are non- normally distributed, we will perform transformations (e.g., log, inverse) on the dependent variables and check residuals for improvement. If residuals improve in the sense that they become normally distributed after transformation, we will re-run our models making use of a transformed outcome variable. If transformation does not lead to improvement, we will continue analyses with the original outcome variables and note the non-normally distributed residuals as a limitation.

Inference criteria

In the statistical analysis addressing hypothesis 1, we will use p-values at alpha < 0.05 (for hypothesis 2, only for Wald-type test for the main effect $\beta 2$) and construct 95% confidence intervals. In the statistical analysis addressing hypotheses 2-3, only 95% confidence intervals will be constructed and reported.

Data exclusion

No response

Missing data

In line with the intention-to-treat principle, all data of participants will be used in the analysis, including data of participants who dropped out from the intervention, and those who had low adherence. We will fit the model using Robust Restricted Maximum Likelihood (robust REML(31)) in Stata 15 (32), which allows for all available data to be used assuming that missing data is at random (33, 34). Therefore, potential bias due to [illegible]

This website relies on cookies to help provide a better user experience. By clicking Accept or continuing to use the site, you agree. For more information, see our Privacy Policy and information on cookie use.
Accept

missing data for condition and the primary outcome.

Data cleaning
The data manager and research assistants, who are blind to treatment allocation, will perform quality checks for each questionnaire as part of the data cleaning process. This means that they will perform plausibility checks, assess for every timepoint and questionnaire whether all logical rules were followed, and correct discrepancies emerging from these checks (e.g., if gender = female in questionnaire A, then gender = female in questionnaire B). Secondly, they will perform cross-checks on questionnaire(s) (items) probing on the same underlying concept (e.g., K10 versus SCL-90-R scores) and document unexpected discrepancies to give an insight in concurrent validity.

Exploratory analysis

No response

Other

Other

Abbreviations
AE: Adverse event; BCSS: Brief Core Schema Scales; BPRS: Brief Psychiatric Rating Scale; CECA: Childhood Experience of Care and Abuse; CIDI: Composite International Diagnostic Interview; CTQ: Childhood Trauma Questionnaire; DMEC: Data Monitoring and Ethics Committee; EMI: Ecological Momentary Intervention; ESM: Experience Sampling Method; EQ-5D-5L: EuroQol – 5 Dimensions – 5 Level version; K10: Kessler Psychological Distress Scale; MERC: Medical Ethics Review Committee; MUMC: Maastricht University Medical Centre; mHealth: Mobile Health; PANAS: Positive and Negative Affect Scale; RCT: Randomized controlled trial; RBQ: Retrospective Bullying Questionnaire; RSES: Rosenberg Self-Esteem Scale; SELFIE: Ecological momentary intervention for improving self-esteem; SERS: Self-Esteem Rating Scale; SOFAS: Social and Occupational Functioning Assessment Scale; TAU: Treatment as usual; TCI: Temperament and Characteristic Inventory; TiCP: Trimbos Institute and Institute of Medical Technology Assessment Questionnaire for Costs associated with Psychiatric Illness; TSC: Trial Steering Committee; WHOQOL-BREF: World Health Organization Quality of Life Instrument-Brief.

References

1. Daemen M, Postma MR, Lindauer R, Hoes-van der Meulen I, Nieman D, Delespaul P, et al. Efficacy of a transdiagnostic ecological momentary intervention for improving self-esteem (SELFIE) in youth exposed to childhood adversity: study protocol for a multi-center randomized controlled trial. Trials. 2021;22(1):641.
2. Myin-Germeys I, Oorschot M, Collip D, Lataster J, Delespaul P, van Os J. Experience sampling research in psychopathology: opening the black box of daily life. Psychological medicine. 2009;39(9):1533-47.
3. Reininghaus U, Depp CA, Myin-Germeys I. Ecological Interventionist Causal Models in Psychosis: Targeting Psychological Mechanisms in Daily Life. Schizophrenia bulletin. 2016;42(2):264-9.
4. Rauschenberg C, Böcking B, Pätzold I, Deckers D, Schruers K, Heunen I, et al. An ecological momentary compassion-focused intervention for enhancing resilience in help-seeking youths: a pilot study. PsyArXiv. 2020.
5. Hunot V, Moore TH, Caldwell DM, Furukawa TA, Davies P, Jones H, et al. 'Third wave'cognitive and behavioural therapies versus other psychological therapies for depression. Cochrane Database of Systematic Reviews. 2013(10).
6. Johns LC, Oliver JE, Khondoker M, Byrne M, Jolley S, Wykes T, et al. The feasibility and acceptability of a brief Acceptance and Commitment Therapy (ACT) group intervention for people with psychosis: the 'ACT for life'study. Journal of behavior therapy and experimental psychiatry. 2016;50:257-63.
7. Staring T. Self-Esteem Treatment in Anxiety (SETA) trial. GZ-Psychologie. 2014;6(5):10-2.
8. Staring AB, van den Berg DP, Cath DC, Schoorl M, Engelhard IM, Korrelboom CW. Self-esteem treatment in anxiety: A randomized controlled crossover trial of Eye Movement Desensitization and Reprocessing (EMDR) versus Competitive Memory Training (COMET) in patients with anxiety disorders. Behav Res Ther. 2016;82:11-20.
9. Everaert J, Koster EHW, Schacht R, De Raedt R. Evaluatie van de psychometrische eigenschappen van de Rosenberg zelfwaardeschaal in een poliklinische psychiatrische populatie. Gedragstherapie. 2010;43:307-17.
10. Schmitt P, Allik J. Simultaneous administration of the Rosenberg self-esteem scale in 53 nations: Exploring the universal and culture specific features of global self-esteem. Journal of Personality and Social Psychology. 2009;89:623-42.
11. Lukoff D, Liberman RP, Nuechterlein KH. Symptom monitoring in the rehabilitation of schizophrenic patients. Schizophrenia bulletin. 1986;12(4):578-602.
12. Overall JE, Hollister LE, Pichot P. Major psychiatric disorders. A four-dimensional model. Archives of general psychiatry. 1967;16(2):146-51.
13. Kwan B, Rickwood DJ. A systematic review of mental health outcome measures for young people aged 12 to 25 years. BMC psychiatry. 2015;15:279.
14. Derogatis LR. SCL-90-R, administration, scoring & procedures manual-I for the R(evised) version. Baltimore, MD: Johns Hopkins University School of Medicine; 1977.
15. Kessler RC, Andrews G, Colpe LJ, Hiripi E, Mroczek DK, Normand SL, et al. Short screening scales to monitor population prevalences and trends in non-specific psychological distress. Psychol Med. 2002;32(6):959-76.
16. Goldman HH, Skodol AE, Lave TR. Revising axis V for DSM-IV: a review of measures of social functioning. The American journal of psychiatry. 1992;149(9):1148-56.
17. Birchwood M, Smith J, Cochrane R, Wetton S, Copestake S. The Social Functioning Scale. The development and validation of a new scale of social adjustment for use in family intervention programmes with schizophrenic patients. The British journal of psychiatry : the journal of mental science. 1990;157:853-9.
18. Whoqol Group. Development of the World Health Organization WHOQOL-BREF quality of life assessment. Psychological medicine. 1998;28(3):551-8.
19. Fowler D, Freeman D, Smith B, Kuipers E, Bebbington P, Bashforth H, et al. The Brief Core Schema Scales (BCSS): psychometric properties and associations with paranoia and grandiosity in non-clinical and psychosis samples. Psychological medicine. 2006;36(6):749-59.
20. Lecomte T, Corbiere M, Laisne F. Investigating self-esteem in individuals with schizophrenia: relevance of the Self-Esteem Rating Scale-Short Form. Psychiatry research. 2006;143(1):99-108.
21. Watson D, Clark LA, Tellegen A. Development and validation of brief measures of positive and negative affect: the PANAS scales. J Pers Soc Psychol. 1988;54(6):1063-70.
22. Thewissen V, Bentall RP, Lecomte T, van Os J, Myin-Germeys I. Fluctuations in self-esteem and paranoia in the context of daily life. Journal of abnormal psychology. 2008;117(1):143-53.
23. Thewissen V, Bentall RP, Oorschot M, J AC, van Lierop T, van Os J, et al. Emotions, self-esteem, and paranoid episodes: an experience sampling study. Br J Clin Psychol. 2011;50(2):178-95.
24. Reininghaus U, Gayer-Anderson C, Valmaggia L, Kempton MJ, Calem M, Onyejiaka A, et al. Psychological processes underlying the association between childhood trauma and psychosis in daily life: an experience sampling study. Psychological medicine. 2016;46(13):2799-813.

This website relies on cookies to help provide a better user experience. By clicking Accept or continuing to use the site, you agree. For more information, see our Privacy Policy and information on cookie use.

Accept

26. Vaessen T, Viechtbauer W, van der Steen Y, Gayer-Anderson C, Kempton MJ, Valmaggia L, et al. Recovery from daily-life stressors in early and chronic psychosis. Schizophrenia research. 2019;213:32-9.
27. Myin-Germeys I, van Os J, Schwartz JE, Stone AA, Delespaul PA. Emotional reactivity to daily life stress in psychosis. Archives of general psychiatry. 2001;58(12):1137-44.
28. Horvath AO, Greenberg LS. Development and validation of the Working Alliance Inventory. Journal of counseling psychology. 1989;36(2):223.
29. Myin-Germeys I, van Os J, Schwartz JE, Stone AA, Delespaul PA. Emotional reactivity to daily life stress in psychosis. Archives of general psychiatry. 2001;58(12):1137-44.
30. Vaessen T, Viechtbauer W, van der Steen Y, Gayer-Anderson C, Kempton MJ, Valmaggia L, et al. Recovery from daily-life stressors in early and chronic psychosis. Schizophrenia research. 2019;213:32-9.
31. Richardson AM, Welsh AH. Robust restricted maximum likelihood in mixed linear models. Biometrics. 1995:1429-39.
32. StataCorp L. Stata statistical software: Release 15 (2017). College Station, TX: StataCorp LP. 2017.
33. StataCorp L. Stata survival analysis reference manual. 2017.
34. Little RJ, Rubin DB. Statistical analysis with missing data: John Wiley & Sons; 2019.

Copyright © 2011-2023 Center for Open Science | Terms of Use | Privacy Policy | Status | API
TOP Guidelines | Reproducibility Project: Psychology | Reproducibility Project: Cancer Biology

This website relies on cookies to help provide a better user experience. By clicking Accept or continuing to use the site, you agree. For more information, see our Privacy Policy and information on cookie use.

Accept

Algemene gegevens / General Information

Programma / Programme	:	**OnderzoeksProgramma GGz**
Subsidieronde / Subsidy round	:	**Onderzoeksprogramma GGz - Middellanglopend Onderzoek**
Projecttitel / Project title	:	**Improving self-esteem in traumatized youth: a transdiagnostic ecological momentary intervention trial (SELFIE)**
Projecttaal / Project language	:	**Engels / English**
Geplande startdatum / Planned start date	:	**01-12-2017**
Geplande duur / Planned duration	:	**48 maanden / months**
Datum indienen / Date of application	:	**11-04-2017**
Projecttype / Project type	:	**Anders / Differently**
Vervolg eerder ZonMw-project / Continuation previously funded project ZonMw	:	**Nee / No**

Projectleden / Project members

Dr. U. Reininghaus (Main applicant)
Functie / Position: Assistant Professor | *Opleiding / Education:*
Studierichting / Subject:
T: 0433883896 | *F:* | *E:* u.reininghaus@maastrichtuniversity.nl

Maastricht University Medical Center
Psychiatrie en Neuropsychologie

Postbus 616
6200 MD MAASTRICHT

Prof. dr. T. van Amelsvoort (Projectleader and secretary)
Functie / Position: Professor | *Opleiding / Education:*
Studierichting / Subject:
T: 0433883509 | *F:* | *E:* t.vanamelsvoort@maastrichtuniversity.nl

Maastricht University Medical Center
Psychiatrie en Neuropsychologie

Postbus 616
6200 MD MAASTRICHT

Prof. dr. N. de Vries (Administrative responsibility)
Functie / Position: Dean | *Opleiding / Education:*
Studierichting / Subject:
T: 0433871329 | *F:* | *E:* n.devries@maastrichtuniversity.nl

Maastricht University
Caphri - School for Public Health and Primary Care
Department of Health Promotion

Postbus 616
6200 MD MAASTRICHT

Prof. dr. M. van der Gaag (Co-Applicant)
Functie / Position: Professor | *Opleiding / Education:*
Studierichting / Subject:
T: 0645780463 | *F:* | *E:* m.van.der.gaag@me.com

Parnassia
Psychose onderzoek

Zoutkeetsingel 40
2512 HN DEN HAAG

Prof. dr. R. Lindauer (Co-Applicant)
Functie / Position: Professor | *Opleiding / Education:*
Studierichting / Subject:
T: 0206168174 | *F:* | *E:* R.Lindauer@debascule.com

Academisch Medisch Centrum
Kinder- en Jeugdpsychiatrie
Onderzoek en Opleiding

Meibergdreef 5
1105 AZ AMSTERDAM ZUIDOOST

Dr. D. Nieman (Co-Applicant)
Functie / Position: Head, Cognition Lab | *Opleiding / Education:*
Studierichting / Subject:
T: 0208913683 | *F:* | *E:* d.h.nieman@amc.nl

Academisch Medisch Centrum
Department of Psychiatry
Early Psychosis

Meibergdreef 5
1105 AZ AMSTERDAM ZUIDOOST

Mr. G. de Roos (Co-Applicant)
Functie / Position: Directeur-bestuurder | *Opleiding / Education:*
Studierichting / Subject:
T: 0612472215 | *F:* | *E:* gerard@ixtanoa.nl

Ixta Noa

Parkstraat 31
6828 JC ARNHEM

Dr. B. Rutten (Co-Applicant)
Functie / Position: Head, Division of Neuroscience | *Opleiding / Education:*
Studierichting / Subject:
T: 0433881348 | *F:* | *E:* b.rutten@maastrichtuniversity.nl

Maastricht University
Psychiatry and Neuropsychology
Neuroscience

Universiteitssingel 50
6229 ER MAASTRICHT

Prof. dr. K. Schruers (Co-Applicant)
Functie / Position: Professor | *Opleiding / Education:*
Studierichting / Subject:
T: 0433883511 | *F:* | *E:* koen.schruers@maastrichtuniversity.nl

Maastricht University Medical Center
Psychiatrie en Neuropsychologie

Postbus 616
6200 MD MAASTRICHT

Dr. G. Tuijthof (Co-Applicant)
Functie / Position: Lector | *Opleiding / Education:*
Studierichting / Subject:
T: +31 (0)6 24 58 55 15 | *F:* | *E:* gabrielle.tuijthof@zuyd.nl

Zuyd
Bèta Sciences and Technology
Smart devices

Nieuw-Eyckholt 300
6419 DJ HEERLEN

Projectgegevens / Project information

Samenvatting / Summary

The majority of mental disorders first emerge in youth and, as such, contribute substantially to disease burden. Three quarters of adult mental disorders emerge before the age of 25, and 50% before the age of 16. This onset phase disrupts critical age-specific developmental, interpersonal, occupational and educational milestones and indicates a need for close scrutiny of the complex interplay between risk and protective factors in childhood, and the value of a preventive intervention to improve well-being, enhance resilience and prevent morbidity later in life. Evidence has accrued linking childhood trauma as a major risk factor, with a range of mental disorders via pathways through self-esteem. Therefore, targeting low self-esteem in youth exposed to childhood trauma is a promising strategy for preventing adult mental disorder, but our current psychological help strategies remain difficult to access and accept for youth, calling for novel, youth-friendly approaches. The recent rapid

advances in information and communication technologies have led to the development of mobile Health (mHealth) and, most prominently, ecological momentary interventions (EMIs), which provide a unique opportunity to deliver youth-friendly, personalized, real-time, guided self-help interventions. The current study aims to minimize the deleterious impact of childhood trauma through testing the efficacy of a novel, accessible, transdiagnostic ecological momentary intervention for improving self-esteem (‘SELFIE’) in help-seeking youth with prior exposure to childhood trauma.
In an exploratory randomized controlled trial, youth aged 12-25 with prior exposure to childhood trauma referred to mental health services across the Netherlands (Virenze, Mondriaan, Parnassia, Bascule, or Amsterdam Medical Centre) will be randomly allocated to the SELFIE in addition to treatment as usual (TAU) (experimental condition) or to TAU only (control condition). Data will be collected before randomization (‘baseline’), at the end of the 6-week intervention period (‘post-intervention’), and after a 6-month, 18-month, and 2-year follow-up period (‘follow-up’). Participants allocated to the TAU (control) condition will continue to receive all the treatment they received prior to the start of the study. Subjects allocated to the experimental condition will receive the manualised SELFIE with a trained clinical psychologist within a 6-week period after randomization in addition to TAU. The intervention will consist of three sessions with a trained clinical psychologist, on-demand e-mail contact, and the SELFIE using a guided self-help approach administered through a smartphone-based PsyMate® App to allow for interactive, personalized, real-time and real-world transfer of intervention components in individuals’ daily lives. Primary outcomes will be the level of self-esteem as measured with Ecological Momentary Assessment using the PsyMate® App and the Rosenberg Self-Esteem Scale. Secondary outcomes will be resilience, emotional well-being, social functioning, quality of life and general psychopathology. Given that exposure to trauma and response to treatment may be reflected at the biological level in distinct epigenetic signatures, levels of DNA methylation in NR3C1 (i.e. glucocorticoid receptor 1 gene) and SLCA4 (i.e. the serotonin transporter gene) will be measured using targeted sequencing.

Trefwoorden / Keywords

youth mental health; mobile Health (mHealth); ecologcial momentary intervention; self-esteem; childhood trauma; prevention; transdiagnostic; randomized controlled trial

Samenwerking / Collaboration

Samenwerking tussen onderzoek en praktijk / Cooperation between research and practice:

Ja / Yes

Organisaties

Academisch Medisch Centrum
Kind en volwassenen psychiatrie

Meibergdreef 9
1105 AZ AMSTERDAM ZUIDOOST

De Bascule

Meibergdreef 5
1105 AZ AMSTERDAM ZUIDOOST

Mondriaan Zorggroep
Verslavingszorg

Postbus 3051
6202 NB MAASTRICHT

Parnassia
Psychose onderzoek

Zoutkeetsingel 40
2512 HN DEN HAAG

Virenze

Wilhelminasingel 114
6221 BL MAASTRICHT

Inhoud / Content

Probleemstelling / Problem definition

The majority of mental disorders first emerge in youth and, as such, contribute substantially to disease burden which is higher in youth than during any other period of life (Copeland et al., 2011, Harhay and King, 2012, Kessler et al., 2005, Kim-Cohen et al., 2003). More specifically, 75% of adult mental disorders emerge before the age of 25, and 50% before the age of 16 (Kessler et al., 2005). This onset phase disrupts critical age-specific developmental, interpersonal, occupational and educational milestones and indicates a need for close scrutiny of the complex interplay between risk and protective factors in childhood, and the value of a preventive intervention to improve well-being, enhance resilience and prevent morbidity later in life. Therefore, interventions aimed at preventing mental health problems should be targeting youth.

Youth referred to mental health services have experienced disproportionate levels of childhood trauma (Chen et al., 2010, Frissen et al., 2015, Kraan et al., 2015a, Kraan et al., 2015b, Kraan et al., 2017, Matheson et al., 2013, Varese et al., 2012), which is one of the most pervasive risk factors for developing a range of psychiatric disorders (Kessler et al., 2010, Matheson et al., 2013). So for example, Kraan et al. (2015), in their recent study of 125 help-seeking adolescents and young adults with an Ultra-High-Risk state for psychosis (UHR) in Amsterdam, reported that a staggering 75% of these individuals were exposed to at least one form of childhood trauma including physical, sexual and emotional abuse as well as physical and emotional neglect (Kraan et al., 2015a). Similarly, in a nation-wide Dutch study of help-seeking adolescents and young adults with UHR conducted in the Hague, Amsterdam, Leiden and Friesland, a high prevalence was found for physical (20.9%), sexual (24.8%) and emotional (46.7%) abuse, and physical (41.9%) and emotional (66.7%) neglect (Kraan et al., 2017). However, primary prevention of childhood trauma through universal, population-based strategies remains difficult to achieve. Thus, screening for, and tackling the negative consequences of, childhood trauma in youth is a promising selective prevention strategy for adverse outcomes later in life.

One important psychological mechanism in pathways from childhood trauma to adult psychopathology is low self-esteem (Brown et al., 2008, Garety et al., 2007). Youth is a critical period for the development of self-esteem. Self-esteem is essential to well-being and mental health per se, with a substantial impact on the development and maintenance of severe mental disorders (Zeigler-Hill, 2011). There is now substantial evidence to suggest that exposure to childhood trauma has disastrous effects on self-esteem (Banyard et al., 2001, Kamsner and McCabe, 2000, Mannarino and Cohen, 1996, Murthi and Espelage, 2005) and that it exerts its detrimental effects on risk of later psychopathology precisely via pathways through low self-esteem (Brown et al., 2008, Fisher et al., 2012, Fisher et al., 2013, Reininghaus et al., 2014). Thus, targeting low self-esteem in youth exposed to childhood trauma has the potential to prevent the onset of adult mental disorder.

Our current psychological help strategies, however, remain difficult to access and accept for youth and have limited efficacy under real-world conditions, calling for novel approaches (Malla et al., 2016, McGorry et al., 2013). This has now been recognized in a number of countries as reflected by an ongoing mental health reform for youth aged 12-25 years to ensure a smooth transition from child and adolescent to adult mental health services (Malla et al., 2016, McGorry et al., 2013). There is a pressing need for youth-friendly, highly accessible, individually tailored and effective interventions that minimize the impact of, and enhance resilience to, childhood trauma. In recent years, it has become increasingly evident how childhood adversity impacts on underlying epigenetic mechanisms to increase risk of mental disorders (Pishva et al., 2012, Pishva et al., 2014, Reininghaus and Morgan, 2014, Rutten and Mill, 2009). Epigenetics has been shown to play a decisive role in the neuronal adaptations underlying the response to environmental changes (McGowan et al., 2009, Turecki and Meaney, 2016) and the pathogenesis and treatment of mental disorders (Vialou et al., 2013). In fact, exposure to trauma has been linked to distinct epigenetic profiles (McGowan et al., 2009, Turecki and Meaney, 2016), and pathogenesis of psychiatric disorders (Vialou et al., 2013). Based on these and other findings, it has been proposed that regaining well-being after trauma may be mediated via epigenetic mechanisms. However, it remains unknown whether changes resulting from interventions preventing or restoring mental health in traumatized individuals are also reflected at the epigenetic level.

Relevantie / Relevance

The majority of mental disorders emerge in youth (Copeland et al., 2011, Kessler et al., 2005, Kim-Cohen et al., 2003), allowing such disorders to affect the entire lifespan. Indeed, mental disorders in youth aged 10-24 years are associated with an immense cost (Mangalore and Knapp, 2007, McCrone et al., 2008, The Schizophrenia Commission, 2012) and, as such, the leading cause of disease burden in high-income countries (Harhay and King, 2012). Therefore, interventions aimed at preventing mental health problems should be targeting youth in order to reduce burden for individuals, families and the wider society.

High levels of childhood trauma have recently been reported in youth presenting to mental health services (Chen et al., 2010, Frissen et al., 2015, Kraan et al., 2015a, Kraan et al., 2015b, Kraan et al., 2017, Matheson et al., 2013, Varese et al., 2012), which are, in turn, associated with an increased risk of developing a range of psychiatric disorders in adulthood (Kessler et al., 2010, Matheson et al., 2013). Current estimates of attributable risks suggest that interventions targeted at averting childhood trauma from exerting its adverse effects can prevent a substantial proportion of the incidence of adult mental disorder, and, thereby, have a sizeable public health impact and reduce societal costs (Kirkbride and Jones, 2011, Varese et al., 2012). While evidence on the primary prevention of childhood trauma remains limited, interventions targeting the negative psychological consequences of childhood trauma in youth are a viable strategy with tangible public health implications (Reininghaus et al., 2016b, Reininghaus et al., 2014). The prevalence and impact of childhood trauma on later psychopathology tends to be greater in women than in men (Fisher et al., 2009, Pereda et al., 2009) and in migrant and ethnic minority than in ethnic majority groups (Morgan et al., 2010). Further, childhood trauma has detrimental effects on self-esteem (Banyard et al., 2001, Kamsner and McCabe, 2000, Mannarino and Cohen, 1996, Murthi and Espelage, 2005), and leads to self-blaming attributions, particularly in girls (Mannarino and Cohen, 1996). The prevalence of low self-esteem in help-seeking youth has been reported to be around 45%, with a higher prevalence reported in girls (54%) than in boys (33%) (Overholser et al., 1995). In recent years, evidence has accrued linking childhood trauma with a range of psychiatric disorders via pathways through self-esteem (Brown et al., 2008, Fisher et al., 2012, Fisher et al., 2013, Reininghaus et al., 2014). Therefore, targeting low self-esteem in youth exposed to childhood trauma is a promising strategy for preventing adult mental disorder and reducing societal costs.

It is also only now that we are beginning to understand that a reform of mental health services is urgently needed that aims to provide a seamless coverage of mental health care with smooth transitions from adolescence to mature adulthood at around 25 years of age involving both child and adolescent and adult mental health services in order to reduce disease burden (Malla et al., 2016, McGorry et al., 2013). While conventional interventions have proven efficacious in reducing psychiatric symptoms via enhancing self-esteem (Staring et al., 2016), a key next step is to develop and evaluate interventions that are specifically

geared toward the specific needs of youth. This is what the proposed research is designed to achieve.

The recent advances in information and communication technologies have led to the development of mobile Health (mHealth) and, most prominently, ecological momentary interventions (Dinesen et al., 2016, Heron and Smyth, 2010, Myin-Germeys et al., 2016, Reininghaus et al., 2016a), which provide a unique opportunity to deliver youth-friendly, accessible, personalized, real-time, guided self-help interventions targeting candidate psychological mechanisms in daily life and, thereby prevent adult mental disorder and reduce disease burden. This enables youth to access interventions that are individually tailored to their needs in a given moment and context (e.g., by offering interventions specifically tailored for helping participants in moments of low self-esteem). EMIs are amenable to enhancing access to mental health services for youth depending on their needs and preferences by delivering low-threshold interventions by frontline mental health staff as one component that can be rolled out across child, adolescent and adult mental health services.

Furthermore, to date it is still impossible to predict who will and who will not benefit from therapy. Epigenetic changes represent the biological mechanism underlying behavioural change and also improvement following therapy. Thus, epigenetics hold the possibility to provide an index of prediction of success, originating from the underlying biological mechanisms of psychological therapy. Therefore, we advocate investigating this in the proposed research.

Kennisoverdracht, implementatie, bestendiging / Knowledge transfer, Implementation Consolidation

The proposed research will provide evidence on the clinical efficacy and cost effectiveness, which will inform national and international guidelines for delivering mental health care and contribute to the youth mental health reform that is currently under way in several countries. In the Netherlands, this is reflected in the implementation of the Australian Headspace model in several cities, in which members of our research team are centrally involved. The ecological momentary intervention for improving self-esteem (‘SELFIE’) investigated as part of the proposed research (see Bijlage 4, Figure 1 for an overview) will be implemented in routine service of five major mental health care organizations in the Netherlands (Virenze, Mondriaan, Parnassia, Bascule, or Academic Medical Centre (AMC)) and will be delivered by frontline mental health staff at a low cost such that it can be easily extended to other clinical partners.

The SELFIE intervention using the PsyMate® App will be entirely novel and will, as such, require collecting new data (which will be combined with other large sets of EMI data from the research team and their academic partners for optimal use) and will not substitute any existing processes or services. Therefore, design, programming, server and support required for delivering the intervention provided by PsyMate® will form part of the implementation phase of the project in frontline mental health services in the beginning of the project (month 1-6; see Bijlage 4, Figure 2, ‘Timeline and milestones of the proposed research’). In response to the comments by the commission of the Onderzoeksprogramma GGZ, we will involve youth from Ixta Noa as representatives of the target group in the development of the SELFIE intervention drawing on a user-centered design approach in month 1-6 of the project in order to adopt the design of the PsyMate® App and optimise its acceptability and usability for youth. This will involve an iterative process of 3 sessions, in which, first, the needs and preferences of youth are identified, second, a mock-model will be designed and assessed on acceptability and usability by youth, and, third, actual implementation of the optimal design, which will be tested for real-life use by youth (Hsieh and Shannon, 2005, Nielsen and Molich, 1990). As a consequence of allowing more time at the set out of the proposed project, we have reduced the follow-up period from a 3-year to a 2-year period in order to ensure feasibility of recruitment and assessment within the given budget and time frame (4 years).

The training and supervision of therapists required for ensuring fidelity to treatment protocol will contribute to knowledge transfer and implement this intervention in services provided by our clinical partners. If the proposed research generates evidence in support of the efficacy and cost effectiveness (Rojas and Gagnon, 2008) of the SELFIE intervention, this will provide the basis for implementing this intervention by other clinical partners and rolling it out to child and adolescent as well as adult mental health services on a nation-wide basis.

Doelstelling / Objective

The overall aim of the current study is to minimize the impact of childhood trauma through testing a novel, accessible, transdiagnostic ecological momentary intervention (EMI) for improving self-esteem (‘SELFIE’) (see Bijlage 4, Figure 1 for an overview) in help-seeking youth aged 12-25 with prior exposure to childhood trauma.

The proposed research will test the clinical efficacy of the SELFIE intervention and investigate underlying epigenetic mechanisms in an exploratory randomized controlled trial: the SELFIE intervention will be administered in addition to treatment as usual (TAU) (experimental condition) as compared to a control condition of TAU only in help-seeking youth exposed to childhood trauma.

The specific objectives are:

1. To test the efficacy of the SELFIE intervention on improving self-esteem (primary outcome), resilience, emotional well-being, functioning, quality of life and general psychopathology (secondary outcomes);

2. To examine whether the effects on resilience, emotional well-being, functioning, quality of life, and general psychopathology are mediated via self-esteem;

3. To test whether effects on primary and secondary outcomes hold true at 6-month, 18-month, and 2-year follow-up;

4. To test the efficacy of the SELFIE intervention on reducing incidence of clinical symptoms of psychopathology as compared

to the control condition at 6-month, 18-month, and 2-year follow-up;

5. To examine the cost effectiveness of the SELFIE intervention;

6. To examine epigenetic mechanisms underlying changes in primary and secondary outcomes; and

7. To predict who will preferentially respond to the SELFIE intervention via epigenetic screening.

Plan van Aanpak / Strategy

Study design
In an exploratory RCT, youth aged 12-25 with prior exposure to childhood trauma who have been referred to mental health services will be randomly allocated to the SELFIE intervention in addition to treatment as usual (TAU) (experimental condition) or to TAU only (control condition). Data will be collected before randomization ('baseline'), at the end of the 6-week intervention period ('post-intervention'), and after a 6-month, 18-month, and 2-year follow-up period ('follow-up') (see Bijlage 4, Figure 2, 'Timeline and milestones of the proposed research'). All outcomes will be measured in all participants at all time points. Randomization will be conducted independently of the research team through a computer-generated sequence. The assessors (i.e. PhD student, research assistant) will be blind to the allocation of subjects when assessing participants at post-intervention and follow-up. After random allocation, the names and contact details of the patients will be passed on to the trained clinician running the SELFIE sessions. This will be done through an independent researcher (i.e., the research coordinator) and not the assessors so that they can maintain masking for post-intervention and follow-up assessments. The trial cannot be fully "blind" because the trained clinician and patients cannot be masked towards the allocation of the patients to the experimental or control group. However, researchers will be masked towards the allocation of patients when assessing eligibility and baseline, post-intervention and follow-up scores. Any data specific to the intervention group (e.g., on treatment fidelity) will be stored in a separate database. For any breaks in masking, this will be documented in the trial master file and another assessor will be will be allocated to complete the next set of assessments where possible.

Justification of the design: Previous studies of conventional interventions suggest that psychiatric symptoms may be reduced through enhancing self-esteem (Staring et al., 2016). However, these interventions are not tailored toward the specific preferences and needs of youth as naturally occurring in daily life. While EMIs such as the SELFIE intervention provide a unique opportunity to deliver youth-friendly, accessible, personalized, real-time interventions in daily life, robust trial-based evidence on EMIs and other mHealth interventions remains very limited (Bakker et al., 2016, Myin-Germeys et al., 2016, Reininghaus et al., 2016a, Steinhart et al., in press). A key next step, therefore, is to examine, in a RCT design, the efficacy of the SELFIE intervention and, thereby, prevent adverse outcomes later in life.

Intervention

Control condition (TAU only):
Participants allocated to the TAU (control) condition will continue to receive all the treatment they received prior to the start of the study. This will include good standard care delivered according to local and national service guidelines and protocols by their psychiatrist and other members of the mental health care team. Service contacts will be assessed for the duration of the trial using the Client Service Receipt Inventory (CSRI) (Chisholm et al., 2000) to monitor variation in delivery of, and engagement with, mental health services. Informed consent to participate will include agreement not to initiate any new psychological talking therapies during the intervention period.

Experimental condition (SELFIE + TAU):
Subjects allocated to the experimental condition will receive the manualised SELFIE with a trained clinical psychologist within a 6-week period in addition to TAU (see Bijlage 4, Figure 1 'Key components of SELFIE intervention' for an overview). The intervention will consist of three sessions with a trained clinical psychologist (1 introductory session, 1 follow-up 'booster' session four weeks later, and a review session at the end of the 6-week intervention period), on-demand e-mail contact, and the SELFIE using a guided self-help approach administered through a smartphone-based PsyMate® App to allow for interactive, personalized, real-time and real-world transfer of intervention components in individuals' daily lives. The PsyMate® App is at the cutting edge of current standards of data safety and confidentiality, preventing access to data by any third party.

Delivering the intervention in individuals' daily lives, and enabling youth to benefit from this intervention in a given moment and context, when most needed (e.g. in moments of low self-esteem) is the key goal of the 6-week SELFIE intervention. Therefore, in the first introductory session, participants will be given the smartphone-based App by the trained clinical psychologist, who will explain the SELFIE intervention in detail and ask the participant to complete examples of training tasks on the App. During the 6-week intervention period, the dedicated electronic device will beep six times per day on four days per week. Each time this device beeps participants will be asked to complete a brief smartphone-based, ecological momentary assessment (EMA) of momentary self-esteem, stress, positive and negative affect, and socio-environmental context in daily life, with EMAs scheduled at random within variable blocks of time agreed with participants using a variable stratified random sampling schedule to match young participants' schedules and daily routines. In order to keep assessment burden for participants to a minimum, we have restricted the EMA to six measurements per day on four days per week and now use a sampling schedule that allows matching young participants' schedules and daily routines. The App will offer participants enhancing, consolidating, interactive and personalized EMI training tasks. Specifically, they will be asked to complete one 'enhancing task' per day, addressing self-selected goals through customized tasks in the component covered in the respective week. Participants will further be offered 'consolidating tasks' each time the smartphone beeps (i.e., covering components from previous weeks). The components covered by these tasks will be extended each week until all components are included in the consolidating tasks.

Interactive tasks will be offered if participants score low on self-esteem. These tasks will be specifically tailored for helping participants in moments of low self-esteem. Personalized tasks will be offered if participants are exposed to specific social situations or socio-environmental contexts (e.g., home, workplace, public places) associated with low self-esteem identified in the introductory or booster therapy sessions.
Acceptability and feasibility of EMIs: The SELFIE intervention is based on principles of EMIs, which have been successfully administered and found to be both acceptable and feasible in samples of individuals with depression (Kramer et al., 2014) and psychotic disorder (Granholm et al., 2012) as well as youth with subclinical symptoms of psychopathology (NTR No. NTR3808) (Lange et al., 2016).

Sample
In order to ensure feasibility and allow for a 25% attrition rate, we will recruit an initial sample of 174 participants seeking help from five different mental health care organizations (i.e., Virenze, Mondriaan, Parnassia, Bascule, or Academic Medical Centre (AMC)), and who have been previously exposed to childhood trauma, leaving n=130 participants at 2-year follow-up for our primary analysis. Individuals aged 12-25 (McGorry et al., 2013) presenting to these services will be screened with the Childhood Trauma Questionnaire (CTQ) (Bernstein and Fink, 1998, Scher et al., 2001, Wright et al., 2001), and the peer bullying subscale of the Social Contexts and Experiences measure (EU-GEI, 2014, Hubbard et al., 2015).

Inclusion criteria will be:
• Aged between 12 and 25 years, which is consistent with national and international efforts for a youth mental health reform (Malla et al., 2016, McGorry et al., 2013)
• Prior exposure to childhood trauma defined as moderate or severe physical, sexual and emotional abuse, emotional and physical neglect, according to established severity categories of the CTQ (Bernstein and Fink, 1998, Scher et al., 2001, Wright et al., 2001), and moderate or severe peer bullying, measured with the Social Contexts and Experiences measure (EU-GEI, 2014, Hubbard et al., 2015)
• Referral to Virenze, Mondriaan, Parnassia, Bascule or AMC
• Willingness to participate in the SELFIE intervention
• Ability to give written informed consent
• Parental consent for minors

Exclusion criteria will be:
• Insufficient command of Dutch so that the SELFIE intervention cannot be followed and outcomes cannot be reasonably assessed in Dutch
• Severe endocrine, cardiovascular or brain disease

Justification of sample size: In planning, we have paid particularly close attention to establishing the feasibility of recruitment strategies for this study. These build on our previous and ongoing work with adolescents and young adults and are based on conservative targets derived from liaising with clinical services at Virenze, Mondriaan, Parnassia, Bascule or AMC. They are in line with calls for pragmatic RCTs to reflect the heterogeneity of patient populations encountered in clinical practice (Hotopf, 2002). At least 2-3 individuals aged 12-25 are referred, on average, to each of the five sites (Virenze, Mondriaan, Parnassia, Bascule, AMC) per week (i.e. a total of 10-15 individuals across all sites). Hence, even if only a very conservative estimate of 30% (i.e., around 3-5 individuals) of these screen positive on the CTQ (a markedly lower estimate than what has been previously reported in help-seeking youth (Kraan et al., 2015a, Kraan et al., 2017, Reininghaus et al., 2016b, Thompson et al., 2009), it will be feasible to recruit 3-4 participants per week and meet our target of including 174 participants within the first 15 months of recruitment (i.e., around 60 working weeks) to allow for a 2-year follow-up assessment in all participants, with sufficient flexibility for contingencies (see Bijlage 4, Figure 2, ‘Timeline and milestones of the proposed research’). We will further ensure that research staff (1 PhD student, 1 research assistant) is physically present at each of the academic centres and clinical services for ongoing engagement, recruitment and data collection to make sure this target is met. Power simulation in Stata 14.0 indicates that a sample size of 130 participants (65 per condition) is sufficient for detecting an effect size of 0.3 (experimental vs. control condition) for the main effect of group on self-esteem with a power of 0.81 at $p<0.05$ using linear mixed modelling (see below). In response to the comments by the cliëntenpanel suggesting a lower age limit (i.e., age 12) and by the commission of the Onderzoeksprogramma GGZ asking for a more detailed description of the feasibility of the study, we have a) extended recruitment to child and adolescent mental health services at Virenze, Mondriaan, Parnassia, Bascule and AMC and b) increased the initial sample size to 174 participants in order to ensure feasibility and allow for a 25% attrition rate at 2-year follow-up (resulting in a loss to follow-up of around 22 individuals per condition on average); therefore, we will recruit a total sample of 174 participants, which leaves n=130 participants at follow-up to detect an effect size of 0.3 (experimental vs. control condition) for the main effect of group on self-esteem. Also, in response to the comments by the commission of the Onderzoeksprogramma GGZ asking to involve the target group in the development of the SELFIE intervention, we will now closely involve youth from Ixta Noa in month 1-6 of the project and have, therefore, reduced the follow-up period from a 3-year to a 2-year period in order to ensure feasibility of recruitment and assessment within the given budget and time frame (4 years). In addition, we will over-sample men as well as individuals from migrant and ethnic minority groups (in particular, in the Amsterdam and The Hague centres to allow for a priori planned subgroup analysis according to established credibility criteria (Sun et al., 2010) for investigating the effects of SELFIE in men (and individuals from migrant and ethnic minority groups, respectively) compared with women (and individuals from the Dutch ethnic majority groups. Results from earlier epigenetic studies by our and other groups (Rutten et al., 2014a, Vinkers et al., 2015, Yehuda et al., 2013) and the estimated effect on self-esteem suggest that the present study will have sufficient power to detect associations in epigenetic changes over time with the changes in self-esteem.

Data collection

Screening:
Childhood abuse and neglect: We will use the Childhood Trauma Questionnaire (CTQ) (Bernstein and Fink, 1998), which is a self-report measure to assess the severity of the most common types of abuse (i.e., physical, sexual and emotional abuse) and neglect (i.e., physical and emotional neglect) before the age of 16 and allows to compute categorical severity scores of none, mild, moderate, and severe. Good psychometric properties have been reported for the CTQ in clinical as well as community samples (Scher et al., 2001, Wright et al., 2001).
Peer bullying: The severity of peer bullying will be assessed using the 6-item severity subscale of the Social Contexts and Experiences measure (EU-GEI, 2014, Hubbard et al., 2015), which allows to compute categorical severity scores of not bullied, mild, moderate, severe, and extremely severe bullying.

Primary outcomes:
The primary outcome for the efficacy of the new EMI will be the level of self-esteem as measured with EMA and the Rosenberg Self-Esteem Scale.

Rosenberg Self-Esteem Scale (RSES): The RSES (Rosenberg, 1965) is a widely used measure to assess global self-esteem with good reliability and validity (Everaert et al., 2010, Schmitt and Allik, 2009). The RSES consists of 10 items rated on a 4-point Likert scale ranging from 'strongly agree' to 'strongly disagree'.

Momentary self-esteem: Ecological Momentary Assessment (EMA) will be used to assess momentary self-esteem in daily life using 4 items (Thewissen et al., 2008, Thewissen et al., 2011) following the protocol from previous EMA studies (Myin-Germeys et al., 2011, Myin-Germeys et al., 2009, Reininghaus et al., 2016b, Reininghaus et al., 2016c). The EMA will be administered through the smartphone-based PsyMate® App for a period of 6 consecutive days. On each day, participants will be asked 10 times per day to complete an EMA, which will be scheduled at random within set blocks of time. During the 6-day EMA period, participants will be contacted by phone to offer advice about any potential questions about the EMA and assess their adherence to instructions. At the end of the 6-day EMA period, participants will be asked to return the device and complete a short debriefing questionnaire.

Secondary outcomes:
Resilience: Resilience will be measured with EMA and operationalized as less intense negative affect in response to momentary stress as well as an attenuated (or absent) decrease in positive affect in response to stress in daily life (Myin-Germeys et al., 2009, Reininghaus et al., 2016b, Reininghaus et al., 2016c).
Psychological distress: Psychological distress will be measured with the Kessler Psychological Distress Scale (K10), which is widely used and well-validated in youth (Kessler et al., 2002, Kwan and Rickwood, 2015).
Emotional well-being: We will assess emotional well-being using the Positive and Negative Affect Scale (Watson et al., 1988) as well as a 5-item EMA measure for assessing negative affect and a 4-item EMA measure of positive affect (Myin-Germeys et al., 2001, Reininghaus et al., 2016b, Reininghaus et al., 2016c).
Functioning: The Social and Occupational Functioning Assessment Scale (SOFAS) (Goldman et al., 1992) will be used as a well-validated measure of functioning in youth (Kwan and Rickwood, 2015).
Subjective quality of life: Subjective quality of life will be measured with the World Health Organisation Quality of Life Instrument-Brief (group, 1998, Kwan and Rickwood, 2015).
General psychopathology: The revised Symptom Checklist (SCL-90-R) will be used as a reliable and valid measure to assess general psychopathology in youth (Derogatis, 1977, Kwan and Rickwood, 2015).
Incidence of clinical symptoms: We will use the Brief Psychiatric Rating Scale (BPRS) (Lukoff et al., 1986, Overall et al., 1967) will be used as a validated interviewer measure to assess incidence of clinical symptoms of psychopathology in youth (Kwan and Rickwood, 2015).
Saliva samples: Saliva samples will be collected for targeted DNA methylation analyses of predictors for (and correlates of) training success; targets for these epigenetic analyses will be derived from recent studies on epigenetic profiles related to trauma (Klengel and Binder, 2015, Rutten et al., 2014b) and to treatment response. DNA methylation levels will be quantified per locus by pyrosequening in amplicons within NR3C1 and SLCA4, resulting in continuous variables on DNA methylation levels. The rationale for collecting saliva samples is based on several recent findings that exposure to 'environmental' factors (such as childhood trauma or behavioural interventions) can have persistent influences on the accessibility and expression of genes (through so-called epigenetic mechanisms) and may be linked to dynamic changes in mental phenotypes. The complementary epigenetic analyses on DNA methylation profiles in NR3C1 (i.e. glucocorticoid receptor 1) and SLCA4 (i.e. the serotonin transporter), which have previously been linked to treatment response (Yehuda et al., 2013) and/or psychological trauma (Vinkers et al., 2015) will provide a heuristic explanation linking psychological and behavioural data with direct biological data, thereby fulfilling the biopsychosocial working model of dynamic changes in mental health, and may possibly reflect a biomarker predicting treatment response (Yehuda et al., 2013). The data will be collected in a secure, coded and confidential way and cannot be traced back by to the research participants for other reasons than the proposed research.

Other study parameters:
Other study parameters will include basic socio-demographic characteristics and other potential confounders (including age, sex, and alcohol/substance use) that may be associated with primary and secondary outcome measures. The CSRI (Chisholm et al., 2000) will be used to record patients' contacts with mental health services, monitor variation in delivery of TAU and model economic outcomes. For all participants in the intervention group, progress toward the effective use of SELFIE exercises will be rated using a 10-point visual analogue scale by the staff therapist (Bond and Lader, 1974). In addition, audio recordings of face-to-face sessions and EMA data of the SELFIE intervention (i.e., adherence to tasks) will be used to assess fidelity to the treatment protocol.

Statistical analysis
The primary analysis will be an intention to treat analysis or an available case analysis following intention to treat principles, with data from all participants entered into the analysis. We will make every effort to assess all participants at post-treatment and follow-up and use multiple imputation for handling missing data where appropriate. Linear mixed modelling in Stata 13 will be used to compare outcomes between experimental and control condition at post-treatment and follow-up. The primary outcome of self-esteem measured at post-treatment and follow-up will be entered as the dependent variable and self-esteem measured at baseline and condition as independent variables. In a next step, time and a time × condition interaction term will be added as independent variables to the previous model. Within-subject clustering of repeated measures will be taken into account by including a subject varying random intercept. The analysis of secondary outcomes will follow the same steps. Mulitlevel moderated mediation analysis of EMA data will be used to investigate whether effects of condition on resilience, emotional well-being, functioning, quality of life, and general psychopathology are mediated via self-esteem (Klippel et al., 2017).

Epigenetic analysis
We will focus on methylation of genes suggested by previous studies to be involved in the response to trauma and/or treatment: the serotonin transporter gene (SLC6A4) and the glucocorticoid receptor gene (NR3C1). The SLC6A4 and NR3C1 amplicons that will be used have been described in previous studies (Perroud et al., 2013, Roberts et al., 2014, Yehuda et al., 2013). For each saliva sample, DNA will be extracted from mononuclear cells and 1 µg of DNA will be subjected to bisulfite treatment using the MethylDetector kit (Active Motif, Carlsbad, CA, USA). For the conversion reaction, we will use a long incubation protocol (Hompes et al., 2014). Bisulfite-treated DNA samples will be subjected to polymerase chain reaction for amplification of the SLC6A4 and NR3C1 amplicons, using primers designed using the PyroMark Assay design software (Qiagen, Manchester, UK). Amplicons will be sequenced using two sequencing primers to maximize coverage using the Pyrosequencing Pyromark Q24 system (Qiagen). Only CpG units with a success rate of 60% or more will be included for further analyses.

Economic evaluation
The CSRI (Chisholm et al., 2000) will be used as a measure for calculating cost effectiveness by combining service use data with available unit cost information comparing between conditions, at post-intervention and follow-up: a) proportion of participants using each service based on the CSRI; b) mean cost of each service, and c) mean total cost. Further, cost-consequences analysis will be carried out by combining costs with primary outcome measures using cost effectiveness planes.

Expertise, voorgaande activiteiten en producten / Expertise, prior activities and products

The Department of Psychiatry and Psychology in the European Graduate School of Neuroscience at Maastricht University Medical Centre is a world leader in the area of multivariate clinical diagnostics using multivariate tools, including novel approaches in the area of diagnostic symptom networks in relation to neural networks and has developed a unique and leading multidisciplinary collaboration in the areas of diagnostics, epidemiology, imaging, neuropsychiatry, genetics and basic neuroscience in order to study the etiology, diagnosis and treatment of mental disorders. The group has specific expertise in ecological momentary assessment and intervention technologies. The interdisciplinary research team consists of leading academics and a youth representative (de Roos), with expertise across all the necessary disciplines including in ecological momentary interventions, youth mental health, randomized controlled trials, linking epigenetic profiles with treatment and outcomes, and smart device engineering. Specifically, Dr. Reininghaus will provide expertise in ecological momentary interventions targeting psychological mechanisms in randomized controlled trials as well as the use of ecological momentary assessment in youth, high risk and clinical populations. Prof. van Amelsvoort will provide expertise in youth mental health and transition psychiatry. With the chair in transitional psychiatry in the Netherlands, she initiated a discussion and movement towards youth mental health reform. She obtained a VENI grant (2006, ZonMw), followed by a VIDI grant (2012, ZonMw) and (co)-authored over 130 papers. Prof. van der Gaag will provide expertise in psychological and ecological momentary interventions and the delivery and conduct of randomized controlled trials. Prof. Rutten will provide expertise in linking epidemiology (exposure to trauma), molecular epigenetic profiles, treatment and outcomes and neurobiological understanding of mental disorder. He will oversee the analysis on epigenetic mechanisms underlying changes in primary and secondary outcomes. Prof. Lindauer is child and adolescent psychiatrist, family therapist at the Centre for Trauma and Family at De Bascule, Amsterdam, and Head of the Department of Child and Adolescent Psychiatry at the AMC, and will provide expertise in screening and assessment of psychological trauma, the effects of treating trauma-related disorders (including the biological aspects of treatment), and the use of epigenetic methods for understanding the effects of psychological trauma, predicting treatment response, and measuring the effects of treatment. Prof. Schruers is a psychiatrist and clinical neuroscientist, but also a licensed behavioural therapist. He will provide expertise in mechanisms and treatment of affective disorders and specifically on the underlying (psychological as well as biological) mechanisms of psychological treatments. Dr. Nieman is clinical psychologist and principal investigator at the AMC Department of Psychiatry and will provide expertise on early detection and treatment of psychiatric symptoms in young people. Dr. Tuijthof will provide expertise in optimising the acceptability and usability of the SELFIE App using principles of smart device engineering. Gerard de Roos (Ixta Noa) will provide advice on all aspects of the study as a youth representative and, together with a group of youth from Ixta Noa, closely involve youth from Ixta Noa in month 1-6 of the project in order to adopt the design of the PsyMate® App and optimise its acceptability and usability for youth in an iterative process of repeated assessment of acceptability and usability by youth, followed by programming and adopting the design until optimal levels of acceptability and usability are achieved.
The research team has extensive experience from previous studies with recruitment, assessment and delivery of interventions in youth. Our recruitment strategies are directly based on this work in youth and on conservative recruitment targets derived from consulting with local mental health services. We will also draw on routine screening, assessment, and genetic sampling by local partners, which will allow considerable resource savings.

Publicaties / Publications

de Koning, M.B., Bloemen, O.J., van Amelsvoort, T.A., Becker, H.E., Nieman, D.H., van der Gaag, M., Linszen, D.H. (2009). Early intervention in patients at ultra high risk of psychosis: benefits and risks. Acta Psychiatr Scand 119, 426-442.
Diehle, J., Schmitt, K., Daams. J., Boer, F., & Lindauer, R.J.L. (2014). Effects of psychotherapy on trauma-related cognitions in posttraumatic stress disorder: a meta-analysis. J Traumatic Stress 27, 257-264.
Diehle, J., Opmeer, B.C., Mannarino, A., Boer, F., & Lindauer, R.J.L. (2014). Trauma-focused Cognitive Behavioral Therapy or Eye Movement Desensitization and Reprocessing - what works in children with posttraumatic stress symptoms? A randomized controlled trial. Eur J Child Adolesc Psychiatry 24(2), 1-10.
Goossens, L., Sunaert, S., Peeters, R., Griez, E. J. & Schruers, K. R. (2007). Amygdala hyperfunction in phobic fear normalizes after exposure. Biol Psychiatry 62, 1119-25.
Jonkman, C.S., Verlinden, E., Bolle, E.A., Boer, F., & Lindauer, R.J.L. (2013). Traumatic stress symptomatology in children after child maltreatment and single traumatic events: Clinical implications. J Traumatic Stress, 26, 225-232.
Kooij, van der I.W., Nieuwendam, J., Moerman, G., Boer, F., Lindauer, R.J.L., Roopnarine, J., & Graafsma, T. (in press). Perceptions among Creoles and Maroons on corporal punishment in Suriname. Child Abuse Rev.
Klippel, A., Myin-Germeys, I., Chavez-Baldini, U., Preacher, K.J., Kempton, M., Valmaggia, L., Calem, M., So, S., Beards, S., Hubbard, K., Gayer-Anderson, C., Wichers, M., McGuire, P., Murray, R.M., Garety, P., van Os J., Wykes, T., Morgan, C. & Reininghaus, U. (2017). Modelling the interplay between psychological processes and adverse, stressful contexts and experiences in pathways to psychosis: an experience sampling study. Schizophr Bull 43, 302-315.
Knuts, I., Esquivel, G., Kenis, G., Overbeek, T., Leibold, N., Goossens, L. & Schruers, K. (2014). Therapygenetics: 5-HTTLPR genotype predicts the response to exposure therapy for agoraphobia. Eur Neuropsychopharmacol 24, 1222-8.
Knuts, I. J., Esquivel, G., Overbeek, T. & Schruers, K. R. (2015). Intensive behavioral therapy for agoraphobia. J Affect Disord 174, 19-22.
Lange, I., Goossens, L., Leibold, N., Vervliet, B., Sunaert, S., Peeters, R., van Amelsvoort, T. & Schruers, K. (2016). Brain and Behavior Changes following Exposure Therapy Predict Outcome at 8-Year Follow-Up. Psychother Psychosom 85, 238-40.
Kraan, T., Velthorst, E., Smit, F., de Haan, L. & van der Gaag, M. (2015b). Trauma and recent life events in individuals at ultra high risk for psychosis: review and meta-analysis. Schizophr Res 161, 143-9
Kraan, T. C., Ising, H. K., Fokkema, M., Velthorst, E., van den Berg, D. P., Kerkhoven, M., Veling, W., Smit, F., Linszen, D. H., Nieman, D. H., Wunderink, L., Boonstra, N., Klaassen, R. M., Dragt, S., Rietdijk, J., de Haan, L. & van der Gaag, M. (2017). The effect of childhood adversity on 4-year outcome in individuals at ultra high risk for psychosis in the Dutch Early Detection Intervention Evaluation (EDIE-NL) Trial. Psychiatry Res 247, 55-62.
Lindauer, R.J.L., Brilleslijper-Kater, S.N., Diehle, J., Verlinden, E., Teeuw, A.H., Middeldorp, C.M., Tuinebreijer, W., Bosschaart, T.F., Duin, E. van, & Verhoeff, A. (2014). The Amsterdam Sexual Abuse Case (ASAC)-study in day care centers: Longitudinal effects of sexual abuse on infants and very young children and their parents, and the consequences of the persistence of abusive images on the internet. BMC Psychiatry 14, 295 doi:10.1186/s12888-014-0295-7.
Myin-Germeys, I., Klippel, A., Steinhart, H. & Reininghaus, U. (2016). Ecological momentary interventions in psychiatry. Curr Op Psychiatry 29, 258-263.
Nieman, D.H. & McGorry P.M. (2015). Detection and treatment of At Risk Mental State for developing a first psychosis: Making up the balance. Lancet Psychiatry 2, 825-34.
Nieman, D.H. (2015). New treatments for psychotic disorders. Lancet Psychiatry 2, 282-283
Nieman, D.H., Ruhrmann, S., Dragt, S., Soen, F., van Tricht, M.J., Koelman, J.H., Bour, L.J., Velthorst, E., Becker, H.E., Weiser, M., Linszen, D.H., de Haan, L. (2014). Psychosis prediction: stratification of risk estimation with information-processing and Premorbid Functioning Variables. Schizophr Bull 40, 1482-90.
Pishva, E., Kenis, G., Lesch, K. P., Prickaerts, J., Steinbusch, H., van Os, J. & Rutten, B. P. (2012). Epigenetic epidemiology in psychiatry. Translational Neuroscience 3, 196-212.
Reininghaus, U., Depp, C.A. & Myin-Germeys, I. (2016). Ecological interventionist causal models in psychosis: targeting psychological mechanisms in daily life. Schizophr Bull 42, 264-269.
Reininghaus, U., Gayer-Anderson, C., Valmaggia, L., Kempton, M.J., Calem, M., Onyejiaka, A., Hubbard, K., Dazzan, P., Beards, S., Fisher, H.L., Mills, J.G., McGuire, P., Craig, T.K., Garety, P., van Os, J., Murray, R.M., Wykes, T., Myin-Germeys, I. & Morgan, C. (2016). Psychological processes underlying the association between childhood trauma and psychosis in daily life: an experience sampling study. Psychol Med 46, 2799-2813.
Reininghaus, U., Morgan, C., Fearon, P., Hutchinson, G., Morgan, K., Dazzan, P., Boydell, J., Kirkbride, J.B., Doody, G.A., Jones, P.B., Murray, R.M. & Craig. T.K. (2014). Modelling the interplay between childhood and adult adversity in pathways to psychosis: initial evidence from the AESOP study. Psychol Med 44, 407-419.
Rutten, B. P., Hammels, C., Geschwind, N., Menne-Lothmann, C., Pishva, E., Schruers, K., van den Hove, D., Kenis, G., van Os, J. & Wichers, M. (2013). Resilience in mental health: linking psychological and neurobiological perspectives. Acta Psychiatr Scand 128, 3-20.
Rutten, B. P. & Mill, J. (2009). Epigenetic mediation of environmental influences in major psychotic disorders. Schizophr Bull 35, 1045-56.
Sommer, I.E., Bearden, C.E., van Dellen, E., Breetvelt, E.J., Duijff, S.N., Maijer, K., van Amelsvoort, T., de Haan, L., Gur, R.E., Arango, C., Díaz-Caneja, C.M., Vinkers, C.H., Vorstman, J.A. (2016). Early interventions in risk groups for schizophrenia: what are we waiting for? NPJ Schizophr 9, 16003.
Steinhart, H., Myin-Germeys, I. & Reininghaus, U. (in press). Translating treatment of mental health problems to daily life: a guide to the development of ecological momentary interventions. in Novel uses of experience sampling in mental health research. Edited by Palmier-Claus J, Haddock G, Varese F. London, UK, Routledge.
van Amelsvoort, T. (2014). Bridging the gap. Tijdschr Psychiatr 56, 638-9
Verlinden, E., Meijel, E.P.M. van, Opmeer, B.C., Beer, R., Roos, C. de, Bicanic, I.A.E., Lamers-Winkelman, F., Olff, M., Boer, F., & Lindauer, R.J.L. (2014). Characteristics of the Children's Revised Impact of Event Scale in a Clinically Referred Dutch Sample. J Traumatic Stress 27, 328-334.
van Amelsvoort, T.A., Van Den Eede, F., Goethals, K., van Marle, H.J., Beekman, A.J. (2014). Structural changes in DSM-5:

the beginning of a transformation? Tijdschr Psychiatr 56, 152-156.
van Houtem-Solberg, D.M., Chatrou, E.W., Werrij, M.Q., van Amelsvoort, T.A. (2015). Youth F-ACT: mapping the problems of a special population. Tijdschr Psychiatr 57, 892-896.
Verlinden, E., Schippers, M., Meijel, E.P.M. van, Beer, R., Opmeer, B.C., Olff, M., Boer, F., &
Zantvoord, J.B., Diehle, J., & Lindauer, R.J.L. (2013). Using neurobiological measures to predict and assess treatment outcome of psychotherapy in posttraumatic stress disorder: systematic review. Psychoth Psychosom 82, 142-151.

Referenties / References

Bakker, D., Kazantzis, N., Rickwood, D. & Rickard, N. (2016). JMIR Ment Health 3, e7.
Banyard, V. L., Williams, L. M. & Siegel, J. A. (2001). J Trauma Stress 14, 697-715.
Bernstein, D. P. & Fink, L. (1998). Childhood Trauma Questionnaire. The Psychological Corporation: San Antonio (Texas).
Brown, G. W., Craig, T. K., Harris, T. O. & Handley, R. V. (2008). J Affect Disord 110, 115-25.
Chen, L. P., Murad, M. H., Paras, M. L., et al. (2010). Mayo Clin Proc 85, 618-29.
Chisholm, D., Knapp, M. R., Knudsen, H. C., et al. (2000). Br J Psychiatry Suppl, s28-33.
Copeland, W., Shanahan, L., Costello, E. J. & Angold, A. (2011). J Am Acad Child Adolesc Psychiatry 50, 252-61.
Derogatis, L. R. (1977). SCL-90-R. Johns Hopkins: Baltimore, MD.
Dinesen, B., Nonnecke, B., Lindeman, D., et al. (2016). J Med Internet Res 18, e53.
EU-GEI. (2014). Schizophr Bull 40, 729-36.
Everaert, J., Koster, E. H. W., Schacht, R. & De Raedt, R. (2010). Gedragsther 43, 307-317.
Fisher, H., Morgan, C., Dazzan, P., et al. (2009). Br J Psychiatry 194, 319-25.
Fisher, H. L., Appiah-Kusi, E. & Grant, C. (2012). Psychiatry Res 196, 323-4.
Fisher, H. L., Schreier, A., Zammit, S., et al. (2013). Schizophr Bull 39, 1045-55.
Frissen, A., Lieverse, R., Drukker, M., et al. (2015). Soc Psychiatry Psychiatr Epidemiol 50, 1481-8.
Garety, P. A., Bebbington, P., Fowler, D., et al. (2007). Psychol Med 37, 1377-91.
Goldman, H. H., Skodol, A. E. & Lave, T. R. (1992). Am J Psychiatry 149, 1148-56.
Granholm, E., Ben-Zeev, D., Link, P. C., et al. (2012). Schizophr Bull 38, 414-25.
The WHOQOL Group (1998). Psychol Med 28, 551-8.
Harhay, M. O. & King, C. H. (2012). Lancet 379, 27-8.
Heron, K. E. & Smyth, J. M. (2010). Br J Health Psychol 15, 1-39.
Hompes, T., Izzi, B., Gellens, E., et al. (2014). J Psychiatr Res 56, 165-167.
Hotopf, M. (2002). Adv Psychiatr Treatment 2002, 326-333.
Hsieh, H. F. & Shannon, S. E. (2005). Qual Health Res 15, 1277-88.
Hubbard, K., Beards, S., Gayer-Anderson, C.,…& Reininghaus, U. (2015). Schizophr Bull 41, S142.
Kamsner, S. & McCabe, M. P. (2000). J Interpers Violence 15, 1243–61.
Kessler, R. C., Andrews, G., Colpe, L. J., et al. (2002). Psychol Med 32, 959-76.
Kessler, R. C., Berglund, P., Demler, O., et al. (2005). Arch Gen Psychiatry 62, 593-602.
Kessler, R. C., McLaughlin, K. A., Green, J. G., et al. (2010). Br J Psychiatry 197, 378-85.
Kim-Cohen, J., Caspi, A., Moffitt, T. E., et al. (2003). Arch Gen Psychiatry 60, 709-17.
Kirkbride, J. B. & Jones, P. B. (2011). Schizophr Bull 37, 262-71.
Klengel, T. & Binder, E. B. (2015). Neuron 86, 1343-57.
Klippel, A., Myin-Germeys, I., Chavez-Baldini, U.,…& Reininghaus, U. (2017). Schizophr Bull 43, 302-315.
Kraan, T., van Dam, D. S., Velthorst, E.,... van der Gaag, M. & de Haan, L. (2015a). Schizophr Res 169, 193-8.
Kraan, T., Velthorst, E., Smit, F., de Haan, L. & van der Gaag, M. (2015b). Schizophr Res 161, 143-9.
Kraan, T. C., Ising, H. K., Fokkema, M.,… van der Gaag, M. (2017). Psychiatry Res 247, 55-62.
Kramer, I., Simons, C. J., Hartmann, J. A., et al. (2014). World Psychiatry 13, 68-77.
Kwan, B. & Rickwood, D. J. (2015). BMC Psychiatry 15, 279.
Lange, I., Goossens, L., Michielse, S., et al. (2016). Biological Psychiatry 79, S125.
Lukoff, D., Liberman, R. P. & Nuechterlein, K. H. (1986). Schizophr Bull 12, 578-602.
Malla, A., Iyer, S., McGorry, P., et al. (2016). Soc Psychiatry Psychiatr Epidemiol 51, 319-326.
Mangalore, R. & Knapp, M. (2007). J Ment Health Policy Econ 10, 23-41.
Mannarino, A. P. & Cohen, J. A. (1996). J Interpers Violence 11, 162–80.
Matheson, S. L., Shepherd, A. M., Pinchbeck, R. M., et al. (2013). Psychol Med 43, 225-38.
McCrone, P., Dhanasiri, S., Patel, A., et al. (2008). Paying the price. London.
McGorry, P., Bates, T. & Birchwood, M. (2013). Br J Psychiatry Suppl 54, s30-5.
McGowan, P. O., Sasaki, A., D'Alessio, A. C., et al. (2009). Nature Neuroscience 12, 342-348.
Morgan, C., Charalambides, M., Hutchinson, G. & Murray, R. M. (2010). Schizophr Bull 36, 655-64.
Murthi, M. & Espelage, D. L. (2005). Child Abuse Negl 29, 1215-31.
Myin-Germeys, I., Birchwood, M. & Kwapil, T. (2011). Schizophr Bull 37, 244-7.
Myin-Germeys, I., Klippel, A., Steinhart, H. & Reininghaus, U. (2016). Curr Opin Psychiatry 29, 258-63.
Myin-Germeys, I., Oorschot, M., Collip, D., et al. (2009). Psychol Med 39, 1533-47.
Myin-Germeys, I., van Os, J., Schwartz, J. E., et al. (2001). Arch Gen Psychiatry 58, 1137-44.
Nielsen, J. & Molich, R. (1990). Heuristic evaluation of user interfaces. Proc ACM CHI'90 Conf: Seattle, WA.
Overall, J. E., Hollister, L. E. & Pichot, P. (1967). Arch Gen Psychiatry 16, 146-51.
Overholser, J. C., Adams, D. M., Lehnert, K. L. & Brinkman, D. C. (1995). J Am Acad Child Adolesc Psychiatry 34, 919-28.
Pereda, N., Guilera, G., Forns, M. & Gomez-Benito, J. (2009). Clin Psychol Rev 29, 328-38.
Perroud, N., Salzmann, A., Prada, P., et al. (2013). Transl Psychiatry 3, e207.
Pishva, E., Kenis, G., Lesch, K. P., et al. (2012). Translational Neuroscience 3, 196-212.
Pishva, E., Kenis, G., van den Hove, D., et al. (2014). Soc Psychiatry Psychiatr Epidemiol 49, 337-48.
Reininghaus, U., Depp, C. A. & Myin-Germeys, I. (2016a). Schizophr Bull 42, 264-9.

Reininghaus, U., Gayer-Anderson, C., Valmaggia, L., et al. (2016b). Psychol Med 46, 2799-813.
Reininghaus, U., Kempton, M. J., Valmaggia, L., et al. (2016c). Schizophr Bull 42, 712-22.
Reininghaus, U. & Morgan, C. (2014). Soc Psychiatry Psychiatr Epidemiol 49, 1-2.
Reininghaus, U., Morgan, C., Fearon, P., et al. (2014). Psychol Med 44, 407-19.
Roberts, S., Lester, K. J., Hudson, J. L., et al. (2014). Transl Psychiatry 4, e444.
Rojas, S. V. & Gagnon, M. P. (2008). Telemed J E Health 14, 896-904.
Rosenberg, M. (1965). Society and the adolescent self-image. Princeton University Press: Princeton, NJ.
Rutten, B. P. & Mill, J. (2009). Schizophr Bull 35, 1045-56.
Rutten, B. P., Vermetten, E., Vinkers, C., et al. (2014a). Biol Psychiatry 75, 216S-216S.
Scher, C. D., Stein, M. B., Asmundson, G. J., et al. (2001). J Trauma Stress 14, 843-57.
Schmitt, P. & Allik, J. (2009). J Pers Soc Psychol 89, 623-642.
Staring, A. B., van den Berg, D. P., Cath, D. C., et al. (2016). Behav Res Ther 82, 11-20.
Steinhart, H., Myin-Germeys, I. & Reininghaus, U. (in press). Translating treatment of mental health problems to daily life: a guide to the development of ecological momentary interventions. In Novel uses of experience sampling in mental health research (ed. J. Palmier-Claus, G. Haddock and F. Varese). Routledge: London, UK.
The Schizophrenia Commission (2012). The abandoned illness. London.
Thewissen, V., Bentall, R. P., Lecomte, T., et al. (2008). J Abnorm Psychol 117, 143-53.
Thewissen, V., Bentall, R. P., Oorschot, M., et al. (2011). Br J Clin Psychol 50, 178-95.
Thompson, J. L., Kelly, M., Kimhy, D., et al. (2009). Schizophr Res 108, 176-81.
Turecki, G. & Meaney, M. J. (2016). Biol Psychiatry 79, 87-96.
Varese, F., Smeets, F., Drukker, M., et al. (2012). Schizophr Bull 38, 661-71.
Vialou, V., Feng, J., Robison, A. J. & Nestler, E. J. (2013). Ann Rev Pharmacol Toxicol 53, 59-87.
Vinkers, C. H., Kalafateli, A. L., Rutten, B. P., et al. (2015). Epigenomics 7, 593-608.
Watson, D., Clark, L. A. & Tellegen, A. (1988). J Pers Soc Psychol 54, 1063-70.
Wright, K. D., Asmundson, G. J., McCreary, D. R., et al. (2001). Depress Anxiety 13, 179-83.
Yehuda, R., Daskalakis, N. P., Desarnaud, F., et al. (2013). Front Psychiatry 4, 118.
Zeigler-Hill, V. (2011). J Contemp Psychoth 41, 157-164.

Efficacy:

Hypothesis 1 (H1): It is hypothesized that, compared with the control condition (TAU), self-esteem (primary outcome) will, on average, be lower in the experimental condition (SELFIE + TAU; i.e., post-intervention, 6-month follow-up) than in the control condition, while controlling for self-esteem and center at baseline.

To test the effect of SELFIE + treatment as usual (TAU) compared to TAU only on the primary outcome variable (self-esteem measured with the RSES), we will use a linear regression model with the primary outcome of self-esteem at post-intervention and 6-month follow-up entered as the dependent variable and self-esteem measured at baseline (grand-mean centered), condition (SELFIE + TAU vs. TAU), time (as a two-level factor) and center (as a four-level factor) as independent variables, in line with the intention-to-treat principle. Residuals within subjects will be allowed to be correlated with a completely unstructured variance-covariance matrix to take within-subject clustering of repeated measures into account. The model will be fitted using robust REML.

$\text{Self-esteem}_{ij} = \beta_0 + \beta_1\ \text{time}_{ij} + \beta_2\ \text{condition}_{ij} + \beta_3\ \text{baseline self-esteem}_j + \beta_4\ \text{condition (experimental vs. control condition)}_j + \beta_5\ \text{center (Zuid-Holland vs. Noord-Holland)}_j + \beta_6\ \text{center (Limburg vs. Noord-Holland)}_j + \beta_7\ \text{center (general population vs. Noord-Holland)} + u_{0j} + e_{ij}$

Self-esteem is the outcome for person *j* at time point *i*, with i=0 (post-intervention), 1 (6-month follow-up). u_{0j} is the random intercept and e_{ij} is the residual error. β_2 indicates the main effect of condition on self-esteem parameterized in order to reflect the difference between the two conditions at the two follow-up points (i.e., post-intervention and 6-month follow-up), which will be tested (at $\alpha = .05$) by a Wald-type test with df=1. This tests the joint null hypothesis (that there is no difference at both follow-up time points) against the alternative hypothesis (that there is, on average, a difference across the two follow-up time points). The alternative hypothesis allows for deviations in either direction at both time points. The main effect β_2 is expected to have a positive point estimate, which indicates that, on average, levels of self-esteem are higher in the experimental condition compared to the control condition across post-intervention and 6-month follow-up.

Hypothesis 2 (H2): It is hypothesized that, compared with the control condition (TAU), positive self-esteem, positive schematic beliefs of self, emotional well-being, functioning, quality of life, and momentary self-esteem, resilience, and positive affect (ESM outcomes), will, on average be higher and negative self-esteem, negative schematic beliefs of self, psychological distress, general psychopathology, clinical symptoms and momentary negative affect (ESM outcome)

will be lower in the experimental condition (SELFIE + TAU); post-intervention and 6-month follow-up (secondary outcomes), while controlling for respective secondary outcome scores and center at baseline.

To test the effect of SELFIE + TAU compared to TAU only on the non-ESM secondary outcomes (i.e., positive and negative self-esteem, positive and negative schematic beliefs of self, emotional well-being, psychological distress, general psychopathology, clinical symptoms, functioning, and subjective quality of life), we will use separate (multilevel) linear regression models with the secondary outcomes at post-intervention and 6-month follow-up entered as dependent variables and positive and negative self-esteem, positive and negative schematic beliefs of self, emotional well-being, psychological distress, general psychopathology, clinical symptoms, functioning, and subjective quality of life at baseline, condition (SELFIE + TAU vs. TAU), time (as a two-level factor), and center (as a four-level factor), as independent variables, in line with the intention-to-treat principle. Residuals within subjects will be allowed to be correlated with a completely unstructured variance-covariance matrix to take within-subject clustering of repeated measures into account. The model will be fitted using robust REML.

Secondary $\text{outcomes}_{ij} = \beta_0 + \beta_1\ \text{time}_{ij} + \beta_2\ \text{condition}_{ij} + \beta_3$ baseline secondary $\text{outcomes}_j + \beta_4$ condition (experimental vs. control $\text{condition})_j + \beta_5$ center (Zuid-Holland vs. $\text{Noord-Holland})_j$ + β_6 center (Limburg vs. $\text{Noord-Holland})_j + \beta_7$ center (general population vs. $\text{Noord-Holland})_j$ + $u_{0j} + e_{ij}$

Positive and negative self-esteem, positive and negative schematic beliefs of self, emotional well-being, psychological distress, general psychopathology, clinical symptoms, functioning, and subjective quality of life are the secondary outcomes for person *j* at time point *i*, with i=0 (post-intervention), 1 (6-month follow-up). u_{0j} is the random intercept and e_{ij} is the residual error. β_2 reflects the main effect of condition on the secondary outcome variables parameterized in order to reflect the difference between the two conditions at the two follow-up points (i.e., post-intervention and 6-month follow-up), which will be tested (at $\alpha = .05$) by a Wald-type test with df=1. This tests the joint null hypothesis (that there is no difference at both follow-up time points) against the alternative hypothesis (that there is (on average) a difference across the two follow-up time points). The alternative hypothesis allows for deviations in either direction at both time points. The main effect of β_2 is expected to have a negative point estimate for negative self-esteem, negative schematic beliefs, psychological distress, and general psychopathology and clinical symptoms, indicating that, compared to the control condition, there will be, on average, lower scores on these outcomes in the experimental condition across

post-intervention and 6-month follow-up. By contrast, the main effect of β_2 is expected to have a positive point estimate for positive self-esteem, positive schematic beliefs, emotional well-being, functioning and quality of life, indicating that, compared to the control condition, there will be, on average, higher scores on these outcomes in the experimental condition across post-intervention and 6-month follow-up.

Second, in order to test the effect of SELFIE + TAU compared to TAU only on the secondary ESM outcomes (i.e., momentary self-esteem, resilience, negative affect and positive affect), we will fit separate (multilevel) linear regression models with the secondary ESM outcomes (eight assessments per day on six consecutive days each) at post-intervention and 6-month follow-up entered as dependent variables and the secondary ESM outcomes (person-mean centered) at baseline, condition (SELFIE + TAU vs. TAU), time (as a two-level factor), center (as a four-level factor), a secondary ESM outcome at baseline × time interaction, and a condition × time interaction as independent variables, in line with the intention-to-treat principle.

A two-level model with time points (post-intervention, 6-month follow-up; level 1, *i*) nested within subjects (level 2, *j*) will be estimated. For subject and time point, a random intercept will be included, while we will include random slopes for time (level 2; only with an error term, no predictors) and secondary ESM outcomes (level 2; only with an error term, no predictors). The variance-covariance matrix of these effects will be set to unstructured.

Additionally, we assume that the within-subject residuals are autocorrelated (i.e., a marginal model instead of adding an explicit level) for the ESM observations at each time point (i.e., post-intervention and 6-month follow-up), and therefore, they will be modelled with an autoregressive (AR) structure (of the exponential type). This allows the models to account for unequally spaced time values, indicating “hours since midnight of the first ESM day” will be used as time indicator in the models, as this takes the longer delay between the last beep of the day and the first beep of the morning in account.

Again, the model will be fitted using robust REML. Should the model not converge, we will first check whether this is due to a lack of variance for any of the random effects and, if so, remove the respective random effect; if this does not solve the non-convergence, we will fit a simpler model without a random slope for time; or finally, we might have to fit a simpler model with an autoregressive AR1 structure.

The formula for the model (for each separate secondary ESM outcome) including the indication that the variables are measured multiple times within each time point (index *k*; addressed via residual structure, see above) is formulated as:

Secondary ESM outcomes$_{ij}$ = β_0 + β_1 time$_{ij}$ + β_2 condition$_j$ β_3 baseline secondary ESM outcomes$_j$ + β_4 condition (experimental vs. control condition)$_j$ + β_5 center (Zuid-Holland vs. Noord-Holland)$_j$ + β_6 center (Limburg vs. Noord-Holland)$_j$ + β_7 center (general population vs. Noord-Holland)$_j$+ β_8 baseline secondary ESM outcomes$_j$ × time$_{ij}$ + β_9 condition$_j$ × time$_{ij}$ + β_{10} baseline secondary ESM outcomes$_{kij}$ × time$_{ij}$ + β_{11} baseline secondary ESM outcomes$_{kij}$ × condition$_j$ + β_{12} baseline secondary ESM outcomes$_{kij}$ × time$_{ij}$ × condition$_j$ + υ_j + u_{ij} + e_{ijk}

For each separate secondary ESM outcome, we will be looking at the effect of the secondary ESM outcome × time × condition interaction on the secondary ESM outcome observation *k* and person *j* at time point *i*. Estimates for the time-specific contrasts are expected to have negative point estimates for negative affect, indicating that these associations will be lower in the experimental condition compared with the control condition at post-intervention and/or 6-month follow-up. The estimates for momentary self-esteem, and positive affect are expected to have positive point estimates, indicating that these associations will be higher in the experimental condition compared to the control condition at post-intervention and/or 6-month follow-up. The estimated relationships will be plotted.

For momentary resilience, multilevel analyses will be carried out with negative affect / positive affect as the dependent variable and time_since as a predictor. We will account for effects of subsequent unpleasant events by adding the covariate subsequent stress (0=no subsequent unpleasant event, 1= subsequent unpleasant events).
Based on these models, linear contrasts will be used to compare the estimated average level of negative affect/ positive affect at the baseline beep (t_{-1}) with the estimated average negative affect / positive affect at t_0, t_1, t_2. In line with (30), we will count the number of beeps following the event where negative affect / positive affect significantly differed from the baseline beep, which will indicate the recovery period. Next, we will add time, condition, center and the condition*time*time_since interaction as predictors to the model to investigate differences in recovery across the time points. Pairwise comparisons will be used to further investigate differences between conditions using the lincom command.

Negative $affect_{kij}$ = β_0 + β_1 $time_{ij}$ + β_2 condition (experimental vs. $control)_j$ + β_3 $time\_since_{kij}$ + β_4 time_since at $baseline_j$ + β_5 subsequent $stress_j$ + β_6 center (Zuid-Holland vs. Noord-$Holland)_j$ + β_7 center (Limburg vs. Noord-$Holland)_j$ + β_8 center (general population vs. Noord-$Holland)_j$ + β_9 time_since at $baseline_j$ × $time_{ij}$ + β_{10} $condition_j$ × $time_{ij}$ + β_{11} $time\_since_{kij}$ × $time_{ij}$ + β_{12} $time\_since_{kij}$ × $condition_j$ + β_{13} $time\_since_{kij}$ × $time_{ij}$ × $condition_j$ + u_j + e_{ijk}

Hypothesis 3 (H3): It is hypothesized that, compared with the control condition (TAU), self-esteem, positive self-esteem, positive schematic beliefs of self, emotional well-being, functioning, quality of life, and momentary self-esteem, positive affect, resilience, will, on average be higher and negative self-esteem, negative schematic beliefs of self, psychological distress, general psychopathology, and clinical symptoms and negative affect (EMS outcome) will be lower in the experimental condition (SELFIE + TAU) at 18-month and 24-month follow-up, while controlling for respective secondary outcome scores and center at baseline.

To test whether the effect of SELFIE + TAU compared to TAU only on the primary (self-esteem) and secondary (momentary self-esteem, positive and negative schematic beliefs of self, resilience, emotional well-being, general psychopathology, clinical symptoms, functioning, and quality of life) outcomes hold at 18-month and 24-month follow up, we will fit separate linear regression models. The primary and secondary outcomes at 18-month and 24-month follow-up will be entered as dependent variables, while the independent variables in the models will be time (five-factor level), center (four-factor level), an outcome at baseline × time interaction, and a condition (two-level factor) × time interaction. The within subject clustering of repeated measures will be taken onto account by adding a level-2 random intercept and the models' level-1 residuals will be allowed to correlate with a completely unstructured error variance-covariance matrix. The models will be fitted using robust REML. The models for ESM- and non-ESM outcomes, and the interpretation of the models are comparable to those described for hypotheses 1 and 2.
