# Supplementary material for: Transdiagnostic Ecological Momentary Intervention for Improving Self-Esteem in Youth Exposed to Childhood Adversity: The SELFIE Randomized Clinical Trial
Source: JAMA Psychiatry. 2023 Nov 29;81(3):227–39. doi: 10.1001/jamapsychiatry.2023.4590 (PMC10687716; doi:10.1001/jamapsychiatry.2023.4590)
Supplement: Supplement 2. — eTable 1. Eligibility Criteria eMethods 1. SELFIE Intervention Description eTable 2. Overview of SELFIE Intervention Procedure eMethods 2. Measures eMethods 3. Statistical Analysis eTable 3. Diagnoses at Baseline eTable 4. Correlations Among Primary and Secondary Outcomes at Baseline eTable 5. Percentiles and Range of Baseline Characteristics of the Intention-to-Treat Sample eTable 6. Compliance With, Fidelity to, and Acceptance of the SELFIE Intervention eMethods 4. Sensitivity Analysis on Primary Outcome at Postintervention and Follow-Up Using Multiple Imputation eFigure. Response Trajectories in the Primary Outcome (RSES Self-Esteem) eAppendix 1. SELFIE in the Context of Previous and Future EMI Research eAppendix 2. Targeting Self-Esteem in Ecological Interventionist Causal Models and Transdiagnostic Intervention Research eReferences [file jamapsychiatry-e234590-s002.pdf]

Supplementary Online Content

Reininghaus U, Daemen M, Postma MR, et al. Transdiagnostic ecological momentary intervention for improving self-esteem in youth exposed to childhood adversity: the SELFIE randomized clinical trial. *JAMA Psychiatry*. Published online November 29, 2023. doi:10.1001/jamapsychiatry.2023.4590

eTable 1. Eligibility Criteria

eMethods 1. SELFIE Intervention Description

eTable 2. Overview of SELFIE Intervention Procedure

eMethods 2. Measures

eMethods 3. Statistical Analysis

eTable 3. Diagnoses at Baseline

eTable 4. Correlations Among Primary and Secondary Outcomes at Baseline

eTable 5. Percentiles and Range of Baseline Characteristics of the Intention-to-Treat Sample

eTable 6. Compliance With, Fidelity to, and Acceptance of the SELFIE Intervention

eMethods 4. Sensitivity Analysis on Primary Outcome at Postintervention and Follow-Up Using Multiple Imputation

eFigure. Response Trajectories in the Primary Outcome (RSES Self-Esteem)

eAppendix 1 SELFIE in Context of Previous and Future EMI Research

eAppendix 2 Targeting Self-Esteem in Ecological Interventionist Causal Models and Transdiagnostic Intervention Research

eReferences

This supplementary material has been provided by the authors to give readers additional information about their work.

**eTable 1.** Eligibility criteria
Inclusion criteria
1) Aged between 12 and 26 years old.
2) Adversity:<br>a. Childhood trauma:<br>Prior exposure to at least one form of childhood trauma defined as moderate or severe physical (score ≥ 10), sexual (score ≥ 8) and/or emotional (score ≥ 13) abuse, emotional (score ≥ 15), and/or physical (score ≥ 10) neglect, according to established severity categories of the Childhood Trauma Questionnaire (CTQ)[1-3], and/or<br>b. Peer bullying:<br>Exposure to moderate or severe peer bullying, measured with the Retrospective Bullying Questionnaire (RBQ) (score of frequency of bullying in one or more ways "sometimes" or more often and/or classified the experience as "quite serious" or "extremely serious")[4], and/or<br>c. Parental conflict:<br>A score of moderate or severe parental conflict, measured with the Childhood Experiences of Care and Abuse Questionnaire (CECA.Q) section Parental Conflict (frequency score of "regularly" or "often" and/or a severity score of "serious" or "violence")[5].
3) Self-esteem below average (measured with the Rosenberg Self-Esteem Scale (RSES) using the cut-off (RSES total score <26) applied in previous research on psychological interventions for reducing self-esteem[6, 7].
4) Willingness to participate in the SELFIE intervention.
5) Ability to give written informed consent.
6) Parental consent for minors.
Exclusion criteria
1) Insufficient command of Dutch
2) Psychiatric symptoms due to an organic cause

© 2023 Reininghaus U et al. *JAMA Psychiatry.*

eMethods 1. SELFIE intervention description

The SELFIE intervention was delivered by trained psychologists within a 6-week period in addition to treatment as usual (TAU) to individuals allocated to the experimental condition. The one-day training for SELFIE therapists (mental health professionals working at clinical sites) consisted of delivering background information on the SELFIE trial (i.e. study protocol, theoretical framework of self-esteem), making oneself familiar with the intervention guideline, practicing exercises, and learning how to apply the smartphone application when delivering the intervention. When delivering the intervention, regular inter- and supervision was offered by a clinical psychologist.

The intervention consisted of three face-to-face sessions (each for around 60 minutes), delivered by SELFIE therapists on a fortnightly basis, , three email contacts with SELFIE therapists (again, delivered every other week as shown in eTable 2), and an EMI administered through a smartphone-based app (i.e., the PsyMate® app) for adaptive real-time and real-world transfer of intervention components tailored to person, moment, and context, delivered over the 6-week intervention period. Face-to-face sessions and emails introduced principles and techniques to be continued by the participant by use of the EMI in the week thereafter. Specifically, the EMI translated the training from face-to-face sessions into individuals' daily lives based on three types of delivery schemes (i.e., enhancing tasks, consolidating tasks, interactive tasks (see below for further detail)). Contact with SELFIE therapists offered an opportunity to reflect on progress and discuss any issues participants faced with the EMI.

eTable 2. Overview of SELFIE intervention procedure

Week 1	Face-to-face session
Week 2	Email contact
Week 3	Face-to-face session
Week 4	Email contact
Week 5	Face-to-face session
Week 6	Email contact
After completing the SELFIE intervention	Short face-to-face closing session

Due to the COVID-19 pandemic, most of the face-to-face sessions were offered through a secure and encrypted video conferencing system. The intervention was based on principles of EMIs[8-15], and a guided self-help approach using principles of cognitive-behavioral therapy (CBT), aimed at modifying cognitive bias (selective perception and selective memory) inherent to negative self-esteem (by actively searching for positive information and other experiences), building a competitive positive self-esteem, and developing and practicing a new behavioral repertoire guided by therapists using modeling and

© 2023 Reininghaus U et al. *JAMA Psychiatry.*

shaping as additional important therapeutic techniques[16, 17]. Delivering the intervention in individuals' daily lives, and enabling youth to benefit from this intervention in a given moment and context, when most needed (e.g. in moments of low self-esteem) was the key goal of the 6-week SELFIE intervention. Therefore, in the first introductory session, participants either received a study smartphone with the app already installed or were asked to install it on their smartphone by the SELFIE therapist, who explained the SELFIE intervention in detail and asked the participant to complete intervention exercises on the app to address the self-selected goals the participant wanted to work on in the 6-week intervention period. The app offered participants 'enhancing', 'consolidating', and 'interactive' tasks (see Table 2 in main manuscript).

In enhancing tasks, new intervention components were introduced and practiced, some of which were modified and extended over the intervention period. Consolidating tasks asked participants to practice previously learned components of enhancing tasks on a daily basis. For these tasks, participants were reminded by the app between 1-3 times per day (varying by intervention week). During the intervention period, Ecological Momentary Assessment (EMA), a structured dairy technique, was used to assess momentary self-esteem, affect, and pleasantness of activities and events, six times a day, on days 3, 4, and 5 on each of the six intervention weeks using a time-based design with stratified random sampling (i.e., with EMAs scheduled at random within set blocks of time) to allow for interactive tasks. Interactive tasks were provided based on their EMA ratings of (positive and negative) affect, momentary self-esteem, and pleasantness of activities and events. The threshold for triggering interactive tasks was either high (operationalized as a rating of 4 or higher on a 7-point Likert scale ranging from 1 to 7) or low (operationalized as a rating of 3 or lower on a 7-point Likert scale ranging from 1 to 7) positive affect, momentary self-esteem, and/or pleasantness of activities, on items of established and validated EMA measures (with a rating of 4 or higher being used as the triggering threshold, as this reflects the midpoint on the 7-point Likert scale, with any score equal to or above indexing high ratings on this scale). When participants provided high ratings, they were directed to the exercise 'positive datalog' and asked to add more successes (week 1) and/or positive qualities (week 3) to their datalog. When participants provided low ratings (a score of 3 or lower), they were directed to the exercise 'positive datalog' to show them all the successes they had previously entered. In the fifth week of the intervention period, participants were additionally asked whether they had received criticism since the last beep. If they answered affirmative, they were directed to the exercise 'a critical look at criticism' to offer them a technique to deal with criticism.

Participants could discontinue the intervention at any time upon request without negative consequences. After completion of the intervention period, or at drop-out, participants deleted the app on their phones and had no longer access to the app.

© 2023 Reininghaus U et al. *JAMA Psychiatry.*

eMethods 2. Measures

Screening

Inclusion criteria on adversity were assessed using the Childhood Trauma Questionnaire (short version) (CTQ [1-3]), the Retrospective Bully Questionnaire (RBQ [4]), and the Childhood Experiences of Care and Abuse Questionnaire (CECA.Q [5]), section Parental Conflict. Furthermore, the Rosenberg Self-Esteem Scale (RSES [6, 7]) was assessed (see eTable 1 in Supplement 1).

The Dutch version of the 25-item CTQ enquires about five types of childhood trauma (physical, emotional and sexual abuse, and physical and emotional neglect). All five types of trauma are covered with five items, rated on a 5-point Likert scale (1=Never true; 5=Very often true). Cut-off scores for including participants on childhood trauma were as follows: a score of ≥ 10 on physical abuse, a score of ≥ 8 on sexual abuse, a score of ≥ 13 on emotional abuse, a score of ≥ 15 on emotional neglect, and/or a score of ≥ 10 on physical neglect, according to established severity categories of the CTQ [1-3]).

The RBQ consisted of nine items that assess physical, verbal and indirect bullying in primary and secondary schools. Participants were included in the study when they reported the frequency of bullying in one or more ways (as "sometimes" or more often and/or classified the experience as "quite serious" or "extremely serious") on the RBQ [4].

The CECA.Q, section Parental Conflict is an interview-based measure to assess frequency and severity of parental conflict [5]. Cut-off scores for this study were frequency scores of "regularly" or "often" and/or a severity score of "serious" or "violence".

Moreover, the inclusion criterion of low self-esteem was operationalized as a total score of <26 on the RSES[6, 7, 16-18] based on previous research on psychological interventions for reducing self-esteem[6, 7]. The RSES is a widely used 10-item self-report measure to assess global self-esteem with good reliability and validity[6, 19]. All ten items were rated on a 4-point Likert scale (1=strongly agree; 4=strongly disagree). The Cronbach's alpha of the RSES at screening was .78.

Data on basic socio-demographic characteristics were collected on age, gender, level of education, and employment status, and self-ascribed ethnicity and race (i.e., Moroccan, Turkish, Surinamese, other) to allow for a basic epidemiological characterization of the sample based on evidence on the basic risk factor epidemiology of mental disorders.

Compliance with, fidelity to, and acceptance of the SELFIE intervention

Fidelity to the SELFIE intervention protocol was based on a) SELFIE therapists' personal ratings of core components delivered in the sessions, and b) independent ratings of core components delivered in the sessions of a selection (n=72 sessions, 31,7% of all delivered sessions) of audio recordings of the face-to-face sessions (using maximum variation sampling to include audio recordings of all three

© 2023 Reininghaus U et al. *JAMA Psychiatry.*

sessions, and all SELFIE therapists). In addition, user experience and acceptance of, as well as satisfaction with the SELFIE intervention was assessed using a self-report measure that included 31 items rated on a 7-point scale, ranging from a rating of 1 referring to 'not at all', via a rating of 4 referring to 'moderate', to a rating of 7 referring to 'very much'. Also, the Working Alliance Inventory (WAI)[20] was completed by the participant (WAI-P) and the SELFIE therapist providing the SELFIE intervention (WAI-T) including 12 items rated on a 5-point Likert scale. We used the mean score of the 12 items for patient and therapist ratings to obtain measures of participant- and therapist-rated working alliance.

Outcomes

Blinded assessors collected data on outcomes before randomization (at 'baseline'), at the end of the 6-week intervention period ('post-intervention'), and at 6-month follow-up ('follow-up'). Participants aged 16 years or older were compensated for their time and travel expenses were fully reimbursed.

Primary outcome

The primary outcome was global self-esteem, measured with the RSES [18], which is a widely used measure to assess global self-esteem with good reliability and validity [6, 19]. The RSES consists of ten items rated on a 4-point Likert scale ranging from 'strongly agree' to 'strongly disagree'. The level of global self-esteem, operationalized as the total score of the RSES, was compared between the experimental and the control condition at post-intervention and 6-month follow-up. The reliability estimate of the RSES total score in the current study was acceptable (Cronbach's α = .78).

Secondary outcomes

Secondary outcomes were positive/negative self-esteem, schematic beliefs of self, emotional well-being, psychological distress, general psychopathology, clinical symptoms, subjective quality of life, and functioning (assessed with non-EMA measures) as well as momentary self-esteem, momentary affect, and momentary resilience (assessed with EMA measures), which all reflect distinct outcomes of interest as an intervention target of the SELFIE intervention to allow for detecting signals of efficacy and, thereby, inform further optimization of the intervention and subsequent research.

Non-EMA measures

Positive/negative self-esteem. Positive and negative self-esteem were measured with the Self-Esteem Rating Scale (SERS), which is a 20-item rating scale to assess these two dimensions of self-esteem separately with good reliability and validity [21]. All items were rated on a 7-point Likert scale. The reliability estimate of the 10 items to measure positive self-esteem was .86, while for the 10 items to

© 2023 Reininghaus U et al. *JAMA Psychiatry.*

measure negative self-esteem the Chronbach's α was .84.

Schematic beliefs of self The Brief Core Schema Scale (BCSS) was used as an established 12-item measure of positive and negative schematic beliefs of self [22]. The internal consistency of the positive- and negative-self schema scales were .76 and .71, respectively.

Emotional well-being. Emotional well-being was assessed using the Positive and Negative Affect Scale (PANAS) [23], consisting of 20 items. The internal consistency of the positive and negative affect scales was .78 and .90, respectively.

Psychological distress. Psychological distress was measured with the Kessler Psychological Distress Scale (K10), which is a widely used and well-validated in youth [24, 25]. The K10 is a 10-item questionnaire to assess psychological distress in the last month on a scale from 1='never' to 5='always'. We used the sum score for this measure. The internal consistency of the K10 was Cronbach's α =.86.

General psychopathology. The revised Symptom Checklist (SCL-90-R) was used as a reliable and valid measure to assess general psychopathology in youth [25, 26]. The measure consisted of 90 items, all rated on a 5-point scale. The total sum score of the SCL-90-R was used. The Alpha value for internal consistency of this measure was .97.

Clinical symptoms. We used the 24-item version of the Brief Psychiatric Rating Scale (BPRS) [27, 28] as a validated interviewer measure to assess clinical symptoms of psychopathology in youth [25]. All items were rated on a 7-point scale. The intensity of psychopathological symptoms was indicated by the BPRS total score (range 24 to 168). We conducted five reliability meetings in order to assess the interrater reliability for the BPRS scores. The Intra-Class Correlation (ICC) coefficients ranged from .82 to .96.

Subjective quality of life. Subjective quality of life was measured with the World Health Organisation Quality of Life Instrument-Brief (WHOQOL-BREF) [25, 29]. Using this questionnaire, mean scores of four domains were measured (physical health, psychological health, social relationships and environment), with reliability estimates of the domains being .62, .73, .61 and .72, respectively.

Functioning. The Social and Occupational Functioning Assessment Scale (SOFAS) [30] and the Global Assessment of Functioning (GAF) scale [31] were used as a well-validated measure of functioning in youth [25]. For the analysis, we used the overall level of functioning rated by researchers on a scale of 0 to 100.

EMA measures

During baseline, post-intervention and at 6-month follow-up participants completed eight independent periods of Ecological Momentary Assessment (EMA) for six consecutive days (i.e., at baseline, post-intervention, and follow-up) using validated EMA measures[32-39]. Serious adverse events were monitored throughout the entire study period. Assessments were scheduled at random within set blocks of time. We used established and validated EMA measures to assess self-esteem, negative

© 2023 Reininghaus U et al. *JAMA Psychiatry.*

affect, positive affect, and resilience [32-34, 38-40]. EMA items on self-esteem, negative affect, and positive affect were all rated on a 7-point Likert-scale of 1='not at all' to 7='very much'.

Momentary self-esteem. Momentary self-esteem was assessed with the following four items [38, 39]: "I like myself", "I am ashamed of myself", "I am satisfied with myself", and "I doubt myself". The internal consistency of these items was α = .90

Momentary affect. Momentary affect was measured using a 5-item EMA measure for assessing negative affect and a 4-item EMA measure of positive affect [32-34]. Positive affect was measured with the following items: "I feel happy", "I feel satisfied", "I feel relaxed", "I feel cheerful", and "I can accept my feelings", while negative affect was assessed using the items: "I feel anxious", "I feel down", "I feel uneasy", "I feel lonely", "I feel insecure". Alpha values for internal consistency were .94 and .92, respectively.

Momentary resilience. Momentary resilience was assessed with the EMA and was operationalized as negative affective recovery in response to momentary stress as well as positive affective recovery in response to event-related stress in daily life[33-35, 37, 40].

© 2023 Reininghaus U et al. *JAMA Psychiatry.*

eMethods 3. Statistical analysis

Based on a pre-specified and published statistical analysis plan (SAP)[41], statistical analyses were performed in Stata version 16[42], while being blinded to random allocation to experimental and control condition. The following hypotheses were tested: First, we hypothesized that, compared with the control condition (care as usual (CAU)), self-esteem will, on average, be higher in the experimental condition (SELFIE+CAU) than in the control condition across post-intervention and 6-month follow-up (primary outcome). Second, we hypothesized that, compared with the control condition (CAU), momentary self-esteem, positive self-esteem, positive schematic beliefs of self, emotional well-being, momentary resilience, momentary positive affect, functioning, and subjective quality of life will, on average, be higher and negative self-esteem, negative schematic beliefs of self, momentary negative affect, psychological distress, general psychopathology, and clinical symptoms (secondary outcomes) will be lower in the experimental condition (SELFIE + CAU). The trial was powered to detect an effect size (standardized mean difference (SMD)) of 0.3 (experimental vs. control condition), i.e., a previously reported difference in the RSES self-esteem score that can be considered as clinically relevant for low-level interventions (adopting a conservative strategy)[43]. Sample size calculation using power simulation in the R environment indicated that a sample size of 130 participants (65 per condition) is sufficient to test our primary hypothesis that levels of self-esteem are, on average, higher in the experimental than control condition across post-intervention and follow-up with a power of 0.87 (primary hypothesis) when testing at 2-tailed alpha = 0.05 using linear mixed modeling. To allow for 12.5% attrition at 6-month follow-up (25% at 24-month follow-up), we aimed to recruit n=174 participants at baseline. In line with the intention-to-treat principle, all data of participants were used in the analysis, including data of participants who dropped out from the intervention, and those with low adherence. We fitted the model using Robust Restricted Maximum Likelihood (robust REML[44]) in Stata 16[42], which assumes that data is missing at random[45-48]. Therefore, potential bias due to attrition over the study period, differences between regions/centres, or levels of self-esteem at baseline were mitigated by the model. In addition, we assessed potential bias due to missing outcomes in descriptive analyses of baseline characteristics stratified by missing data for condition and the primary outcome. In a sensitivity analysis using multiple imputation in Stata 16, we examined the main effect of condition on self-esteem measured with the RSES, including all n=174, with missing data imputed for baseline variables as well as RSES self-esteem at post-intervention and follow-up as the primary outcome (see eMethods 4).

To test the effect of SELFIE + care as usual (CAU) compared to CAU only on the primary outcome variable (self-esteem measured with the RSES), we fitted a mixed effects regression model with the primary outcome of self-esteem at post-intervention and 6-month follow-up entered as the dependent

© 2023 Reininghaus U et al. *JAMA Psychiatry.*

variable and self-esteem measured at baseline (grand-mean centered), condition (SELFIE + CAU vs. CAU), time (as a two-level factor) and center (as a four-level factor) as independent variables, in line with the intention-to-treat principle. Residuals within subjects were allowed to be correlated with a completely unstructured variance-covariance matrix to take within-subject clustering of repeated measures into account. The model was fitted using robust Restricted Maximum Likelihood (REML) estimation, assuming data is missing at random. The main effect of condition (β_2) on self-esteem was parameterized in order to reflect the difference between the two conditions at the two follow-up points (i.e., post-intervention and 6-month follow-up), which was tested (at $\alpha = .05$) by a Wald-type test with df = 1. This tested the joint null hypothesis (that there is no difference at both follow-up time points) against the alternative hypothesis (that there is, on average, a difference across the two follow-up time points). The alternative hypothesis allowed for deviations in either direction at both time points. We computed p-values to index statistical significance at $\alpha < .05$.

To test the effect of SELFIE + CAU compared to CAU only on the non-EMA secondary outcomes (i.e., positive and negative self-esteem, positive and negative schematic beliefs of self, emotional well-being, psychological distress, general psychopathology, clinical symptoms, functioning, and subjective quality of life), we used separate (multilevel) linear regression models with the secondary outcomes at post-intervention and 6-month follow-up entered as dependent variables and positive and negative self-esteem, positive and negative schematic beliefs of self, emotional well-being, psychological distress, general psychopathology, clinical symptoms, functioning, and subjective quality of life at baseline, condition (SELFIE + CAU vs. CAU), time (as a two-level factor), and center (as a four-level factor), as independent variables, in line with the intention-to-treat principle. Residuals within subjects were allowed to be correlated with a completely unstructured variance-covariance matrix to take within-subject clustering of repeated measures into account. The model was fitted using robust REML. The main effect of condition (β_2) on the secondary outcome variables was parameterized in order to reflect the difference between the two conditions at the two follow-up points (i.e., post-intervention and 6-month follow-up), which were tested (at $\alpha = .05$) by a Wald-type test with df=1. This tested the joint null hypothesis (that there is no difference at both follow-up time points).

Further, in order to test the effect of SELFIE + CAU compared to CAU only on the secondary EMA outcomes (i.e., momentary self-esteem, resilience, negative affect and positive affect), we fitted separate (multilevel) mixed effects regression models with the secondary EMA outcomes (eight assessments per day on six consecutive days each) at post-intervention and 6-month follow-up entered as dependent variables and the secondary EMA outcomes (person-mean centered) at baseline,

condition (SELFIE + CAU vs. CAU), time (as a two-level factor), center (as a four-level factor), a secondary EMA outcome at baseline × time interaction, and a condition × time interaction as independent variables, in line with the intention-to-treat principle. A two-level model with time points (post-intervention, 6-month follow-up; level 1, *i*) nested within subjects (level 2, *j*) was estimated. For subject and time point, a random intercept was included, while we included random slopes for time (level 2; only with an error term, no predictors) and secondary EMA outcomes (level 2; only with an error term, no predictors). The variance-covariance matrix of these effects was set to unstructured. Additionally, we assumed that the within-subject residuals were autocorrelated at each time point for the EMA observations. The model was fitted using robust REML.

For all above analyses, Cohen's d-type effect sizes and 95% Confidence Intervals (CIs) were constructed for the primary and secondary outcomes in order to provide an estimate of the magnitude of differences between conditions[49]. A d-type effect size was obtained by dividing the model coefficient for the difference between the two conditions by the square root of the total variance. All d-type effect sizes reported in the tables of EMA outcomes are based on the total variance estimated in the mixed model, which is deemed more appropriate in cases where there is (potential for) large heterogeneity between level-2 units (here: participants; random intercept and residual error terms[49-51]. For determining effect sizes in more complex models including a random slope for time, we also used the total model-based variance observed in the EMA measures at post-intervention[52], i.e., we fitted the full model to obtain the variance estimate and then divided the coefficient for the between-group difference by the square root of this total variance. The effect sizes for these therefore represent the effect size (in case of interaction effects: the effect size due to a particular difference between two sub-groups) with reference to the total variation observed in the EMA measures at any point during the post-intervention or follow-up phases.

© 2023 Reininghaus U et al. *JAMA Psychiatry.*

eTable 3. Diagnoses at baseline

	Full sample (174)	Experimental condition (85)	Control condition (89)
Mood disorder	51 (29.31%)	27 (31.76%)	24 (26.97%)
Neurodevelopmental disorder	44 (25.29%)	19 (22.35%)	25 (28.09%)
Trauma and stressor-related disorder	36 (20.69%)	16 (18.82%)	20 (22.47%)
Anxiety disorder	32 (18.39%)	15 (17.64%)	17 (19.10%)
Personality disorder	28 (16.09%)	11 (12.94%)	17 (19.10%)
Eating disorder	17 (9.77%)	9 (10.59%)	8 (8.99%)
Relational problems	9 (5.17%)	3 (3.53%)	6 (6.74%)
Psychotic disorder	4 (2.30%)	2 (2.36%)	2 (2.25%)
Other	7 (4.02%)	2 (2.36%)	5 (5.62%)
No diagnosis	38 (21.84%)	20 (23.53%)	18 (20.22%)
Missing	15 (8.62%)	11 (12.94%)	4 (4.49%)

Note: cumulative frequencies (percentages) exceed the total sample size (100%) due to comorbidities

© 2023 Reininghaus U et al. *JAMA Psychiatry.*

eTable 4a. Correlations among primary and secondary outcomes at baseline

	RSES	SERS +	SERS -	BCSS POS. SELF	BCSS NEG. SELF	PANAS +	PANAS -	SCL-90-R	K10	BPRS	WHOQOL – PHYS.	WHOQOL – PSYCH.	WHOQOL – SOC.	WHOQOL – ENVIR.	SOFAS	GAF
**RSES**	1.0	-	-	-	-	-	-	-	-	-	-	-	-	-	-	-
**SERS +**	0.47**	1.0	-	-	-	-	-	-	-	-	-	-	-	-	-	-
**SERS -**	-0.59**	-0.27**	1.0	-	-	-	-	-	-	-	-	-	-	-	-	-
**BCSS POS. SELF**	0.54**	0.57**	-0.45**	1.0	-	-	-	-	-	-	-	-	-	-	-	-
**BCSS NEG. SELF**	-0.66**	-0.40**	0.63**	-0.43**	1.0	-	-	-	-	-	-	-	-	-	-	-
**PANAS +**	0.11	0.34**	-0.02	0.31**	-0.08	1.0	-	-	-	-	-	-	-	-	-	-
**PANAS -**	-0.38**	-0.14	0.53**	-0.24*	0.58**	0.05	1.0	-	-	-	-	-	-	-	-	-
**SCL-90-R**	-0.48**	-0.18*	0.59**	-0.32**	0.62**	0.11	0.65**	1.0	-	-	-	-	-	-	-	-
**K10**	0.47**	0.16*	-0.56**	0.31**	-0.52**	0.10	-0.54**	-0.76**	1.0	-	-	-	-	-	-	-
**BPRS**	-0.31**	-0.13	0.26**	-0.23**	0.33**	-0.10	0.37**	0.50**	-0.45**	1.0	-	-	-	-	-	-
**WHOQOL—PHYS.**	0.34**	0.15*	-0.31**	0.30**	-0.34**	0.04	-0.44**	-0.49**	0.46**	-0.37**	1.0	-	-	-	-	-
**WHOQOL – PSYCH.**	0.69**	0.42**	-0.64**	0.54**	-0.62**	0.28**	-0.44**	-0.57**	0.64**	-0.46**	0.43**	1.0	-	-	-	-
**WHOQOL – SOC.**	0.32**	0.35**	-0.39**	0.31**	-0.41**	0.08	-0.22*	-0.35**	0.20*	-0.11**	0.22*	0.35**	1.0	-	-	-
**WHOQOL – ENVIR.**	0.27**	0.14	-0.31**	0.25**	-0.35**	0.03	-0.34**	-0.43**	0.35**	-0.39**	0.50**	0.33**	0.36**	1.0	-	-
**SOFAS**	0.30**	0.22*	-0.20*	0.32**	-0.34**	0.03	-0.27**	-0.37**	0.30**	-0.66**	0.35**	0.40**	0.15*	0.40**	1.0	-
**GAF**	0.31**	0.19*	-0.24*	0.34**	-0.38**	0.04	-0.34**	-0.39**	0.35**	-0.68**	0.33**	0.43**	0.11	0.37**	0.84**	1.0

Note: * = p < 0.05, ** = p ≤ 0.001; RSES, global self-esteem measured with the Rosenberg Self-esteem Scale (RSES); SERS +, positive self-esteem measured with the Self-Esteem Rating Scale (SERS); SERS -, negative self-esteem measured with the SERS; BCSS pos. self, positive schematic beliefs of self, measured with the Brief Core Schema Scales (BCSS); BCSS neg. self, negative schematic beliefs of self measured with the BCSS; PANAS +, positive affect measured with the Positive and Negative Affect Scale (PANAS); PANAS -, negative affect measured with the PANAS; SCL-90-R, general psychopathology measured with the revised Symptom Checklist (SCL-90-R); K10, psychological distress measured with the Kessler Psychological Distress Scale (K10); BPRS, clinical symptoms measured with the Brief Psychiatric Rating Scale (BPRS); WHOQol – Phys., quality of life in the physical domain measured with the World Health Organisation Quality of Life Instrument-Brief (WHOQOL-BREF); WHOQol – Psych., quality of life in the psychological domain measured with the WHOQOL-BREF; WHOQol – Soc., quality of life in the social domain measured with the

© 2023 Reininghaus U et al. *JAMA Psychiatry.*

WHOQOL-BREF; WHOQol – Envir., quality of life in the environmental domain measured with the WHOQOL-BREF. SOFAS; functioning measured with the Social and Occupational Functioning Assessment Scale (SOFAS); GAF, Global Assessment of Functioning scale (GAF).

Table 4b. Correlations between EMA outcomes at baseline

	SELF-ESTEEM	NEGATIVE AFFECT	POSITIVE AFFECT
**SELF-ESTEEM**	1.0	-	-
**NEGATIVE AFFECT**	-0.84**	1.0	-
**POSITIVE AFFECT**	0.74**	-0.61**	1.0

Note. ** = p ≤ 0.001; Correlations were conducted using one average score per subject.

© 2023 Reininghaus U et al. *JAMA Psychiatry.*

eTable 5. Percentiles and range of baseline characteristics of the intention-to-treat sample.

	25th, 50th and 75th percentile	range
Age	19, 21, 23	12 – 25
Childhood trauma		
Emotional abuse	11, 14, 18	5 – 25
Physical abuse	5, 5, 8	5 – 22
Sexual abuse	5, 5, 12	5 – 25
Emotional neglect	11, 15, 18	5 – 25
Physical neglect	6, 8, 11	5 – 18
RSES	16, 19, 22	10 – 25
SERS		
Positive self-esteem	22, 30, 35	6 – 51
Negative self-esteem	34, 39, 46	7 – 60
BCSS		
Positive self	2, 5, 8	0 – 21
Negative self	5, 8, 13	0 – 24
Positive other	4, 7, 10	0 – 21
Negative other	2, 4, 8	0 – 20
PANAS Positive Affect	18, 21.5, 27	10 – 38
PANAS Negative Affect	15, 23, 29	10 – 45
K10 (total score)	23, 26.5, 32	10 – 47
SCL-90-R (total score)	188, 226, 267	110 – 381
BPRS (total score)	39, 43, 49	30 – 66
WHOQol-BREF		
Physical domain	19, 21, 24	12 – 30
Psychological domain	12, 14, 16	7 – 25
Social domain	7, 9, 11	3 – 15
Environmental domain	25, 28, 31	13 – 39
SOFAS	58, 65, 75	35 – 90
GAF	58, 65, 70	40 – 88

Note: RBQ, Retrospective Bullying Questionnaire; BPRS, Brief Psychiatric Rating Scale; K10, Kessler Psychological Distress Scale; SCL-90-R, Symptom Checklist 90-R; PANAS, Positive and Negative Affect Scale; GAF, Global Assessment of Functioning; SOFAS, Social and Occupational Functioning Assessment Scale; WHOQOL-BREF, World Health Organisation Quality of Life Instrument-Brief; SERS, Self-esteem Rating Scale; RSES, Rosenberg Self-esteem Scale; BCSS, Brief Core Schema Scale.

© 2023 Reininghaus U et al. *JAMA Psychiatry.*

eTable 6a. Compliance with, fidelity to, and acceptance of the SELFIE intervention.

		n
a) Session attendance in %		
Session 1		77 (90.59%)
Session 2		76 (89.41%)
Session 3		73 (85.88%)
b) Fidelity to intervention protocol		
Study therapist rating of core components delivered in %		
Session 1		65
Yes	95.66%	
Somewhat	3.92%	
No	0.14%	
Missing	0.28%	
Session 2		65
Yes	90.77%	
Somewhat	7.38%	
No	0.31%	
Missing	1.54%	
Session 3		63
Yes	86.67%	
Somewhat	8.25%	
No	0.32%	
Missing	4.76%	
Independent rating of core components delivered in %*		
Session 1		23
Yes	96.44%	
Somewhat	2.37%	
No	0.79%	
Missing	0.40%	
Session 2		25
Yes	97.60%	
Somewhat	1.60%	
No	0.80%	
Missing	0.00%	
Session 3		23
Yes	93.04%	
Somewhat	3.91%	
No	3.04%	
Missing	0.00%	
Number of EMI tasks completed per person, per week, mean (S.D.), range	13.28 (11.12), 1-82	
Number of ESM assessments completed per person, per week, mean (S.D.), range	4.72 (3.27), 1 - 18	
c) User experience, mean (S.D.)**		61
Did you experience a change in your self-esteem		
Positive change	4.80 (1.35)	
Negative change	1.92 (1.26)	
To what extent did the following elements contribute to a change in your self-esteem:		
Exercises via app	4.65 (1.57)	
Availability of exercises at all times	4.62 (1.44)	
Sessions with therapist	5.33 (1.39)	
Email contacts	3.95 (1.60)	
To what extent were the following exercises helpful in building your new self-esteem		
Formulating a new positive core belief	4.72 (1.30)	

© 2023 Reininghaus U et al. *JAMA Psychiatry.*

Positive data-log	5.62 (1.40)	
Positive quality list	5.23 (1.20)	
One minute exercise	4.42 (1.70)	
Development of new behavior patterns	4.82 (1.24)	
A critical look at criticism	5.03 (1.65)	
The minimum programme	4.53 (1.69)	
Writing a positive story about yourself	3.93 (1.72)	
Maintenance plan	4.14 (1.65)	
Was one of the following exercises harmful to the building of your new positive self-esteem		
Formulating a new positive core belief	1.52 (1.19)	
Positive data-log	1.46 (1.26)	
Positive quality list	1.53 (1.22)	
One minute exercise	1.51 (1.13)	
Development of new behavior patterns	1.47 (1.07)	
A critical look at criticism	1.44 (1.19)	
The minimum programme	1.41 (1.04)	
Writing a positive story about yourself	1.42 (1.03)	
Maintenance plan	1.25 (0.69)	
d) Satisfaction with SELFIE intervention, mean (S.D.)**		61
Was the SELFIE intervention useful for you?	5.23 (1.35)	
Were the homework exercises useful?	5.22 (1.34)	
Were the face-to-face-sessions useful?	5.42 (1.37)	
Was the guidance within the SELFIE intervention sufficient?	6.12 (0.96)	
Was the SELFIE intervention applicable to your symptoms?	5.13 (1.64)	
e) Acceptance of the SELFIE intervention, mean (S.D.)**		61
To what extent are you convinced of the effect of the SELFIE intervention?	5.03 (1,43)	
Did you experience this way of receiving help as pleasant?	5.53 (1.43)	
f) Working Alliance, mean (S.D.)***		
Working Alliance Inventory participant	4.07 (0.54)	69
Working Alliance Inventory Therapist	3.96 (0.39)	64

Note: n, number of participants; S.D., standard deviation, Missing values in user experience, satisfaction and acceptance questionnaire, n=16.
* 30% of all delivered intervention sessions have been rated by an independent rater through the audio recordings. The to be rated sessions were selected based on creating a sample with a variety of therapists who delivered the session as well as a variety of sessions (1, 2, or 3).
** rating on a scale from 1 to 7.
*** rating on a scale from 1 to 5.

© 2023 Reininghaus U et al. *JAMA Psychiatry.*

eTable 6b. Compliance with, fidelity to, and acceptance of the SELFIE intervention by using own vs. study smartphone‡

	Own smartphone	Study smartphone	t	P
a) User experience, mean (S.D.)				
Did you experience a change in your self-esteem				
Positive change	4.80 (1.32)	4.83 (1.72)	-0.06	0.955
Negative change	1.85 (1.16)	2.50 (1.97)	-1.20	0.235
To what extent did the following elements contribute to a change in your self-esteem:				
Exercises via app	4.63 (1.64)	4.83 (0.75)	-0.30	0.766
Availability of exercises at all times	4.78 (1.36)	3.17 (1.47)	2.74	0.008
Sessions with therapist	5.41 (1.37)	4.67 (1.51)	1.25	0.217
Email contacts	4.04 (1.64)	3.17 (0..98)	1.27	0.210
To what extent were the following exercises helpful in building your new self-esteem				
Formulating a new positive core belief	4.83 (1.27)	3.67 (1.21)	2.14	0.036
Positive data-log	5.70 (1.33)	4.83 (1.94)	1.45	0.151
Positive quality list	5.30 (1.19)	4.67 (1.21)	1.23	0.225
One minute exercise	4.46 (1.69)	4.00 (1.90)	0.63	0.532
Development of new behavior patterns	4.91 (1.26)	4.00 (0.63)	1.73	0.090
A critical look at criticism	5.04 (1.66)	5.00 (1.67)	0.05	0.958
The minimum programme	4.56 (1.69)	4.33 (1.86)	0.31	0.761
Writing a positive story about yourself	3.98 (1.70)	3.50 (2.07)	0.64	0.525
Maintenance plan	4.08 (1.67)	4.67 (1.51)	-0.82	0.414
Was one of the following exercises harmful to the building of your new positive self-esteem				
Formulating a new positive core belief	1.56 (1.24)	1.17 (0.41)	0.76	0.450
Positive data-log	1.45 (1.28)	1.50 (1.22)	-0.09	0.932
Positive quality list	1.53 (1.23)	1.50 (1.22)	0.05	0.958
One minute exercise	1.49 (1.09)	1.67 (1.63)	-0.36	0.722
Development of new behavior patterns	1.50 (1.11)	1.17 (0.41)	0.72	0.472
A critical look at criticism	1.47 (1.25)	1.17 (0.41)	0.59	0.557
The minimum programme	1.46 (1.09)	1.00 (0.00)	1.03	0.309
Writing a positive story about yourself	1.47 (1.08)	1.00 (0.00)	1.05	0.296
Maintenance plan	1.27 (0.72)	1.00 (0.00)	0.92	0.360
b) Satisfaction with SELFIE intervention, mean (S.D.)				
Was the SELFIE intervention useful for you?	5.27 (1.37)	4.83 (1.17)	0.75	0.454
Were the homework exercises useful?	5.26 (1.33)	4.83 (1.47)	0.73	0.465
Were the face-to-face-sessions useful?	5.50 (1.38)	4.67 (1.03)	1.43	0.159

© 2023 Reininghaus U et al. *JAMA Psychiatry.*

Was the guidance within the SELFIE intervention sufficient?	6.19 (0.95)	5.50 (0.84)	1.69	0.097
Was the SELFIE intervention applicable to your symptoms?	5.19 (1.65)	4.67 (1.63)	0.73	0.468
c) Acceptance of the SELFIE intervention, mean (S.D.)				
To what extent are you convinced of the effect of the SELFIE intervention?	5.06 (1.42)	4.83 (1.60)	0.36	0.721
Did you experience this way of receiving help as pleasant?	5.55 (1.49)	5.33 (0.82)	0.34	0.732

Note: S.D., standard deviation, all ratings on a scale from 1 to 7.

‡ Of n=85 participants randomized to the experimental condition, n=10 used a study smartphone, n=67 used their own smartphone, and n=8 did not start using a smartphone for SELFIE intervention delivery. Missing values in user experience, satisfaction and acceptance questionnaire, n=16.

© 2023 Reininghaus U et al. *JAMA Psychiatry.*

Table 6c Observer-rated secondary outcomes at post-intervention and 6-month follow-up, excluding assessments for which blinded assessors correctly guessed random allocation to experimental and control condition.‡

	Experimental condition			Control condition			Difference between conditions (at time)				
	Mean	SE	n	Mean	SE	n	Adj. B	95% CI	P-value	d-type effect size	95% CI
Clinical symptoms (BPRS)											
Main effect of condition							-0.86	-3.01 – 1.29	0.434	-0.14	-0.50 – 0.21
Time											
Post-intervention	41.48	0.99	62	43.71	1.16	73	-1.86	-4.31 – 0.59	0.137	-0.31	-0.72 – 0.10
Follow-up	40.44	0.99	55	41.39	1.06	67	-0.14	-2.41 – 2.70	0.912	0.02	-0.40 – 0.45
Functioning (SOFAS)											
Main effect of condition							-0.85	-3.55 – 1.84	0.535	-0.10	-0.43 – 0.23
Time											
Post-intervention	68.03	1.35	62	67.39	1.29	73	0.38	-2.95 – 3.71	0.825	0.05	-0.36 – 0.45
Follow-up	68.88	1.45	56	69.43	1.49	68	-2.08	-5.57 – 1.40	0.241	-0.26	-0.68 – 0.17
Functioning (GAF)											
Main effect of condition							0.18	-2.51 – 2.87	0.897	0.02	-0.32 – 0.36
Time											
Post-intervention	67.29	1.29	61	66.20	1.26	72	0.79	-2.48 – 4.07	0.635	0.10	0.31 – 0.52
Follow-up	67.88	1.30	56	67.00	1.54	68	-0.44	-3.85 – 2.97	0.802	-0.05	-0.48 – 0.38

Note: Adjusted for centered baseline values, region and group status; SE, Standard Error; N, number of participants; CI, confidence interval; BPRS, Brief Psychiatric Rating Scale; SOFAS, Social and Occupational Functioning Assessment Scale; GAF, Global Assessment of Functioning.

‡ Breaks in blinding occurred for 3 assessments, which were then re-assigned to, and completed by, other blind assessors. Blinded assessors correctly guessed allocation to experimental and control condition for 12 of 153 assessments at post-intervention (with n=8 correctly guessing allocation to experimental condition and n=4 correctly guessing allocation to control, condition), and for 4 of 140 assessments at 6-month follow-up (n=4 correctly guessing allocation to experimental condition and n=0 correctly guessing allocation to control condition).

© 2023 Reininghaus U et al. *JAMA Psychiatry.*

eMethods 4. Sensitivity analysis on primary outcome at post-intervention and 6-month follow-up using multiple imputation.

The analysis of the primary outcome was conducted assuming that data is missing at random given variables included as covariates in the model[48], as specified in our protocol paper[53] and preregistration on the open science framework[41]. While these were key variables that relate both to the substantive research question as well as to potential key drivers of discontinuation (e.g., baseline values of global self-esteem measured with the Rosenberg Self-Esteem Scale (RSES) and treatment allocation for selective responding and drop-out; centre for average differences in the target variables as well as site-effects on drop-out), these reflect only a limited number of variables, and on request of a peer-reviewer, we additionally report the results of an analysis based on multiply imputed data sets via chained equations. We opted for a chained equations approach as it allows a flexible specification of the imputation model across multiple time points. The imputations were conducted in Stata 16[42], and we imputed 20 data sets. The imputations were started with a fixed seed value (generated via random.org) and with 500 burn-in iterations. Continuous variables were predicted using linear regressions, dichotomous variables using logistic regressions, and the posterior estimates of the model parameters were estimated from bootstrap samples.

The imputation was performed separately for experimental and control condition[48], and used the following baseline variables (see details in Table 1): age, sex (dichotomised as male vs female), education (dichotomised as low vs middle and high), employment (dichotomised as active (employed and student) vs. non-active (unemployed)), ethnicity, study site, and medication representing participant mix, as well as the secondary outcome variables positive and negative self-esteem measured with the Self-Esteem Rating Scale (SERS); general psychopathology measured with the revised Symptom Checklist (SCL-90-R); the four subscales (physical, psychological, social, environment) for quality of life, assessed with the World Health Organisation Quality of Life Instrument-Brief (WHOQOL-BREF); the Positive and Negative Affect Scale (PANAS) subscales for positive and negative affect; clinical symptoms of psychopathology measured with the Brief Psychiatric Rating Scale (BPRS); the four subscales of positive and negative schematic beliefs of self and others measured with the Brief Core Schema Scale (BCSS); and functioning, assessed with both the Global Assessment of Functioning (GAF) scale and the Social and Occupational Functioning Assessment Scale (SOFAS).

The RSES entered the imputation in three places: it was used as a baseline variable, and the post-intervention and follow-up assessments as longitudinal endpoints. The baseline variables were all used to impute any baseline missing values (see Table 1, only very few missing values) as well as for the

© 2023 Reininghaus U et al. *JAMA Psychiatry.*

imputation of the RSES at post-intervention and follow-up. The post-intervention value of the RSES was also used to impute the follow-up assessment of the RSES. We decided on this set-up to represent the temporal order as well as since the overall sample size of the trial is small for models with many predictors and could easily result in models with more parameters than our sample size (especially in a by-group imputation). Additionally, as there are increasingly missing values on all variables across the three time points, but our main interest was the RSES, therefore using multiple variables from each time point could lead to increased prediction error.

We estimated the average difference in RSES self-esteem between conditions *across* post-intervention and follow-up as the primary outcome via employing a mixed model treating post-intervention and follow-up data as nested observations within individuals, with a separate random effect error term for time. This deviation from the main analysis model (see also eTable 7 below), which calculated predicted differences based on model-estimated coefficients, was necessary since the integration of multiply imputed data sets for these operations is not fully resolved at this point, and therefore to define a model that most-closely resembled the model test in a single model estimated from the multiply imputed data. Based on this model, the RSES was, on average, higher in the experimental than control condition across post-intervention and follow-up time points (B=2.35, 95% CI 1.13–3.57, p<0.001; original model: B=2.32, 95% CI 1.14–3.50, p<0.001).

eTable 7 further shows the results of re-estimating the main analysis model on the imputed data sets compared to the original parameter estimates. This sensitivity analysis showed broadly similar findings compared with our a priori planned main analysis for the primary outcome. While the 95% CIs were wider for the imputed data than for the original analysis, none of the estimated parameters showed a substantial shift. Specifically, the findings from the sensitivity analysis that the evaluated average difference in RSES levels between the two conditions (i.e., the primary outcome in the main analysis) was in the expected direction and the estimated coefficients were of similar size, indicating similar effect sizes. When inspecting findings at each time point separately, higher levels of global self-esteem in the experimental than control condition were observed at post-intervention and follow-up, with point estimates for between-condition differences being of similar size in the main and sensitivity analysis. Further, findings at post-intervention showed that levels of global self-esteem were higher in the experimental than control condition, with a very similar 95% CI that did not include 0 (B=2.74, 95% CI 1.34-4.14; adjusted estimate of main analysis B=2.83; 95% CI 1.46-4.20; see Table 3). The only difference between main and sensitivity analysis was that the analysis based on multiply imputed data showed a slightly smaller between-condition estimate at follow-up, with the 95% CI being wider and

potentially not different from "0" (1.58, 95% confidence interval -0.12 to 3.28; adjusted estimate of main analysis 1.81; 95% confidence interval 0.38 to 3.23; see Table 3).

eTable 7. Parameter estimates for the mixed model analysis for the primary outcome, left column based on n=174 with 348 observations after multiple imputation; right column presents estimates from originally estimated mixed model (see Table 3).

	Imputed Model<br>*B* (95% CI)	Originally estimated model<br>*B* (95% CI)
Intercept	22.58 (19.81 – 25.35)	22.02 (19.54 – 24.50)
Condition	2.82 (1.29 – 4.34)	2.83 (1.46 – 4.20)
Time	1.67 (0.29 – 3.04)	1.32 (0.29 – 2.35)
RSES baseline	0.83 (0.65 – 1.00)	0.88 (0.72 – 1.04)
Time × condition	-1.31 (-3.11 – 0.49)	-1.03 (-2.53 – 0.47)
Intercept<br>(Standard Deviation)	2.90 (2.24 – 3.74)	2.87 (2.33 – 3.53)
Residual<br>(Standard Deviation)	3.75 (3.09 – 4.55)	3.21 (2.84 – 3.62)

Note. *B* unstandardised coefficient. 95% CI, 95% confidence interval.

© 2023 Reininghaus U et al. *JAMA Psychiatry.*

eFigure. Response trajectories in the primary outcome (RSES self-esteem).

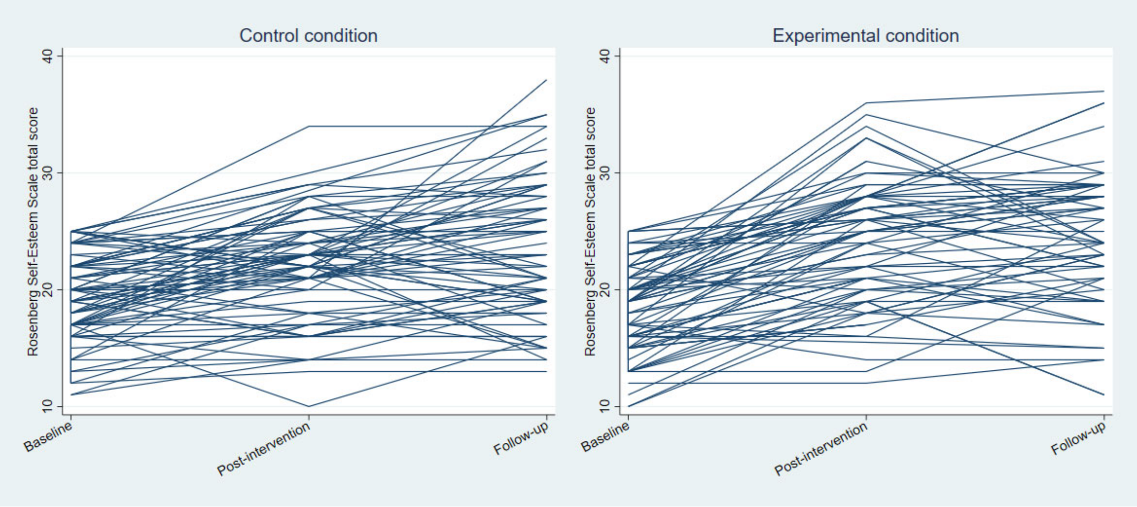

© 2023 Reininghaus U et al. *JAMA Psychiatry.*

eAppendix 1 SELFIE in context of previous and future EMI research

While digital mental health interventions, broadly defined, encompass telemedical, internet-based (eHealth), and mobile Health (mHealth)[54-56] interventions that have rapidly emerged over the past decade. Ecological Momentary Interventions (EMIs)[9, 57-60] such as SELFIE move beyond previous research by incorporating interactive and adaptive elements for real-world, real-time, interactive, adaptive, and personalized delivery of interventions that are centred around the dynamics of individuals' experience and behaviour, and their interaction with contextual factors in daily life[9, 59, 60]. The delivery of intervention components is informed by design principles of ecological momentary assessment (EMA), which enables real-time processing of fine-grained intensive longitudinal data collected via mobile devices[57, 61-63]. More specifically, EMI components are offered according to event-contingent, time-contingent, or hybrid EMA design principles and sampling strategies, building on long-standing methodological insights and a large body of evidence from over twenty years of EMA research[59, 61]. EMIs harness these design principles and sampling strategies to collect EMA data not only to enhance self-reflection, self-management or emotional awareness, but for delivering person-tailored interactive and adaptive intervention components when they are most needed in a given moment and context[9, 57]. To our knowledge, the current study reflects the first confirmatory RCT in the field of prevention and early intervention of mental disorders providing evidence for the efficacy of an EMI incorporating interactive and adaptive elements in this way and, as such, moves beyond previous trials of EMIs. It, thereby, provides important insights for the digital mental health field that interactive and adaptive EMI components may reflect promising active ingredients of mHealth interventions more generally. These may be optimized further based on machine learning algorithms to refine tailoring of intervention components and the timing of delivery to individuals’ current needs and context. For instance, there have been recent efforts to use machine learning techniques to assign interventions components of EMIs based on recurrent neural networks (RNNs[64, 65]), which can be harnessed to predict individuals’ proximal responses to interactive EMI components. While, overall, moderate to high user experience, satisfaction, and acceptance ratings were reported for the SELFIE intervention, this may be optimized further for email contacts and writing a positive story about oneself, both of which received, on average, slightly lower user experience ratings. Future research also needs to clarify why and how SELFIE works in our qualitative work conducted alongside the current trial as well as in subsequent trials using an active control condition and/or different dosages of SELFIE (e.g., face-to-face sessions, EMI components) as comparator. This further needs to adopt appropriate strategies for implementation of SELFIE as technology-enabled intervention in routine public and/or digital mental health services.

© 2023 Reininghaus U et al. *JAMA Psychiatry.*

eAppendix 2 Targeting self-esteem in ecological interventionist causal models and transdiagnostic intervention research

Building on an ecological interventionist causal model approach[13, 66], we adopted a strategy that targets candidate mechanisms, demonstrates that these are modifiable in vivo by an Ecological Momentary Intervention, and examines subsequent effects on the outcome. We have previously argued that the benefits of this approach, applied in the context of randomized controlled trials, are at least two-fold: first, this approach allows us to investigate important criteria for establishing causality such as association, temporal order, experimental evidence, and sole plausibility outside the research lab, in daily life. Second, it provides robust evidence whether the experimental manipulation method (i.e., the EMI) reflects an efficacious technique for prevention and intervention in individuals' daily living environments. Given previous research suggested that self-esteem is an important candidate mechanism in pathways to adverse mental health outcomes[67, 68] (including in daily life[38, 39, 69-71]) and provided evidence in support of 'association' as one important criterion for establishing causality, self-esteem reflected a promising intervention mechanism for the current trial adopting an ecological interventionist causal model approach. Notably, our findings may be viewed partially supportive of this approach, as, first, they provide experimental evidence on significant and sustained improvements in the primary outcome of global self-esteem of moderate effect size, corroborated by effect sizes suggestive of beneficial effects in secondary outcomes on positive and negative self-esteem, schematic self-beliefs, and, importantly, momentary self-esteem in daily life. Second, while no improvements were evident in observer-rated symptoms and functioning, effect sizes signaled beneficial effects for self-reported general psychopathology and quality of life as important subsequent outcomes. In short, our findings provide experimental evidence on modifying self-esteem as target mechanism (in vivo) and important subsequent outcomes. Overall, the current study reflects the first confirmatory trial of the ecological interventionist causal model approach and, coupled with our recently published EMIcompass trial[72], contributes more generally to demonstrating that the ecological interventionist causal model approach reflects an important translational strategy from mechanism to intervention and from the lab/clinic to daily living environments that may substantially contribute to ecological translation in public mental health provision. In a next step, we now need to formally test, in secondary (mediation) analyses of the current trial, including data on long-term effects at 18- and 24-month follow-up, the temporal order of experimental manipulation/exposure (i.e., SELFIE), putative mechanism (i.e., self-esteem), and subsequent outcomes (i.e., general psychopathology, quality of life), which will, in turn, allow for clarifying the role of self-esteem as transdiagnostic target mechanism and/or outcome.

The evaluation of interventions targeting transdiagnostic mechanisms across various diagnostic entities and severity levels of mental health problems face the challenge of selecting outcome measures that

are genuinely transdiagnostic in nature. This applies in particular to clinical outcomes that require measures with sufficient bandwidth cutting across dimensions and categories of diagnostic entities and capture relevant variance across clinical stages (e.g., in terms of frequency, severity etc.). While important work to address this challenge of identifying transdiagnostic symptom measures is under way[73], current studies need to draw on already available and validated scales. While the BPRS and K10 have been recommended for capturing clinical outcomes in youth[25], which informed our choice as outcome measures in the current study, it has not been designed to cover all relevant domains of early pluripotent or extended transdiagnostic phenotypes and their symptomatic outcomes. We urgently need to address this challenge for the field of prevention and early intervention to be able to progress to target transdiagnostic mechanisms and intervene at an early point to prevent enduring adult mental health problems in youth.

© 2023 Reininghaus U et al. *JAMA Psychiatry.*

eReferences

1. Bernstein DP, Fink L. *Childhood Trauma Questionnaire: a retrospective self-report manual.* San Antonio (Texas): The Psychological Corporation; 1998.
2. Scher CD, Stein MB, Asmundson GJ, McCreary DR, Forde DR. The childhood trauma questionnaire in a community sample: psychometric properties and normative data. *J Trauma Stress.* 2001;14(4):843-857.
3. Wright KD, Asmundson GJ, McCreary DR, Scher C, Hami S, Stein MB. Factorial validity of the Childhood Trauma Questionnaire in men and women. *Depress Anxiety.* 2001;13(4):179-183.
4. Schäfer M, Korn S, Smith PK, Hunter SC, Mora-Merchán JA, Singer MM, Meulen K. Lonely in the crowd: Recollections of bullying. *British Journal of Developmental Psychology.* 2004;22(3):379-394.
5. Bifulco A, Brown GW, Harris TO. Childhood Experience of Care and Abuse (CECA): a retrospective interview measure. *J Child Psychol Psychiatry.* 1994;35(8):1419-1435.
6. Schmitt P, Allik J. Simultaneous administration of the Rosenberg self-esteem scale in 53 nations: Exploring the universal and culture specific features of global self-esteem. *Journal of Personality and Social Psychology.* 2009;89:623-642.
7. Staring AB, van den Berg DP, Cath DC, Schoorl M, Engelhard IM, Korrelboom CW. Self-esteem treatment in anxiety: A randomized controlled crossover trial of Eye Movement Desensitization and Reprocessing (EMDR) versus Competitive Memory Training (COMET) in patients with anxiety disorders. *Behav Res Ther.* 2016;82:11-20.
8. Dinesen B, Nonnecke B, Lindeman D, Toft E, Kidholm K, Jethwani K, Young HM, Spindler H, Oestergaard CU, Southard JA, Gutierrez M, Anderson N, Albert NM, Han JJ, Nesbitt T. Personalized Telehealth in the Future: A Global Research Agenda. *J Med Internet Res.* 2016;18(3):e53.
9. Heron KE, Smyth JM. Ecological momentary interventions: incorporating mobile technology into psychosocial and health behaviour treatments. *Br J Health Psychol.* 2010;15(Pt 1):1-39.
10. Myin-Germeys I, Klippel A, Steinhart H, Reininghaus U. Ecological momentary interventions in psychiatry. *Curr Opin Psychiatry.* 2016;29(4):258-263.
11. Myin-Germeys I, van Aubel E, Vaessen T, Steinhart H, Klippel A, Lafit G, Viechtbauer W, Batink T, van Winkel R, van der Gaag M, van Amelsvoort T, Marcelis M, Schirmbeck F, de Haan L, Reininghaus U. Efficacy of Acceptance and Commitment Therapy in Daily Life in Early Psychosis: Results from the Multi-Center INTERACT Randomized Controlled Trial. *Psychother Psychosom.* 2022:1-13.
12. Rauschenberg C, Boecking B, Paetzold I, Schruers K, Schick A, van Amelsvoort T, Reininghaus U. A compassion-focused ecological momentary intervention for enhancing resilience in help-seeking youth: uncontrolled pilot study. *JMIR Ment Health.* 2021;8(8):e25650.
13. Reininghaus U, Depp CA, Myin-Germeys I. Ecological interventionist causal models in psychosis: targeting psychological mechanisms in daily life. *Schizophr Bull.* 2016;42(2):264-269.
14. Reininghaus U, Klippel A, Steinhart H, Vaessen T, van Nierop M, Viechtbauer W, Batink T, Kasanova Z, van Aubel E, van Winkel R, Marcelis M, van Amelsvoort T, van der Gaag M, de Haan L, Myin-Germeys I. Efficacy of Acceptance and Commitment Therapy in Daily Life (ACT-DL) in early psychosis: study protocol for a multi-centre randomized controlled trial. *Trials.* 2019;20(1):769.
15. Reininghaus U, Paetzold I, Rauschenberg C, Hirjak D, Banaschewski T, Meyer-Lindenberg A, Boehnke JR, Boecking B, Schick A. Effects of a Novel, Transdiagnostic Ecological Momentary Intervention for Prevention, and Early Intervention of Severe Mental Disorder in Youth (EMIcompass): Findings From an Exploratory Randomized Controlled Trial. *Schizophr Bull.* 2023;49(3):592-604.
16. De Neef M. *Build Your Confidence with CBT: 6 Simple Steps to be Happier, More Successful, and Fulfilled*: Open University Press; 2016.

© 2023 Reininghaus U et al. *JAMA Psychiatry.*

17. Postma MR, Hoes, I., Daemen, M., van Amersvoort, T. & Reininghaus, U. SELFIE, a transdiagnostic ecological momentary intervention for improving self-esteem in youth exposed to childhood adversity – structure of the intervention. *Maastricht University: Maastricht, the Netherlands.* 2019.
18. Rosenberg M. *Society and the adolescent self-image.* . 10th ed. Princeton, NJ: Princeton University Press; 1965.
19. Everaert J, Koster EHW, Schacht R, De Raedt R. Evaluatie van de psychometrische eigenschappen van de Rosenberg zelfwaardeschaal in een poliklinische psychiatrische populatie. *Gedragstherapie.* 2010;43:307-317.
20. Horvath AO, Greenberg LS. Development and validation of the Working Alliance Inventory. *Journal of counseling psychology.* 1989;36(2):223.
21. Lecomte T, Corbiere M, Laisne F. Investigating self-esteem in individuals with schizophrenia: relevance of the Self-Esteem Rating Scale-Short Form. *Psychiatry Res.* 2006;143(1):99-108.
22. Fowler D, Freeman D, Smith B, Kuipers E, Bebbington P, Bashforth H, Coker S, Hodgekins J, Gracie A, Dunn G, Garety P. The Brief Core Schema Scales (BCSS): psychometric properties and associations with paranoia and grandiosity in non-clinical and psychosis samples. *Psychol Med.* 2006;36(6):749-759.
23. Watson D, Clark LA, Tellegen A. Development and validation of brief measures of positive and negative affect: the PANAS scales. *J Pers Soc Psychol.* 1988;54(6):1063-1070.
24. Kessler RC, Andrews G, Colpe LJ, Hiripi E, Mroczek DK, Normand SL, Walters EE, Zaslavsky AM. Short screening scales to monitor population prevalences and trends in non-specific psychological distress. *Psychol Med.* 2002;32(6):959-976.
25. Kwan B, Rickwood DJ. A systematic review of mental health outcome measures for young people aged 12 to 25 years. *BMC Psychiatry.* 2015;15:279.
26. Derogatis LR. *SCL-90-R, administration, scoring & procedures manual-I for the R(evised) version*. Baltimore, MD: Johns Hopkins University School of Medicine; 1977.
27. Lukoff D, Liberman RP, Nuechterlein KH. Symptom monitoring in the rehabilitation of schizophrenic patients. *Schizophr Bull.* 1986;12(4):578-602.
28. Overall JE, Hollister LE, Pichot P. Major psychiatric disorders. A four-dimensional model. *Arch Gen Psychiatry.* 1967;16(2):146-151.
29. Whoqol Group. Development of the World Health Organization WHOQOL-BREF quality of life assessment. *Psychological medicine.* 1998;28(3):551-558.
30. Goldman HH, Skodol AE, Lave TR. Revising axis V for DSM-IV: a review of measures of social functioning. *Am J Psychiatry.* 1992;149(9):1148-1156.
31. Birchwood M, Smith J, Cochrane R, Wetton S, Copestake S. The Social Functioning Scale. The development and validation of a new scale of social adjustment for use in family intervention programmes with schizophrenic patients. *Br J Psychiatry.* 1990;157:853-859.
32. Myin-Germeys I, van Os J, Schwartz JE, Stone AA, Delespaul PA. Emotional reactivity to daily life stress in psychosis. *Arch Gen Psychiatry.* 2001;58(12):1137-1144.
33. Reininghaus U, Gayer-Anderson C, Valmaggia L, Kempton MJ, Calem M, Onyejiaka A, Hubbard K, Dazzan P, Beards S, Fisher HL, Mills JG, McGuire P, Craig TK, Garety P, van Os J, Murray RM, Wykes T, Myin-Germeys I, Morgan C. Psychological processes underlying the association between childhood trauma and psychosis in daily life: an experience sampling study. *Psychol Med.* 2016;46(13):2799-2813.
34. Reininghaus U, Kempton MJ, Valmaggia L, Craig TK, Garety P, Onyejiaka A, Gayer-Anderson C, So SH, Hubbard K, Beards S, Dazzan P, Pariante C, Mondelli V, Fisher HL, Mills JG, Viechtbauer W, McGuire P, van Os J, Murray RM, Wykes T, Myin-Germeys I, Morgan C. Stress sensitivity, aberrant salience, and threat anticipation in early psychosis: an experience sampling study. *Schizophr Bull.* 2016;42(3):712-722.
35. Ader L, Schick A, Simons C, Delespaul P, Myin-Germeys I, Vaessen T, Reininghaus U. Positive Affective Recovery in Daily Life as a Momentary Mechanism Across Subclinical and Clinical

© 2023 Reininghaus U et al. *JAMA Psychiatry.*

Stages of Mental Disorder: Experience Sampling Study. *JMIR Ment Health.* 2022;9(11):e37394.

36. De Calheiros Velozo J, Lafit G, Viechtbauer W, van Amelsvoort T, Schruers K, Marcelis M, Goossens L, Simons CJP, Delespaul P, Claes S, Myin-Germeys I, Vaessen T. Delayed affective recovery to daily-life stressors signals a risk for depression. *J Affect Disord.* 2023;320:499-506.

37. Vaessen T, Viechtbauer W, van der Steen Y, Gayer-Anderson C, Kempton MJ, Valmaggia L, McGuire P, Murray R, Garety P, Wykes T, Morgan C, Lataster T, Lataster J, Collip D, Hernaus D, Kasanova Z, Delespaul P, Oorschot M, Claes S, Reininghaus U, Myin-Germeys I. Recovery from daily-life stressors in early and chronic psychosis. *Schizophr Res.* 2019;213:32-39.

38. Thewissen V, Bentall RP, Lecomte T, van Os J, Myin-Germeys I. Fluctuations in self-esteem and paranoia in the context of daily life. *J Abnorm Psychol.* 2008;117(1):143-153.

39. Thewissen V, Bentall RP, Oorschot M, J AC, van Lierop T, van Os J, Myin-Germeys I. Emotions, self-esteem, and paranoid episodes: an experience sampling study. *Br J Clin Psychol.* 2011;50(2):178-195.

40. Myin-Germeys I, Oorschot M, Collip D, Lataster J, Delespaul P, van Os J. Experience sampling research in psychopathology: opening the black box of daily life. *Psychol Med.* 2009;39(9):1533-1547.

41. Reininghaus U, Daemen, M., Schick, A., Postma, M.R., Volbragt, N., Lindauer, R., Hoes - van der Meulen, I., Nieman, D., Delespaul, P., Breedvelt, J., van der Gaag, M., Viechtbauer, W., van den Berg, D., Bockting, C., van Amelsvoort, T. The effects of a transdiagnostic ecological momentary intervention for improving self-esteem (SELFIE) in youth exposed to childhood adversity: data analysis plan for a multi-center randomized controlled trial. OSF registries.; 2022-08-10:https://osf.io/8e4bc.

42. StataCorp. *Stata Statistical Software: Release 16.* College Station, TX: StataCorp LP; 2021.

43. Kolubinski DC, Frings D, Nikcevic AV, Lawrence JA, Spada MM. A systematic review and meta-analysis of CBT interventions based on the Fennell model of low self-esteem. *Psychiatry Res.* 2018;267:296-305.

44. Richardson AM, Welsh AH. Robust restricted maximum likelihood in mixed linear models. *Biometrics.* 1995:1429-1439.

45. StataCorp L. Stata survival analysis reference manual. 2017.

46. Little RJ, Rubin DB. *Statistical analysis with missing data.* Vol 793: John Wiley & Sons; 2019.

47. Twisk JW, Rijnhart JJ, Hoekstra T, Schuster NA, Ter Wee MM, Heymans MW. Intention-to-treat analysis when only a baseline value is available. *Contemp Clin Trials Commun.* 2020;20:100684.

48. White IR, Carpenter J, Horton NJ. Including all individuals is not enough: lessons for intention-to-treat analysis. *Clin Trials.* 2012;9(4):396-407.

49. Luers B, Klasnja P, Murphy S. Standardized Effect Sizes for Preventive Mobile Health Interventions in Micro-randomized Trials. *Prev Sci.* 2019;20(1):100-109.

50. Xiao Z, Kasim A, Higgins S. Same difference? Understanding variation in the estimation of effect sizes from educational trials. *International Journal of Educational Research.* 2016;77:1-14.

51. Hoffman L, Stawski RS. Persons as contexts: Evaluating between-person and within-person effects in longitudinal analysis. *Res Hum Dev. .* 2009;6:97-120.

52. Bijleveld CC, Leo, J. T., Leo, J., Mooijaart, A., Van Der Van Der, W. A., Van Der Leeden, R., & Van Der Burg, E. . *Longitudinal data analysis: Designs, models and methods.* . London: Sage; 1998.

53. Daemen M, Postma MR, Lindauer R, Hoes-van der Meulen I, Nieman D, Delespaul P, Breedvelt JJF, van der Gaag M, Viechtbauer W, Schruers K, van den Berg D, Bockting C, van Amelsvoort T, Reininghaus U. Efficacy of a transdiagnostic ecological momentary intervention for improving self-esteem (SELFIE) in youth exposed to childhood adversity: study protocol for a multi-center randomized controlled trial. *Trials.* 2021;22(1):641.

54. Ebert DD, Van Daele T, Nordgreen T, Karekla M, Compare A, Zarbo C, Brugnera A, Øverland S, Trebbi G, Jensen KL. Internet-and mobile-based psychological interventions: applications, efficacy, and potential for improving mental health. *European Psychologist.* 2018.
55. Rauschenberg C, Rus-Calafell M, Reininghaus U, Valmaggia L. Digital Interventions for Psychosis. In: Asmundson GJ, ed. *Reference Module in Neuroscience and Biobehavioral Psychology*. 2nd ed. Amsterdam, Netherlands: Elsevier Ltd.; 2022.
56. Rauschenberg C, Schick A, Hirjak D, Seidler A, Paetzold I, Apfelbacher C, Riedel-Heller SG, Reininghaus U. Evidence Synthesis of Digital Interventions to Mitigate the Negative Impact of the COVID-19 Pandemic on Public Mental Health: Rapid Meta-review. *Journal of medical Internet research.* 2021;23(3):e23365.
57. Myin-Germeys I, Klippel A, Steinhart H, Reininghaus U. Ecological momentary interventions in psychiatry. *Current opinion in psychiatry.* 2016;29(4):258-263.
58. Myin-Germeys I, Birchwood M, Kwapil T. From environment to therapy in psychosis: a real-world momentary assessment approach. *Schizophr Bull.* 2011;37(2):244-247.
59. Reininghaus U. Ecological momentary interventions in psychiatry: the momentum for change in daily social context. *Psychiatrische Praxis.* 2018;45(2):59.
60. Patrick K, Intille SS, Zabinski MF. An ecological framework for cancer communication: implications for research. *J Med Internet Res.* 2005;7(3):e23.
61. Myin-Germeys I, Kasanova Z, Vaessen T, Vachon H, Kirtley O, Viechtbauer W, Reininghaus U. Experience sampling methodology in mental health research: new insights and technical developments. *World Psychiatry.* 2018;17(2):123-132.
62. Palmier-Claus JE, Myin-Germeys I, Barkus E, Bentley L, Udachina A, Delespaul PA, Lewis SW, Dunn G. Experience sampling research in individuals with mental illness: reflections and guidance. *Acta Psychiatr Scand.* 2011;123(1):12-20.
63. Reininghaus U, Depp CA, Myin-Germeys I. Ecological interventionist causal models in psychosis: targeting psychological mechanisms in daily life. *Schizophrenia bulletin.* 2016;42(2):264-269.
64. Koppe G, Guloksuz S, Reininghaus U, Durstewitz D. Recurrent neural networks in mobile sampling and intervention. *Schizophrenia bulletin.* 2019;45(2):272-276.
65. Rauschenberg C, Schick A, Goetzl C, Roehr S, Riedel-Heller SG, Koppe G, Durstewitz D, Krumm S, Reininghaus U. Social isolation, mental health, and use of digital interventions in youth during the COVID-19 pandemic: A nationally representative survey. *Eur Psychiatry.* 2021;64(1):e20.
66. Kendler KS, Campbell J. Interventionist causal models in psychiatry: repositioning the mind-body problem. *Psychol Med.* 2009;39(6):881-887.
67. Brown GW, Craig TK, Harris TO, Handley RV. Parental maltreatment and adulthood cohabiting partnerships: a life-course study of adult chronic depression--4. *J Affect Disord.* 2008;110(1-2):115-125.
68. Garety PA, Bebbington P, Fowler D, Freeman D, Kuipers E. Implications for neurobiological research of cognitive models of psychosis: a theoretical paper. *Psychol Med.* 2007;37(10):1377-1391.
69. Daemen M, van Amelsvoort T, Group I, Reininghaus U. Self-esteem and psychosis in daily life: An experience sampling study. *J Psychopathol Clin Sci.* 2022;131(2):182-197.
70. Postma MR, van Amelsvoort T, Myin-Germeys I, Gayer-Anderson C, Kempton MJ, Valmaggia L, McGuire P, Murray RM, Garety P, Wykes T, Morgan C, Reininghaus U. Across the continuum: Associations between (fluctuations in) momentary self-esteem and psychotic experiences. *Schizophr Res.* 2021;238:188-198.
71. Daemen M. *Sampling the self: investigating and improving self-esteem in pathways to psychopathology*. Maastricht: Department of Psychiatry and Neuropsychology, Maastricht University; 2022.
72. Reininghaus U, Paetzold I, Rauschenberg C, Hirjak D, Banaschewski T, Meyer-Lindenberg A, Boehnke JR, Boecking B, Schick A. Effects of a Novel, Transdiagnostic Ecological Momentary

Intervention for Prevention, and Early Intervention of Severe Mental Disorder in Youth (EMIcompass): Findings From an Exploratory Randomized Controlled Trial. *Schizophr Bull.* 2023.

73. Krueger RF, Kotov R, Watson D, Forbes MK, Eaton NR, Ruggero CJ, Simms LJ, Widiger TA, Achenbach TM, Bach B, Bagby RM, Bornovalova MA, Carpenter WT, Chmielewski M, Cicero DC, Clark LA, Conway C, DeClercq B, DeYoung CG, Docherty AR, Drislane LE, First MB, Forbush KT, Hallquist M, Haltigan JD, Hopwood CJ, Ivanova MY, Jonas KG, Latzman RD, Markon KE, Miller JD, Morey LC, Mullins-Sweatt SN, Ormel J, Patalay P, Patrick CJ, Pincus AL, Regier DA, Reininghaus U, Rescorla LA, Samuel DB, Sellbom M, Shackman AJ, Skodol A, Slade T, South SC, Sunderland M, Tackett JL, Venables NC, Waldman ID, Waszczuk MA, Waugh MH, Wright AGC, Zald DH, Zimmermann J. Progress in achieving quantitative classification of psychopathology. *World Psychiatry.* 2018;17(3):282-293.

© 2023 Reininghaus U et al. *JAMA Psychiatry.*
