# Supplementary material for: Transdiagnostic Ecological Momentary Intervention for Improving Self-Esteem in Youth Exposed to Childhood Adversity: The SELFIE Randomized Clinical Trial
Source: JAMA Psychiatry. 2023 Nov 29;81(3):227–39. doi: 10.1001/jamapsychiatry.2023.4590 (PMC10687716; doi:10.1001/jamapsychiatry.2023.4590)
Supplement: Supplement 3. — Data Sharing Statement. [file jamapsychiatry-e234590-s003.pdf]

Data Sharing Statement

Reininghaus. Transdiagnostic ecological momentary intervention for improving self-esteem in youth exposed to childhood adversity: the SELFIE randomized clinical trial. *JAMA Psychiatry*. Published online November 29, 2023. doi:10.1001/jamapsychiatry.2023.4590

Data
Data available:
Yes

Data types:
Deidentified participant data

How to access data:
The datasets generated and/or analyzed during the current study will be available in anonymized form from the corresponding author (UR) on reasonable request, subject to review, following the publication of results.

When available:
With publication

Document types:
None

Who can access the data:
Researchers whose proposed use of the data has been approved

Types of analyses:
For any secondary analyses that do not overlap with planned analyses of the SELFIE research team

Mechanisms of data availability:
After approval of a proposal, with a signed data access agreement

Any additional restrictions:
The datasets generated and/or analyzed during the current study will be available in anonymized form from the corresponding author (UR) on reasonable request, subject to review, following the publication of results.
